# Genexpi: a toolset for identifying regulons and validating gene regulatory networks using time-course expression data

**DOI:** 10.1186/s12859-018-2138-x

**Published:** 2018-04-13

**Authors:** Martin Modrák, Jiří Vohradský

**Affiliations:** Institute of Microbiology of the Czech Academy of Sciences, Vídeňská, 1083 Prague, Czech Republic

**Keywords:** Gene network inference, Transcription regulation, Time series, Cytoscape

## Abstract

**Background:**

Identifying regulons of sigma factors is a vital subtask of gene network inference. Integrating multiple sources of data is essential for correct identification of regulons and complete gene regulatory networks. Time series of expression data measured with microarrays or RNA-seq combined with static binding experiments (e.g., ChIP-seq) or literature mining may be used for inference of sigma factor regulatory networks.

**Results:**

We introduce Genexpi: a tool to identify sigma factors by combining candidates obtained from ChIP experiments or literature mining with time-course gene expression data. While Genexpi can be used to infer other types of regulatory interactions, it was designed and validated on real biological data from bacterial regulons. In this paper, we put primary focus on CyGenexpi: a plugin integrating Genexpi with the Cytoscape software for ease of use. As a part of this effort, a plugin for handling time series data in Cytoscape called CyDataseries has been developed and made available. Genexpi is also available as a standalone command line tool and an R package.

**Conclusions:**

Genexpi is a useful part of gene network inference toolbox. It provides meaningful information about the composition of regulons and delivers biologically interpretable results.

**Electronic supplementary material:**

The online version of this article (10.1186/s12859-018-2138-x) contains supplementary material, which is available to authorized users.

## Background

Uncovering the nature of gene regulatory networks is one of the core tasks of systems biology. Identifying direct regulons of sigma factors/transcription factors can be considered the basic element of this task. In fact a large portion of software for network inference is limited to such direct interactions (e.g., [1–3]). It has however been shown that using only one source of data for network inference (e.g., only CHIP-seq experiment) can be misleading and combining multiple sources is necessary [4].

Primary focus of this paper is on CyGenexpi – a plugin for the Cytoscape platform [5] that uses time-course gene expression data to discover regulons among candidate genes obtained from other sources (literature, database mining, or ChIP experiments). CyGenexpi can be also used for de-novo network inference, although this is less reliable. CyGenexpi is built on top of the Genexpi software package that provides the core functionality also as a command-line tool and an interface to the R language.

Genexpi is based on an ordinary differential equation model of gene expression introduced in [6]. In the model, the synthesis of new mRNA for a gene is determined by a non-linear (sigmoidal) transformation of the expression of its regulators. The model also includes a per-gene decay rate of the mRNA, which is assumed to be constant.

While there are multiple tools for gene network inference from the command line or programming languages (see [7] for a recent review), there are currently, only two Cytoscape plugins for gene network inference: ARACNE [8] and Network BMA [9]. ARACNE is intended for steady-state expression data, while Network BMA handles time series, but assumes a simple linear model of regulation without regard to mRNA decay. CyGenexpi thus provides an alternative to Network BMA in that it builds on a non-linear model including decay.

A preliminary version of the method presented in this paper has been applied in our previous work [10]. The additional contribution of this paper is a) a polished and documented publicly available implementation of the method with well-defined API, b) improved workflow and software support for the workflow c) interfacing the method with Cytoscape and R and d) evaluation of the method on additional datasets. As Cytoscape does not natively support working with time series data, we also developed CyDataseries - a plugin for importing and handling time series and other forms of repeated measurements data in Cytoscape.

Both Genexpi and CyDataseries are imlemented in Java and are platform independent. Binaries, source code and documentation are available at http://github.com/cas-bioinf/genexpi/wiki/. The software is open source and licensed under LGPL version 3.

## Implementation

The core of Genexpi – the algorithm for fitting model parameters – is implemented in OpenCL, with a Java wrapper. Thanks to high portability of both Java and OpenCL, Genexpi can be executed on both GPUs and CPUs in any major operating system and has very good performance. There are currently three interfaces to Genexpi core: CyGenexpi (a Cytoscape plugin), a command-line interface and an R interface. In this section we describe the model and fitting method of Genexpi – the implementation of the interfaces is straightforward. Initial part of this section is taken from [10] and its supplementary material where we describe first use of Genexpi in practice. In addition we provide details of regularization and parameter fitting as well as further developments made to make the method usable by non-expert users, especially the semi-automatic evaluation of good fits and the “no change” and “constant synthesis” models.

### The model

Genexpi is based on an ordinary differential equation (ODE) model for gene regulation, inspired by the neural network formalism [6]. In this model the synthesis of new mRNA for a gene *z* controlled by set of *m* regulators *y*_*1*_*,..,y*_*m*_ (genes or any other regulatory influence) is determined by activation function *f(ρ(t))* of the regulatory input $\rho (t)={\sum}_{j=1..m}{w}_j{y}_j(t)+b$. Here *w*_*j*_ is the relative weight of regulator *y*_*j*_ and *b* is bias (inversely related to the regulatory influence that saturates the synthesis of the mRNA). In our case, *f* is the logistic soft-threshold function *f(x) = 1/(1 + e*^*-x*^*)*. The transcript level of *z* is then governed by the ODE:

1 $$ \frac{dz}{dt}={k}_1f\left(\rho \right)-{k}_2z $$

where *k*_*1*_ is related to the maximal level of mRNA synthesis and *k*_*2*_ represents the decay rate of the mRNA. Both *k*_*1*_ and *k*_*2*_ must be positive. The complete set of parameters for this model is thus *β = {k*_*1*_*, k*_*2*_*, b, w*_*1*_*,…, w*_*m*_*}*. Given *N* samples from a time series of gene expression taken at time points *t*_*1*_*, …, t*_*N*_ the inference task can be formalized as finding *β* that minimizes squared error with regularization*:*

2 $$ \widehat{\beta}=\underset{\beta }{\mathrm{argmin}}\left[\sum \limits_{i=1}^N{\left({\widehat{z}}_{\beta}\left({t}_i\right)-z\left({t}_i\right)\right)}^2+r\left(\beta \right)\right] $$

Here *z* is the observed expression profile, ${\widehat{z}}_{\beta }$ the solution to (1) given the parameter values *β* and the observed expression of *y*_*1*_*,..,y*_*m*_, and *r(β)* is the regularization term. The regularization term represents a prior probability distribution over *β* that gives preference to biologically interpretable values for *β* and is discussed in more detail below*.* Assuming Gaussian noise in the expression data, (2) is the maximum a posteriori estimate of *β*.

Our model is similar to that used by the Inferelator algorithm [1], although there are important differences: the Inferelator does not model decay (*k*_*2*_) – it assumes decay is always one. Further, Inferelator minimizes the error of the predicted derivative of the expression profile, while we minimize the prediction error for the actual integrated expression profile and introduce the regularization term.

### Smoothing the expression profiles

Since the expression data is noisy, Genexpi encourages smoothing the data prior to computation. We have had good results with linear regression of B-spline basis with degrees of freedom equal to approximately half the number of measurement points. By smoothing we get more robust results with respect to low frequency phenomena, but sacrifice our ability to discover high-frequency changes and regulations (oscillations with frequency comparable to the measurement interval are mostly suppressed). Further our experiments with fitting raw data or tight interpolations of the data (e.g. cubic spline with knots at all measurement points) have had little success in fitting even the profiles that were highly correlated, due to the amplified noise in the data. Smoothing of time series profiles has been used previously for network inference [11].

Further advantage of smoothing is that it lets us subsample the fitted curve at arbitrary resolution. The subsampling then allows us to integrate (1) accurately with the computationally cheap Euler method, making evaluation of the error function fast and easy to implement in OpenCL.

### Parameter fitting and regularization

Genexpi minimizes eq. 2 by simulated annealing. For each gene and candidate regulator set we execute 128 annealing runs with different initial parameter values. Using 128 runs was enough to achieve high replicability of the results. Annealing runs for the same target and regulator are executed on the same OpenCL compute unit, letting us to move all necessary data to local memory and thus increase efficiency. We use the XorShift1024* random generator [12] as a fast and high quality parallel source of randomness.

Note that in some cases, multiple vastly different combinations of parameters may yield almost identical regulatory profiles. For example, if the interval of attained regulatory input $\left(\underset{i=1..N}{\min}\rho \left({t}_i\right);\underset{i=1..N}{\max}\rho \left({t}_i\right)\right)$ lies completely on one of the tails of *f*, the activation function becomes approximately linear over the whole interval, so increasing the weights and decreasing bias while decreasing *k*_*1*_ yields a very similar ${\widehat{z}}_{\beta }$. To discriminate between those models and to force the parameters into biologically interpretable ranges, we introduce the regularization term *r(β).* In particular, we expect *k*_*1*_ smaller than the maximal expression level of the target gene (i.e., that maximal transcript level cannot be achieved in less than a unit time starting from zero), we put a bound on maximal steepness of the regulatory response: $\underset{t}{\max}\mid {w}_j{y}_j(t)\mid <10$ for all regulators *j* and we expect the regulatory input to come close to zero (the steepest point of the sigmoid function) for at least one time point: $\underset{t}{\min}\mid \rho (t)\mid <0.5$. For a suitable penalty function *γ*(*x*, *ω*) the regularization term becomes:

3 $$ r\left(\beta \right)=c\left(\gamma \left({k}_1,\underset{i=1..N}{\max }z\left({t}_i\right)\right)+\sum \limits_{j=1}^m\gamma \Big(\underset{i=1..N}{\max}\left|{w}_j{y}_j\left({t}_i\right)\right|,10\left)+\gamma \right(\underset{i=1..N}{\min}\left|\rho \left({t}_i\right)\right|,0.5\right) $$

where c is a constant governing the amount of regularization. In our work, the penalty for value *x > 0* and bound *ω* is:

4 $$ \gamma \left(x,\omega \right)=\left\{\begin{array}{c}0,\kern0.5em x\le \omega \\ {}{\left(\frac{x}{\omega }-1\right)}^2,\kern0.5em x>\omega \end{array}\right. $$

Minimizing *γ*(*x*, *ω*) is then the same as maximizing log-likelihood, assuming that *x* is distributed uniformly over *(0; ωx)* with some probability *p* and as *ωx* + *α|e|* with probability *(1 – p)* where e ∼ N(0, 1). In this interpretation, the probability *p* is uniquely determined by *c* in the regularization term and by choosing α such that the resulting density function is continuous.

We have empirically determined the best value of *c* to be approximately one tenth of the number of time points after smoothing. While without regularization, many of the inferred models contained implausible parameter values, regularization forced almost all of those parameters into given bounds - *r(β)* was zero for most models. At the same time the mean residual error of the models inferred with regularization differed by less than one part in hundred from models inferred without regularization.

### Evaluating good fits

To evaluate whether a fit is good, we have chosen a simple, but easily interpretable approach. The primary reason is that we intend to keep the human in the loop throughout the inference process and thus the human has to be able to understand the criteria intuitively. Since most published time series expression data is reported only as averages without any quantification of uncertainty, we let the user set the expected error margin based on their knowledge of the data. The error margin is determined by three parameters: absolute, relative and minimal error. These combine in a straightforward way to get an error margin for each time point, depending on the expression level *z(t)*:

5 $$ error(t)=\max \left\{{e}_{minimal},{e}_{absolute}+z(t){e}_{relative}\right\} $$

*Fit quality* is then the proportion of time points where the fitted profile is within the error margin of the measured profile. A fit is considered good if fit quality is above a given threshold (the default value is 0.8).

### No change and constant synthesis model

Prior to analyzing a gene as being regulated, we need to test for two baseline cases that would make any prediction useless. The obvious first case are genes that do not change significantly over the whole time range. Genes that do not change are excluded from further analysis as both regulators and targets as the Genexpi model contains no information in that case.

A slightly more complicated case is the constant synthesis model where we expect the mRNA synthesis to be constant over the whole time range:

6 $$ \frac{dz}{dt}={k}_1-{k}_2z $$

Note that this is the same as assuming there are 0 regulators. Since genes with constant synthesis could be fitted by any regulator by simply putting *w = 0*, and large *b,* those genes are excluded as targets. However, regulators that could be explained by constant synthesis are still analyzed, as there is meaningful information. Fitting the constant synthesis model is also done via simulated annealing in OpenCL.

For the putative regulations excluded this way, the correct interpretation is that the underlying dataset provides no evidence for or against such regulations. If there are biological justifications that the regulations should be visible in the data (e.g. that the regulatory effect should be larger than the measurement noise), it is possible to cautiously consider this as evidence against the regulations taking place.

## Results and discussion

In this section we describe the intended workflow for analysis with Genexpi and its user interface and then we discuss results of evaluation on real biological data.

The primary user interface for Genexpi is the CyGenexpi plugin for the Cytoscape software, but Genexpi can also be run directly from R and via a command line interface. For CyGenexpi, an important improvement over the Aracne or NetworkBMA Cytoscape plugins is the direct involvement of user in the process.

### Genexpi workflow

**Fig. 1:**
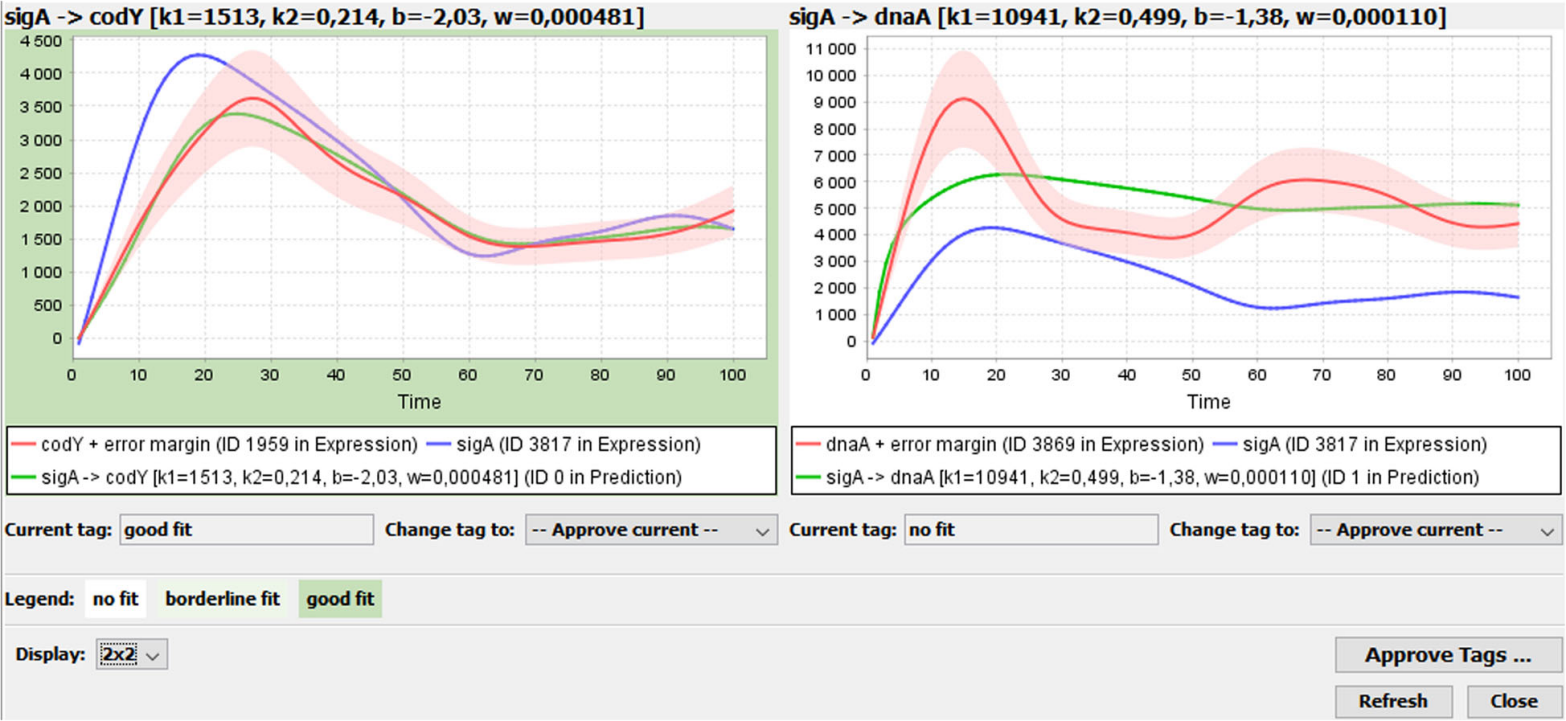
Human inspection of the model fits in CyGenexpi. The user is shown the profile of the regulator (blue) and target (red) as well as the best profile found by Genexpi (green). The red ribbon is the error margin of the measured profile. The algorithm classified the first profile as a good fit, while the second was considered implausible to be regulated. The user may however modify the classification based on their knowledge of the data and organism

The workflow for analysis with Genexpi is as follows:

1. Start with a network of putative regulations either obtained from database mining or experiments.
2. Import the time-course expression data and smooth them to provide a continuous curve.
3. Remove genes whose expression does not change significantly throughout the whole time-course.
4. Remove genes that could be modelled by the constant synthesis model.
5. Optional: Human inspection of the results of steps 3&4, possibly overriding the algorithm’s decisions.
6. Finding best parameters of the Genexpi model for each gene-regulator pair. The fitted models are then classified into good and bad fits. Good fits indicate that the regulation is plausible, while bad fits show that the regulation either does not take place or involves additional regulators.
7. Optional: Human inspection of the fits, possibly overriding the algorithm’s classification (shown in Fig. 1).

This workflow is completely covered by CyGenexpi with the help of CyDataseries in a simple wizard-style interface. Alternatively, the same workflow, but without human intervention can be run by a single function call in R. All interfaces also provide the user with the ability to run individual steps separately.

While Genexpi can include multiple regulators for a gene, we found this not very useful in practice, as even for relatively long expression time series (13 time points), an arbitrary pair of regulators is able to model the expression of a large fraction of all genes, increasing the false positive rate. CyGenexpi therefore currently does not expose GUI for using more than one regulator in the model. Using more regulators is however available for more advanced users via the command-line or R interfaces.

For CyGenexpi, the time series data is imported with CyDataseries from either a delimited text file or the SOFT format used in Gene Expression Omnibus.

While Genexpi can be used for de-novo regulon identification from time-series expression data only, high rate of false positives should be expected. The main reason is that in real biological data, multiple sigma factors may have similar expression profiles and Genexpi thus considers all genes regulated by one of the sigma factors as possibly regulated by all of the similar sigma factors. The evaluation in this paper therefore focuses on identifying the regulated genes among a set of plausible candidates. Nevertheless, the workflow for de-novo inference is almost the same as described above, only the initial network should contain a link from each investigated regulator to all other genes.

We evaluated Genexpi in three ways: 1) direct biological testing of the suggested regulatory relationships, 2) comparing the ability of Genexpi and other tools to reconstruct two literature-derived regulons and 3) measuring computing time required to process the data. The first part of the evaluation is taken from our previous work [10], while the latter two are new contributions.

### In-vitro biological evaluation

This section recapitulates the relevant results obtained with Genexpi, originally reported as a part of [10]. We performed a basic analysis of the predictive performance of Genexpi with the SigA regulon of Bacillus subtilis combined with the expression time series from GSE6865 [13]. We followed the Genexpi workflow outlined in the previous section, including evaluation of fits by human. Genexpi predicted 215 genes that were not known to be regulated by SigA as potential SigA targets. We selected 10 of those genes for in-vitro transcription assays.^1^ We found that 5 of them were SigA-dependent (for the remaining five, the regulation could not be excluded). More details of the SigA analysis can be found in the aforementioned paper. We have however excluded the SigA regulon from purely computational evaluation as the method was developed and tweaked for the SigA data and any comparison would thus be likely biased.

### Reconstructing bacterial regulons

To extend the biological evaluation from [10] and to better determine Genexpi’s performance in identifying regulons, we took two bacterial regulons from the literature: a) the SigB regulon of *B. subtilis* from Subtiwiki [14] as of January 2017 combined with the GSE6865 expression time series [13] and b) two versions of the SigR regulon of Streptomyces coelicolor: one derived with ChIP-chip [15] and the one determined via knockouts [16]. Both versions of the SigR regulon were combined with the GSE44415 expression time series [17].

**Fig. 2:**
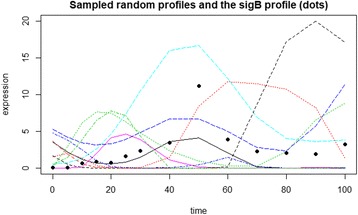
A sample of the random profiles tested against the SigB regulon. The dots represent the measured (not smoothed) profile of SigB

For each of the literature regulons we first exclude targets that were constant or had constant synthesis (steps 3&4 of the workflow) and determined how many of the remaining members were considered by Genexpi to be regulated by the respective sigma factor – these correspond to true positives. Then we generated a set of random expression profiles with similar magnitude and rate of change as the sigma factor. Inspired by [18] we draw random profiles from a Gaussian process with a squared exponential kernel with zero mean function, transformed to have positive values. See Fig. 2 for an example of the random profiles. We then tested how many targets were predicted to be regulated by this nonsensical profile – these correspond to false positives.

We consider testing a random regulator profile as a more reliable assessment than testing the complement of the literature-based regulon for two reasons. First, it is a better match for the intended Genexpi workflow, which starts with a set of candidate genes. Here, using a random profile for the regulator models the situation where the candidate list is wrong and we expect Genexpi to reject that there is regulatory influence on most genes. Second, the complement is usually composed of less characterized genes and there is little guarantee that the complement contains genes that are not regulated by the sigma factor. The complement may include genes that are regulated with the sigma factor, but were not annotated yet, and also genes that have expression profile similar to the profiles of the regulon of the analyzed sigma factor due to chance or non-regulatory interactions. Such profiles would be classified as false positives, while they in fact have nothing to do with the analyzed regulon and its sigma factor. Comparing the performance on regulon complement actually depends more on the uniqueness of the sigma factor profile than on the inference algorithm.

For this evaluation we ran Genexpi with default settings and without any human input. Complete code to reproduce all of the results for this and the following section is attached as an R notebook in Additional file 1.

For comparison, we performed the same analysis with TD-Aracne [19] – an extension of the frequently used Aracne algorithm designed for time series data. TD-Aracne was run both on the whole dataset at once and on each regulator-target pair separately. Running regulator-target pairs however had much worse performance than using the whole dataset, so those results are omitted here, but can be inspected in Additional file 1. We also compared the results for the whole regulon and for the subset of the regulon that was predicted by Genexpi, i.e. without the genes removed in steps 3&4 of the workflow.

For all analyses, we smoothed the raw data by linear regression over B-spline basis of order 3 with 3–10 degrees of freedom. TD-Aracne was tested with the raw data as well as the smoothed data subsampled to give lower number of equal-spaced time points as expected by TD-Aracne. For TD-Aracne we tested three methods of recovering the regulon from the inferred network over the full gene set: a) take only the genes that were marked as directly regulated by the sigma factor, b) take all genes connected by a directed path from the regulator and c) take all genes connected to the regulator. Variant a) had very low performance overall, among b) and c) we report the result more favorable to TD-Aracne. For the SigR regulon of Kim et al., the results were very similar when only the targets marked as having “strong” evidence were used. All results not shown here can be found in Additional file 1. See Table 1 for the main results.

**Table 1:**
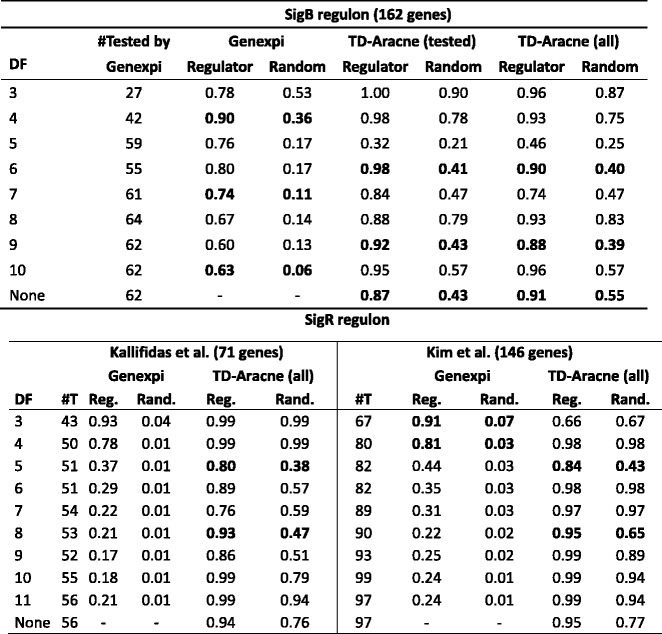
Main Evaluation Results

Results of Genexpi and TD-Aracne on the regulon reconstruction task. The “Regulator” column reports the proportion of predicted regulations by the true regulator, “Random” reports the proportion of predicted regulations by a random profile (averaged over 50 runs). The best results for each algorithm are highlighted in bold. TD-Aracne (tested) are results of TD-Aracne only on those genes not removed by Genexpi in steps 3&4 of the workflow. The “tested” variant is not reported for the SigR regulon as the results are very similar to those on all genes. The DFs column contains the degrees of freedom for the spline, “#T” stands for “Number of genes tested by Genexpi”, “Reg.” for “Regulator” and “Rand.” for “Random”

In the SigB regulon, the Genexpi performs slightly better than TD-Aracne. While TD-Aracne (in multiple settings) confirms almost all of the literature regulon while rejecting over half of the regulations by a random profile, Genexpi using spline with 4 degrees of freedom rejects two thirds of random regulations while also recovering 90% of the literature regulon. Moreover, Genexpi has the advantage of allowing for a sensitivity/specificity tradeoff by choosing the degree of freedom for the spline – with high degrees of freedom, almost all random regulations are rejected while still recovering majority of the literature regulon. The performance of TD-Aracne varied unexpectedly with the chosen degree of freedom. We also see, that running TD-Aracne with smoothed data and removing no change and constant synthesis genes as in Genexpi workflow, allows for only slight improvements for the performance of TD-Aracne over running directly with the raw data (as TD-Aracne is designed to work).

For both variants of the SigR regulon, TD-Aracne mostly found little difference between the literature based and random regulons. The few cases of better performance by TD-Aracne occurred unpredictably with certain smoothing of the data. At the same time, Genexpi was rarely misled by the random regulations and recovered large fractions of the literature regulon while behaving consistently: the proportion of both true and random regulations grows with more aggressive smoothing (less degrees of freedom).

### Computing time required

For analysis of computing time, Genexpi was run on a mid-tier GPU (Asus Radeon RX 550) and TD-Aracne on an upper-level CPU (Intel i7 6700 K). Both algorithms were run on a Windows 10 workstation with only basic precautions to prevent other process from perturbing the system load. The numbers reported should therefore not be considered benchmarks but rather an informative estimate of the computing time during a normal analysis workflow. The results are shown in Table 2 and indicate that Genexpi was fast enough to be run repeatedly on commodity hardware with TD-Aracne being slower, but still fast enough for most practical use cases.

**Table 2:** Computing time [s] required for a single inference run on the given regulon

	SigB	SigR Kallifidas et al.	SigR Kim et al.
Genexpi	26	109	150
TD-Aracne	447	184	446

Time taken to compute a possible regulations for a single regulon. All of the results were averaged across both the runs with the actual regulator profile and the runs with a randomly generated profile. All times in seconds

### Reconstructing eukaryotic regulons

While Genexpi was designed for bacterial regulons, we also tested its performance on eukaryotic data, in particular the time series of gene expression throughout the cell cycle of *Saccharomyces cerevisiae* [20], deposited as GDS38. We chose the same 8 transcription factors regulating the cell cycle as in our previous work [21] and downloaded their regulons from the YEASTRACT database (as of 2018–02-09) [22]. We used spline with 6 degrees of freedom to smooth the data and interpolate missing values. After excluding constant and constant synthesis targets (steps 3&4 of the workflow), we selected 30 targets for each gene at random to reduce computational burden. We then proceeded as in the bacterial regulons evaluation by generating random profiles and comparing recovered regulations by both Genexpi and TD-Aracne across the measured regulator profiles and 20 random profiles. The results are shown in Table 3.

**Table 3:** Evaluation results for *S. cerevisiae*

Transcription factor	Genexpi		TD-ARACNE	
	Regulator	Random	Regulator	Random
FKH1	0.30	0.24	0.00	0.14
FKH2	0.37	0.22	0.45	0.08
MCM1	0.33	0.18	0.65	0.20
NDD1	0.42	0.21	0.00	0.00
ACE2	0.40	0.28	0.52	0.33
MBP1	0.13	0.21	0.39	0.05
SWI4	0.23	0.16	0.48	0.10
SWI6	0.10	0.20	0.00	0.04

Results of Genexpi and TD-Aracne on the eukaryotic regulon reconstruction task. The “Regulator” column reports the proportion of predicted regulations by the true regulator, “Random” reports the proportion of predicted regulations by a random profile (averaged over 20 runs)

In this case, the signal is weaker than in the prokaryotes, which is not unexpected given the increased complexity of eukaryotic regulation. Genexpi gives the worst (undistinguishable from random) results for MBP1, SWI4 and SWI6, which are known to regulate in complexes and thus break the model expected by Genexpi. Interestingly, TD-Aracne is able to determine some of those regulations. For the other genes, Genexpi provides consistent, but weak information while TD-Aracne provides strong signal for some genes, while performing very poorly on the others.

The full code to reproduce the analysis can be found in Additional file 1.

### Future work

The Genexpi workflow was kept deliberately simple, but this involves some inaccuracies. Most notably, Genexpi masks uncertainty in the data and uses multiple hard thresholds. Following [18] that use a similar model of gene regulation in a fully Bayesian setting, we want to extend Genexpi to handle uncertainty explicitly and provide full posterior probability distributions for the quantities of interest.

## Conclusions

Our evaluation has shown that Genexpi is a useful part of a bioinformatician’s toolbox for uncovering and/or validating regulons in biological systems. Genexpi was designed for bacterial regulons, but can be – with caution – employed also for eukaryotic data. It also provides transparent results and – unlike other similar programs - lets the human to stay in the loop and apply expert knowledge when necessary. The parameters of the fitted models are biologically interpretable and thus can guide design of future experiments. Time-series expression data cannot in principle provide complete information about the regulatory interactions taking place and Genexpi is therefore best used as one of multiple sources of insight about a biological system.

Genexpi is equipped with both simple point&click interface for the Cytoscape application and with R and command-line interfaces for advanced users.

## List of mathematical notation

**Table:** 

Symbol	Meaning
*k*_*1*_	synthesis rate of a gene at full activation
*k*_*2*_	decay rate of a gene
*w*_*i*_	weight of regulatory influence of putative regulator *I* on the gene
*b*	bias of the activation function
***ρ***(***t***)	regulatory response (weighed sum of regulator profiles) as a function of time
*f*	activation function (logistic sigmoid in our case)
***β***	vector of all model parameters
*z(t)*	measured/smoothed mRNA levels of gene as a function of time
${\widehat{\boldsymbol{z}}}_{\boldsymbol{\beta}}$	mRNA levels estimated by a model with parameter vector *β*
*t*	time
*N*	number of time points
*y*_*i*_ *(t)*	mRNA level of i-th regulator as a function of time
*m*	number of regulators

## Additional file

Additional file 1: evaluation.zip - an archive containing: • evaluation.Rmd – R Markdown notebook (best used with RStudio, https://www.rstudio.com/) to reproduce the evaluation on bacterial regulons in this paper. evaluation.nb.html – Compiled version of evaluation.Rmd for easy reading, including stored results produced by running all the code. • evaluation_sacharomyces.Rmd – R Markdown notebook to reproduce the evaluation on *Sacharomyces* data. • evaluation_sacharomyces.nb.html – Compiled version of evaluation_sacharomyces.Rmd, including stored results produced by running all the code.

## Footnotes

1 Transcription of the gene within a solution with *SigA* present was compared to transcription without SigA (negative control) and transcription from a known strong *SigA*-dependent promoter (positive control).
