# Supplementary material for: Genexpi: a toolset for identifying regulons and validating gene regulatory networks using time-course expression data
Source: BMC Bioinformatics. 2018 Apr 13;19:137. doi: 10.1186/s12859-018-2138-x (PMC5899412; doi:10.1186/s12859-018-2138-x)
Supplement: Supplementary file 1 — evaluation.zip - an archive containing: • evaluation.Rmd – R Markdown notebook (best used with RStudio, https://www.rstudio.com/) to reproduce the evaluation on bacterial regulons in this paper. evaluation.nb.html – Compiled version of evaluation.Rmd for easy reading, including stored results produced by running all the code. • evaluation_sacharomyces.Rmd – R Markdown notebook to reproduce the evaluation on Sacharomyces data. • evaluation_sacharomyces.nb.html – Compiled version of evaluation_sacharomyces.Rmd, including stored results produced by running all the code. [file 12859_2018_2138_MOESM1_ESM.zip › evaluation.nb.html]

Genexpi evaluation


Code 

- Show All Code
- Hide All Code
- Download Rmd

# Genexpi evaluation

This R notebook performs the evaluation of Genexpi against TD-Aracne on regulon identifiaction tasks. Note that the full evaluation can take over a week. If you want to do quick rerun to understand what is going on, you can alter the `sigBRegulonNames`, `sigRRegulon_Kallifidas`, `sigRRegulon_Kim` and , `sigRRegulon_Kim_Strong` variables to contain just a few genes, which should give you answers in reasonable time.

# Setting up

We will keep a lot of intermediate results in the form of Java objects in memory, so let us increase the max memory. Please start the notebook in a fresh session.


```
  options(java.parameters = "-Xmx2048m")
  develRun = FALSE;
```


First we install and start all the required libraries.


```
  # Helper function to install packages if they are not available
  pkgTest <- function(x)
  {
    if (!require(x,character.only = TRUE))
    {
      install.packages(x,dep=TRUE)
        if(!require(x,character.only = TRUE)) stop("Package not found")
    }
  }

  # Install and load Genexpi from GitHub vie devtools
  if(!develRun) {
    pkgTest("devtools")
    install_github("cas-bioinf/genexpi", ref = "bmc-release", subdir="rpackage")
  }

  # Install and load the packages required for evaluation against TD-Aracne
  source("https://bioconductor.org/biocLite.R")
  biocLite(c("Biobase","TDARACNE", "RBGL"))
  library(Biobase)
  library(TDARACNE)
  library(RBGL)
  
  pkgTest("splines")
  pkgTest("foreach")
  pkgTest("doParallel")
  pkgTest("rJava")
  pkgTest("ggplot2")
```


Genexpi relies on OpenCL, so we need to check the available OpenCL devices:


```
listOpenCLDevices()
```


```
[1] "Intel(R) HD Graphics 530 (Intel(R) OpenCL)"                                          
[2] "Intel(R) Core(TM) i7-6700K CPU @ 4.00GHz (Intel(R) OpenCL)"                          
[3] "gfx804 (AMD Accelerated Parallel Processing)"                                        
[4] "Intel(R) Core(TM) i7-6700K CPU @ 4.00GHz (AMD Accelerated Parallel Processing)"      
[5] "Intel(R) Core(TM) i7-6700K CPU @ 4.00GHz (Experimental OpenCL 2.0 CPU Only Platform)"
[6] "Oclgrind Simulator (Oclgrind)"                                                       
[7] "GeForce GTX 960 (NVIDIA CUDA)"
```


If the list is empty or the call ends in an error, it is probably because your computer has no OpenCL driver installed. Almost all recent GPUs and CPUs (processors) support OpenCL, so you should be able to run Genexpi.

- For GPUs, try updating your device driver. We also encountered cases where GPUs started being available only after installing a CPU driver (see next bullet).
- For CPUs (the usual processors), you need to install specific drivers. As of 2017-05-22, drivers for Intel CPUs can be downloaded at https://software.intel.com/en-us/articles/opencl-drivers#latest\_CPU\_runtime - (you want “runtime-only”). AMD’s drivers can be found at http://support.amd.com/en-us/kb-articles/Pages/OpenCL2-Driver.aspx.

If the list is non-empty, you can choose which device to use for computation. The best devices to use with Genexpi are (in the following order):

- A dedicated graphics card (GPU) for computing (not connected to a display)
- A Xeon Phi card
- Your processor (CPU)
- A GPU connected to a display

While GPUs will run Genexpi the fastest, using a GPU connected to a display is discouraged as it interferes with the operating system (OS) and may cause your computer to freeze and/or the computations to be reset by the operating system. If you absolutely need to use the GPU running your display, and use Windows, you may want to disable TDR - Timeout detection and recovery (expert only).

With that in mind, let us select a device.


```
#Select the device automatically
deviceSpecs = getDeviceSpecs()
#Select the best GPU device
#deviceSpecs = getDeviceSpecs(deviceType = "gpu")
#Select the best CPU device
#deviceSpecs = getDeviceSpecs(deviceType = "processor")
#Select device by ID reported in listOpenCLDevices()
deviceSpecs = getDeviceSpecs(device = 3)
#Display the selected device
if(is.null(deviceSpecs)) {
  stop("Invalid device")
} else {
  paste0("Using device: ", getJavaDeviceSpecs(deviceSpecs)$getDevice()$toString())
}
```


```
[1] "Using device: gfx804 (AMD Accelerated Parallel Processing)"
```

# Load the sigB data from GSE6865

The original source of data: (https://www.ncbi.nlm.nih.gov/geo/query/acc.cgi?acc=GSE6865)


```
temp <- tempfile();
download.file("ftp://ftp.ncbi.nlm.nih.gov/geo/series/GSE6nnn/GSE6865/matrix/GSE6865_series_matrix.txt.gz", temp)
```


```
trying URL 'ftp://ftp.ncbi.nlm.nih.gov/geo/series/GSE6nnn/GSE6865/matrix/GSE6865_series_matrix.txt.gz'
downloaded 290 KB
```


```
gse6865_raw_df = read.delim(gzfile(temp), comment.char = "!") #Intermediate data frame representation
# Raw profile data. We scale by 1000 to get to more wieldy values
gse6865_raw = as.matrix(gse6865_raw_df[,2:15]) / 1000
rownames(gse6865_raw) = gse6865_raw_df$ID_REF
#Times (in minutes) for the individual samples
gse6865_raw_time = c(0,5,10,15,20,25,30,40,50,60,70,80,90,100)
colnames(gse6865_raw) <- sapply(gse6865_raw_time,  FUN = function(x) { paste0(x,"min")})
#We will compute at 1 minute resolution
smoothTime = 0:100
errorDef = list(relative = 0.2, absolute = 0, minimal = 0.5)
# There are a few genes that have NA values for the first or second measurement
apply(gse6865_raw, MARGIN  = 2, FUN = function(x) { sum(is.na(x)) })
```


```
  0min   5min  10min  15min  20min  25min  30min  40min  50min  60min  70min  80min  90min 100min 
    16     39      0      0      0      0      0      0      0      0      0      0      0      0
```


Above, the number of values that are NA for each time point is shown. Those will be imputed.


```
# For the first measurement, we can expect the value to be 0 (the series are from germination)
gse6865_raw[is.na(gse6865_raw[,1]),1] = 0
# For the second measurement we will go with a linear interpolation
na2 = is.na(gse6865_raw[,2])
gse6865_raw[na2,2] = 0.5 * gse6865_raw[na2,1] + 0.5 * gse6865_raw[na2,3]
# Genes in the regulon according to http://subtiwiki.uni-goettingen.de/wiki/index.php/SigB_regulon as of 2017-02-09
sigBRegulonNames = c("aag", "aldY", "bmr", "bmrR", "bmrU", "bofC", "cdd", "chaA", "clpC", "clpP", "corA", "csbA", "csbB", "csbC", "csbD", "csbX", "ctc", "ctsR", "cypC", "disA", "dps", "era", "gabD", "gsiB", "gspA", "gtaB", "guaD", "hpf", "iolW", "ispD", "ispF", "katE", "katX", "mcsA", "mcsB", "mgsR", "mhqO", "mhqP", "nadE", "nhaX", "ohrB", "opuE", "phoP", "phoR", "plsC", "radA", "rnr", "rpmEB", "rsbRD", "rsbV", "rsbW", "rsbX",  "sodA", "spo0E", "spoVC", "spx", "trxA", "yaaH", "yaaI", "yacL", "ybyB", "ycbP", "ycdF", "ycdG", "yceC", "yceD", "yceE", "yceF", "yceG", "yceH", "ydaD", "ydaE", "ydaF", "ydaG", "ydaJ", "ydaK", "ydaL", "ydaM", "ydaN", "ydaP", "ydaS", "ydaT", "ydbD", "ydeC", "ydhK", "yerD", "yfhD", "yfhE", "yfhF", "yfhK", "yfhL", "yfhM", "yfkD", "yfkH", "yfkI", "yfkJ", "yfkM", "yfkS", "yfkT", "yflA", "yflH", "yflT", "ygxB", "yhcM", "yhdF", "yhdN", "yhxD", "yitT", "yjbC", "yjgB", "yjgC", "yjgD", "yjzE", "ykgA", "ykgB", "ykuT", "ykzI", "ylxP", "ymzB", "yoaA", "yocB", "yocK", "yoxB", "yoxC", "ypuB", "ypuC", "ypuD", "yqhB", "yqhP", "yqhQ", "yqjL", "yraA", "ysdB", "ysnF", "ytaB", "ytkL", "ytxG", "ytxH", "ytxJ", "yugU", "yuzA", "yvaK", "yvgN", "yvgO", "yvrE", "ywiE", "ywjC", "ywlB", "ywmE", "ywmF", "ywsB", "ywtG", "ywzA", "yxaB", "yxbG", "yxiS", "yxjI", "yxjJ", "yxkO", "yxnA", "yxzF", "yycD", "yczO", "ydaC", "yebE", "yebG", "yflD", "yflB", "yisP", "ipi", "yjlB", "ykzN", "spo0E", "yktC", "ykzC", "rbfA", "ytkC", "ytiB", "menC", "menE", "yuzH", "yvbG", "yvzE", "ywsA", "ywjA", "yydC", "yyzG", "yyzH", "yybO")
#cleanup the intermediate results
rm(gse6865_raw_df)
rm(na2)
rm(temp)
```

# Random Profiles

The random profiles are drawn from a Gaussian process with squared exponential kernel and zero mean function. To keep the profile strictly positive, it is transformed via \[f(x) = log(1 + e^x)\]

The source of the random profiles related functions can be found at https://github.com/cas-bioinf/genexpi/blob/master/rpackage/R/randomProfiles.R


```
# Plot 10 random profiles with scale 5 and length 20
plotRandomProfiles(10, gse6865_raw_time, scale = 5, length = 20, main = "Sampled random profiles", ylab="Expression");
```


How do we identify the correct scale and length for the Gaussian process? Lets just find some that look similar enough to the sigB profile. After a bit of experimentation we chose scale = 7 and length = 20 minutes. Below is a sample of how those random trajectories look, compared to the sigB profile from the original data. (this is shown in the paper as Fig. 2)


```
sigBRandomScale = 7
sigBRandomLength = 20
sigBIndex = which(rownames(gse6865_raw) == "sigB")
plotRandomProfiles(10,gse6865_raw_time, scale = sigBRandomScale, length = sigBRandomLength, trueTime = gse6865_raw_time, trueProfile = gse6865_raw[sigBIndex,], main = "Sampled random profiles and the sigB profile (dots)", ylab="expression")
```

# Splining

Splining is done via linear regression of B-spline basis. The source for splining is in the function `splineProfileMatrix` which can be found at https://github.com/cas-bioinf/genexpi/blob/master/rpackage/R/workflow.R The splines are generated using the `bs` function from the `splines` package and the coefficients are found via standard `lm` function. Here is an example:


```
#Generated a noisy measuremets
time = seq(0,6.28, by = 0.2)
testProfile = sin(time) ^ 2 + rnorm(length(time), 0, 0.1)
#Spline with 3 degrees of freedom - not very accurate
splineBasis3 = bs(time, df=3, degree = 3)
splineFit3 = lm(testProfile ~ 0 + splineBasis3)
splinedProfile3 = splineBasis3 %*% splineFit3$coefficients
matplot(time,splinedProfile3, type = "l", main = "Spline 3DF", ylim = c(0,1.1))
points(time, testProfile)
```


```
#Spline with 6 degrees of freedom - nicely removes noise from data
splineBasis6 = bs(time, df=6, degree = 3)
splineFit6 = lm(testProfile ~ 0 + splineBasis6)
splinedProfile6 = splineBasis6 %*% splineFit6$coefficients
matplot(time,splinedProfile6, type = "l", main = "Spline 6DF", ylim = c(0,1.1))
points(time, testProfile)
```

# Testing SigB regulon of Bacilus subtilis

## Genexpi in the sigB regulon

The source for functions used here can be found at https://github.com/cas-bioinf/genexpi/blob/master/rpackage/R/evaluationTools.R

First let us take a sneak peek (just 3 random rounds) when working 6 degrees of freedom for the spline?


```
result6DF = evaluateRandomForRegulon(deviceSpecs, rounds = 3, rawProfiles = gse6865_raw,regulatorName =  "sigB", regulonNames = sigBRegulonNames, time = smoothTime, rawTime = gse6865_raw_time, randomScale = sigBRandomScale, randomLength = sigBRandomLength, errorDef = errorDef, splineDFs = 6)
```


```
paste0("Proportion of true regulations discovered: ",result6DF$trueRatio)
```


```
[1] "Proportion of true regulations discovered: 0.8"
```


```
paste0("Overall proportion of regulations by random profiles: ", result6DF$overallRandomRatio)
```


```
[1] "Overall proportion of regulations by random profiles: 0.393939393939394"
```


Now, let’s test for various splines (we can’t get less DFs than 3, 10 DFs do clearly overfit) and with more rounds.


```
dfsToTest = 3:10
randomRounds = 50
genexpiStart = proc.time();
genexpiOptions(verbose = FALSE) # Hide unnecessary output
variousSplinesResult = testVariousSplines(deviceSpecs, rounds = randomRounds, rawProfiles = gse6865_raw, rawTime = gse6865_raw_time, targetTime = smoothTime, dfsToTest = dfsToTest, regulatorName = "sigB", regulonNames = sigBRegulonNames,randomScale = sigBRandomScale, randomLength = sigBRandomLength, errorDef = errorDef )
genexpiEnd = proc.time();
# Calculate time per round. For each spline (df) variant I have 1 non-random round in addition to the random roudns
genexpiTimePerRound = (genexpiEnd["elapsed"] - genexpiStart["elapsed"]) / (length(dfsToTest) * (randomRounds + 1))
```


```
printVariousSplinesResultsHeader()
```


```
DF  numTested   Regulator       Random      Ratio
```


```
printVariousSplinesResults(variousSplinesResult)
```


```
3   27  0.78    21  0.53    14.3    1.468531    
4   42  0.9 38  0.36    15.06   2.52324 
5   59  0.76    45  0.17    9.8 4.591837    
6   55  0.8 44  0.17    9.24    4.761905    
7   61  0.74    45  0.11    6.92    6.50289 
8   64  0.67    43  0.14    8.76    4.908676    
9   62  0.6 37  0.13    8.18    4.523227    
10  62  0.63    39  0.06    3.66    10.65574
```


```
cat(paste0("Genexpi time per round: ", genexpiTimePerRound, " seconds\n"))
```


```
Genexpi time per round: 26.3183088235294 seconds
```


Let’s plot histogram of the random results for 6DFs:


```
hist(variousSplinesResult[[which(dfsToTest == 6)]]$result$randomRatios, main="", xlab = "Proportion predicted regulated by random profile.")
```


We see that very few random profiles can mimic the behavior of sigB and most are poorly predicted.

## Testing TD-Aracne in the sigB regulon

We tried TD-Aracne in two modes: pairwise and simple. In the simple mode, TD-Aracne is run on the whole regulon at once. This however proved rather slow and didn’t get very good results. So we instead ran TD-Aracne similarly to the way we use Genexpi - a separate run for every TF-Target combination. Since TD-Aracne produce oriented edges, we have two ways to classify the results - either we consider regulation as predicted if the edge has the correct direction (labelled “Direct”), or we are simply interested in presence versus absence of an edge (labelled “Any”). Further we can test the whole regulon or only the genes tested by Genexpi (i.e. remove genes that do not change or can be fit by constant synthesis).

The sources for the functions used in this section and related code can be found at https://github.com/cas-bioinf/genexpi/blob/master/rpackage/R/tdaracneBridge.R


```
#Aracne requires uniform time intervals
aracneSmoothTimeIndices = c(1,11,21,31,41,51,61,71,81,91,101)
aracneRawTimeIndices = c(1,3,5,7:14)
#This was chosen as the minimal value that does not rise warnings
defaultAracneNumBins = 10

randomRoundsAracne = 50
```

# TD-Aracne in simple mode on SigB

For completeness, here is the code to run TD-Aracne in simple mode (on the whole sigB regulon). This takes days to compute! The output has two categories: “Downstream” where every gene that has a directed path from the regulator is treated as regulated and “Connected” where the path need not be directed. Taking only the immediate connections of the regulator had very little performance. (Note that TD-Aracne is solving a much more difficult task here)


```
  #Aracne in simple mode is too slow
  randomRoundsAracneSimple = 20
  dfsToTest_simple = dfsToTest;
  aracneSplinedStart =  proc.time();
  aracneSplinedResults = list()
  for(i in 1:length(dfsToTest_simple)) {
    aracneSplinedResults[[i]] = evaluateTDAracne(
      rounds = randomRoundsAracneSimple, profilesRaw = gse6865_raw, time = smoothTime[aracneSmoothTimeIndices], 
      rawTime = gse6865_raw_time, splineDFs = dfsToTest_simple[i],
      randomScale = sigBRandomScale, randomLength = sigBRandomLength,errorDef =errorDef, regulatorName = "sigB", regulonNames = sigBRegulonNames, numBins = defaultAracneNumBins)
  }
```


```
foreach: simple, scalable parallel programming from Revolution Analytics
Use Revolution R for scalability, fault tolerance and more.
http://www.revolutionanalytics.com
Loading required package: iterators
Loading required package: parallel
```


## Results for simple TD-Aracne, sigB


```
  numCoresUsed = detectCores()[1] - 1; #The script leaves one CPU idle to let you work while it computes
  printTDAracneEvaluationHeader();
```


```
DF  Type    numTested   Regulator       Random      Ratio
```


```
  printTDAracneEvaluation("None", aracneRawResult, variousSplinesResult[[length(variousSplinesResult)]])
```


```
None    Downstream      162 0.86    139 0.43    69.35   2.004326
None    Connected       162 0.91    148 0.55    88.8    1.666667
None    Downstream-L    62  0.87    54  0.43    26.95   2.003711
None    Connected-L     62  0.89    55  0.53    33  1.666667
```


```
  for(i in 1:length(dfsToTest_simple)) {
    printTDAracneEvaluation(dfsToTest_simple[i], aracneSplinedResults[[i]], variousSplinesResult[[i]])
  }
```


```
3   Downstream      162 0.44    72  0.35    56.15   1.28228
3   Connected       162 0.96    156 0.87    140.65  1.109136
3   Downstream-L    27  0.33    9   0.26    7.1 1.267606
3   Connected-L     27  1   27  0.9 24.3    1.111111
4   Downstream      162 0.23    38  0.37    59.45   0.6391926
4   Connected       162 0.93    151 0.75    120.8   1.25
4   Downstream-L    42  0.52    22  0.43    18.25   1.205479
4   Connected-L     42  0.98    41  0.78    32.8    1.25
5   Downstream      162 0.46    74  0.25    40.65   1.820418
5   Connected       162 0.96    155 0.91    147.25  1.052632
5   Downstream-L    59  0.32    19  0.21    12.5    1.52
5   Connected-L     59  1   59  0.95    56.05   1.052632
6   Downstream      162 0.9 145 0.4 64.35   2.253302
6   Connected       162 0.96    155 0.81    131.9   1.175133
6   Downstream-L    55  0.98    54  0.41    22.65   2.384106
6   Connected-L     55  0.98    54  0.83    45.9    1.176471
7   Downstream      162 0.74    120 0.47    75.35   1.592568
7   Connected       162 0.96    156 0.87    140.4   1.111111
7   Downstream-L    61  0.84    51  0.47    28.95   1.761658
7   Connected-L     61  0.97    59  0.87    53.1    1.111111
8   Downstream      162 0.03    5   0.56    90.4    0.05530973
8   Connected       162 0.93    151 0.83    135.2   1.116864
8   Downstream-L    64  0.03    2   0.57    36.6    0.05464481
8   Connected-L     64  0.88    56  0.79    50.4    1.111111
9   Downstream      162 0.88    142 0.39    63.4    2.239748
9   Connected       162 0.96    155 0.62    100.75  1.538462
9   Downstream-L    62  0.92    57  0.43    26.35   2.163188
9   Connected-L     62  0.94    58  0.61    37.7    1.538462
10  Downstream      162 0.02    3   0.24    38.35   0.07822686
10  Connected       162 0.96    155 0.57    93  1.666667
10  Downstream-L    62  0.02    1   0.23    14.25   0.07017544
10  Connected-L     62  0.95    59  0.57    35.4    1.666667
```


```
  cat(paste0("Raw time per round (on ", numCoresUsed," cores): ", aracneRawTimePerRound, " seconds\n"))
```


```
Raw time per round (on 7 cores): 447.014761904763 seconds
```


```
  cat(paste0("Splined time per round (on ", numCoresUsed," cores): ", aracneSplinedTimePerRound, " seconds\n"))
```


```
Splined time per round (on 7 cores): 446.134702380952 seconds
```


```
aracneSplinedPairwiseStart =  proc.time();
aracneSplinedPairwiseResults = list()
for(i in 1:length(dfsToTest)) {
  aracneSplinedPairwiseResults[[i]] = evaluateTDAracnePairwise(
    title = paste("Spline_",dfsToTest[i]), rounds = randomRoundsAracne, profilesRaw = gse6865_raw, 
    time =  smoothTime[aracneSmoothTimeIndices], rawTime = gse6865_raw_time, splineDFs = dfsToTest[i],
    randomScale = sigBRandomScale, randomLength = sigBRandomLength,errorDef =errorDef, regulatorName = "sigB", regulonNames = sigBRegulonNames, numBins = defaultAracneNumBins)
}
aracneSplinedPairwiseEnd = proc.time();
aracneSplinedPairwiseTimePerRound = (aracneSplinedPairwiseEnd["elapsed"] - aracneSplinedPairwiseStart["elapsed"]) / ((randomRoundsAracne + 1) * length(dfsToTest));


aracneRawPairwiseStart =  proc.time();
aracneRawPairwiseResult = evaluateTDAracnePairwise(
  title = "Raw",rounds = randomRoundsAracne, profilesRaw = gse6865_raw[,aracneRawTimeIndices, drop=FALSE], 
  rawTime =  gse6865_raw_time[aracneRawTimeIndices], randomScale = sigBRandomScale, randomLength = sigBRandomLength,
  regulatorName = "sigB", regulonNames = sigBRegulonNames, errorDef =errorDef, numBins = defaultAracneNumBins,
  splineDFs = NULL, time = NULL)
aracneRawPairwiseEnd =  proc.time();
aracneRawPairwiseTimePerRound = (aracneRawPairwiseEnd["elapsed"] - aracneRawPairwiseStart["elapsed"]) / (randomRoundsAracne + 1);

save.image(".RData")

numCoresUsed = detectCores()[1] - 1; #The script leaves one CPU idle to let you work while it computes
```


### Results for pairwise TD-ARACNE, sigB


```
printTDAracneEvaluationHeader();
```


```
DF  Type    numTested   Regulator       Random      Ratio
```


```
#Run on raw data is compared against the highest definition spline
printTDAracnePairwiseEvaluation("None", aracneRawPairwiseResult, variousSplinesResult[[length(variousSplinesResult)]])
```


```
None    Direct      161 0.19    31  0.1 15.54   1.994852
None    Any         161 0.3 49  0.24    38.88   1.260288
None    Direct-L    62  0.29    18  0.09    5.62    3.202847
None    Any-L       62  0.31    19  0.25    15.64   1.214834
```


```
for(i in 1:length(dfsToTest)) {
  printTDAracnePairwiseEvaluation(dfsToTest[i], aracneSplinedPairwiseResults[[i]], variousSplinesResult[[i]])
}
```


```
3   Direct      161 0.11    18  0.23    37.62   0.4784689
3   Any         161 0.32    52  0.57    91.84   0.5662021
3   Direct-L    27  0.11    3   0.21    5.68    0.528169
3   Any-L       27  0.26    7   0.65    17.48   0.4004577
4   Direct      161 0.08    13  0.12    19.16   0.6784969
4   Any         161 0.22    36  0.34    54.82   0.6566946
4   Direct-L    42  0.21    9   0.19    7.96    1.130653
4   Any-L       42  0.31    13  0.36    15.04   0.8643617
5   Direct      161 0.17    27  0.15    24.64   1.095779
5   Any         161 0.24    39  0.41    65.4    0.5963303
5   Direct-L    59  0.08    5   0.14    8.52    0.5868545
5   Any-L       59  0.25    15  0.47    27.66   0.5422993
6   Direct      161 0.33    53  0.12    19.62   2.701325
6   Any         161 0.4 65  0.33    53.04   1.22549
6   Direct-L    55  0.44    24  0.12    6.84    3.508772
6   Any-L       55  0.53    29  0.4 21.96   1.320583
7   Direct      161 0.2 32  0.13    20.52   1.559454
7   Any         161 0.3 48  0.3 48.42   0.9913259
7   Direct-L    61  0.26    16  0.12    7.48    2.139037
7   Any-L       61  0.28    17  0.35    21.22   0.801131
8   Direct      161 0.1 16  0.12    18.84   0.8492569
8   Any         161 0.29    46  0.3 48.12   0.9559435
8   Direct-L    64  0.09    6   0.1 6.46    0.9287926
8   Any-L       64  0.19    12  0.28    17.98   0.6674082
9   Direct      161 0.19    30  0.12    19.02   1.577287
9   Any         161 0.24    39  0.28    44.42   0.8779829
9   Direct-L    62  0.27    17  0.13    8.08    2.10396
9   Any-L       62  0.31    19  0.26    16.38   1.159951
10  Direct      161 0.03    5   0.12    19.14   0.261233
10  Any         161 0.12    20  0.24    38.96   0.513347
10  Direct-L    62  0.05    3   0.13    7.76    0.3865979
10  Any-L       62  0.08    5   0.25    15.24   0.328084
```


```
cat(paste0("Raw time per round (on ", numCoresUsed," cores): ", aracneRawPairwiseTimePerRound, " seconds\n"))
```


```
Raw time per round (on 7 cores): 36.9860784313724 seconds
```


```
cat(paste0("Splined time per round (on ", numCoresUsed," cores): ", aracneSplinedPairwiseTimePerRound , " seconds\n"))
```


```
Splined time per round (on 7 cores): 39.4223039215686 seconds
```

# The SigR regulon of S. coelicolor

This is basically the same as previously, just with different data.


```
#The data are included in the package
data(gse44415_raw)
gse44415_raw_time = as.numeric(colnames(gse44415_raw))
smoothTime_r = -30:330

sigRSco = "5216"
sigRRegulon_Kallifidas = c("0564", "0569", "0570", "0882", "0885", "1340", "1384", "1392", "1421", "1513", "1831", "1839", "1869", "1919", "1920", "1958", "1995", "1996", "1997", "2161", "2537", "2618", "2619", "2634", "2643", "2849", "2910", "2911", "2912", "3083", "3091", "3162", "3187", "3202", "3206", "3373", "3403", "3415", "3764", "3889", "3890", "4039", "4198", "4199", "4204", "4297", "4316", "4501", "4736", "4770", "4956", "4967", "4968", "5042", "5163", "5177", "5178", "5187", "5217", "5285", "5359", "5465", "5545", "5655", "5754", "5755", "5796", "6061", "6423", "6424", "6551", "6577", "6759", "7117", "7203", "7632");


sigRRegulon_Kim = c("0569", "0570", "0882", "0884", "0885", "0917", "0973", "1084", "1085", "1142", "1238", "1425", "1426", "1513", "1598", "1599", "1600", "1618", "1619", "1643", "1644", "1645", "1646", "1647", "1648", "1758", "1869", "1919", "1920", "1936", "1937", "1938", "1939", "1958", "1995", "1996", "1997", "2124", "2154", "2161", "2162", "2194", "2254", "2310", "2331", "2481", "2537", "2538", "2539", "2595", "2617", "2618", "2619", "2634", "2635", "2642", "2643", "2763", "2816", "2849", "2910", "2911", "s02", "3083", "3091", "3162", "3187", "3206", "3207", "3295", "3296", "3373", "3403", "3442", "3449", "3450", "3451", "3509", "3764", "3765", "3766", "3767", "3889", "3890", "4039", "4040", "4109", "4203", "4204", "4205", "4297", "4298", "4299", "4418", "4419", "4420", "4561", "4770", "4771", "4797", "4833", "4834", "4835", "4956", "4966", "4967", "4968", "5042", "5065", "5163", "5178", "5187", "5188", "5217", "5284", "5285", "5357", "5359", "5360", "5361", "5465", "5490", "5545", "5552", "5705", "5706", "5707", "5708", "5709", "5754", "5796", "5820", "5864", "5865", "6061", "6126", "6127", "6423", "6551", "6759", "6760", "6761", "6762", "6763", "6764", "6765", "6766", "6767", "6768", "6769", "6770", "6771", "6775", "6776", "7140", "7631", "7632", "7784", "7785")

sigRRegulon_Kim_Strong = c("0570", "0882", "0884", "0885", "0917", "0973", "1084", "1085", "1142", "1238", "1425", "1426", "1513", "1600", "1619", "1648", "1758", "1869", "1920", "1936", "1958", "1995", "1997", "2124", "2154", "2161", "2162", "2194", "2254", "2310", "2331", "2481", "2537", "2595", "2619", "2634", "2635", "2642", "2643", "2763", "2816", "2849", "2911", "s02", "3083", "3091", "3162", "3187", "3206", "3207", "3296", "3373", "3403", "3442", "3450", "3451", "3509", "3765", "3766", "3767", "3890", "4039", "4040", "4109", "4203", "4204", "4297", "4419", "4420", "4561", "4770", "4797", "4835", "4956", "4966", "4967", "5042", "5065", "5163", "5178", "5187", "5188", "5284", "5285", "5357", "5359", "5465", "5490", "5545", "5552", "5705", "5754", "5796", "5820", "5864", "6061", "6126", "6127", "6423", "6551", "6759", "6775", "6776", "7140", "7631", "7632", "7784")

#Filter out the genes I do not have in data:
sigRRegulon_Kallifidas = sigRRegulon_Kallifidas[sigRRegulon_Kallifidas %in% rownames(gse44415_raw)]
sigRRegulon_Kim = sigRRegulon_Kim[sigRRegulon_Kim %in% rownames(gse44415_raw)]
sigRRegulon_Kim_Strong = sigRRegulon_Kim_Strong[sigRRegulon_Kim_Strong %in% rownames(gse44415_raw)]
```


Once again, we try to find a setting for the profile generator to roughly match the sigR profile.


```
sigRRandomScale = 3
sigRRandomLength = 30
sigRIndex = which(rownames(gse44415_raw) == sigRSco)
plotRandomProfiles(10,gse44415_raw_time, sigRRandomScale, sigRRandomLength, trueTime = gse44415_raw_time, trueProfile = gse44415_raw[sigRIndex,], main = "Sampled random profiles and the sigR profile (dots)", ylab="expression")
```


## Genexpi and SigR regulon


```
dfsToTest_r = 3:11
genexpiStart_r = proc.time();
genexpiOptions(verbose = FALSE) # Hide unnecessary output
variousSplinesResult_r = testVariousSplines(deviceSpecs, rounds = randomRounds, rawProfiles = gse44415_raw[rownames(gse44415_raw) %in% c(sigRSco, sigRRegulon_Kallifidas),], rawTime = gse44415_raw_time, targetTime = smoothTime_r, dfsToTest = dfsToTest_r, regulatorName = sigRSco, regulonNames = sigRRegulon_Kallifidas,randomScale = sigRRandomScale, randomLength = sigRRandomLength, errorDef = errorDef )
genexpiEnd_r = proc.time();
# Calculate time per round. For each spline (df) variant I have 1 non-random round in addition to the random roudns
genexpiTimePerRound_r = (genexpiEnd_r["elapsed"] - genexpiStart_r["elapsed"]) / (length(dfsToTest) * (randomRounds + 1))
save.image(".RData")
```


### Results for Genexpi and the SigR regulon (Kallifidas et al.)


```
printVariousSplinesResultsHeader()
```


```
DF  numTested   Regulator       Random      Ratio
```


```
printVariousSplinesResults(variousSplinesResult_r)
```


```
3   43  0.93    40  0.04    1.72    23.25581    
4   50  0.78    39  0.01    0.68    57.35294    
5   51  0.37    19  0.01    0.5 38  
6   51  0.29    15  0.01    0.42    35.71429    
7   54  0.22    12  0.01    0.64    18.75   
8   53  0.21    11  0.01    0.38    28.94737    
9   52  0.17    9   0.01    0.48    18.75   
10  55  0.18    10  0.01    0.42    23.80952    
11  56  0.21    12  0.01    0.3 40
```


```
cat(paste0("Genexpi time per round: ", genexpiTimePerRound_r, " seconds\n"))
```


```
Genexpi time per round: 108.876666666667 seconds
```


```
genexpiStart_r_kim = proc.time();
genexpiOptions(verbose = FALSE) # Hide unnecessary output
variousSplinesResult_r_kim = testVariousSplines(deviceSpecs, rounds = randomRounds, rawProfiles = gse44415_raw[rownames(gse44415_raw) %in% c(sigRSco, sigRRegulon_Kim),], rawTime = gse44415_raw_time, targetTime = smoothTime_r, dfsToTest = dfsToTest_r, regulatorName = sigRSco, regulonNames = sigRRegulon_Kim,randomScale = sigRRandomScale, randomLength = sigRRandomLength, errorDef = errorDef )
genexpiEnd_r_kim = proc.time();
# Calculate time per round. For each spline (df) variant I have 1 non-random round in addition to the random roudns
genexpiTimePerRound_r_kim = (genexpiEnd_r_kim["elapsed"] - genexpiStart_r_kim["elapsed"]) / (length(dfsToTest) * (randomRounds + 1))
```

### Results for the whole sigR regulon of Kim et al.


```
printVariousSplinesResultsHeader()
```


```
DF  numTested   Regulator       Random      Ratio
```


```
printVariousSplinesResults(variousSplinesResult_r_kim)
```


```
3   67  0.91    61  0.07    5   12.2    
4   80  0.81    65  0.03    2.54    25.59055    
5   82  0.44    36  0.03    2.44    14.7541 
6   82  0.35    29  0.03    2.06    14.07767    
7   89  0.31    28  0.03    2.48    11.29032    
8   90  0.22    20  0.02    2.16    9.259259    
9   93  0.25    23  0.02    2.04    11.27451    
10  99  0.24    24  0.01    1.18    20.33898    
11  97  0.24    23  0.01    1   23
```


```
cat(paste0("Genexpi time per round: ", genexpiTimePerRound_r_kim, " seconds\n"))
```


```
Genexpi time per round: 150.260147058824 seconds
```


```
genexpiStart_r_kim_strong = proc.time();
genexpiOptions(verbose = FALSE) # Hide unnecessary output
variousSplinesResult_r_kim_strong = testVariousSplines(deviceSpecs, rounds = randomRounds, rawProfiles = gse44415_raw[rownames(gse44415_raw) %in% c(sigRSco, sigRRegulon_Kim_Strong),], rawTime = gse44415_raw_time, targetTime = smoothTime_r, dfsToTest = dfsToTest_r, regulatorName = sigRSco, regulonNames = sigRRegulon_Kim_Strong, randomScale = sigRRandomScale, randomLength = sigRRandomLength, errorDef = errorDef )
```

### Results for the ‘STRONG’ subpart of sigR regulon of Kim et al.


```
printVariousSplinesResultsHeader()
```


```
DF  numTested   Regulator       Random      Ratio
```


```
printVariousSplinesResults(variousSplinesResult_r_kim_strong)
```


```
3   58  0.9 52  0.05    3.18    16.3522 
4   69  0.78    54  0.03    2.28    23.68421    
5   71  0.46    33  0.05    3.44    9.593023    
6   70  0.33    23  0.02    1.54    14.93506    
7   74  0.27    20  0.03    2.14    9.345794    
8   75  0.19    14  0.03    1.96    7.142857    
9   78  0.22    17  0.02    1.78    9.550562    
10  83  0.2 17  0.01    1   17  
11  82  0.21    17  0.01    0.88    19.31818
```


```
cat(paste0("Genexpi time per round: ", genexpiTimePerRound_r_kim_strong, " seconds\n"))
```


```
Genexpi time per round: 131.387205882353 seconds
```

### Simple TD-ARACNE on SigR, Kallifidas et al.


```
  dfsToTest_simple_r = dfsToTest_r;
  aracneSplinedStart_r =  proc.time();
  aracneSplinedResults_r = list()
  for(i in 1:length(dfsToTest_simple_r)) {
    aracneSplinedResults_r[[i]] = evaluateTDAracne(
      rounds = randomRoundsAracneSimple, profilesRaw = gse44415_raw, time = gse44415_raw_time, 
      rawTime = gse44415_raw_time, splineDFs = dfsToTest_simple_r[i],
      randomScale = sigRRandomScale, randomLength = sigRRandomLength,errorDef =errorDef, regulatorName = sigRSco, regulonNames = sigRRegulon_Kallifidas, numBins = defaultAracneNumBins)
  }
  aracneSplinedEnd_r = proc.time();
  aracneSplinedTimePerRound_r = (aracneSplinedEnd_r["elapsed"] - aracneSplinedStart_r["elapsed"]) / ((randomRoundsAracneSimple + 1) * length(dfsToTest_simple_r));
  
  aracneRawStart_r =  proc.time();
  aracneRawResult_r = evaluateTDAracne(
      rounds = randomRoundsAracneSimple, profilesRaw = gse44415_raw, 
      time = NULL, 
      rawTime = gse44415_raw_time, splineDFs = NULL,
      randomScale = sigRRandomScale, randomLength = sigRRandomLength,errorDef =errorDef, regulatorName = sigRSco, regulonNames = sigRRegulon_Kallifidas, numBins = defaultAracneNumBins)
    

  aracneRawEnd_r =  proc.time();
  aracneRawTimePerRound_r = (aracneRawEnd_r["elapsed"] - aracneRawStart_r["elapsed"]) / (randomRoundsAracneSimple + 1);
```

## Results for simple TD-Aracne, sigR


```
  numCoresUsed = detectCores()[1] - 1; #The script leaves one CPU idle to let you work while it computes
  printTDAracneEvaluationHeader();
```


```
DF  Type    numTested   Regulator       Random      Ratio
```


```
  printTDAracneEvaluation("None", aracneRawResult_r, variousSplinesResult_r[[length(variousSplinesResult_r)]])
```


```
None    Downstream      71  0.94    67  0.76    54.2    1.236162
None    Connected       71  0.96    68  0.91    64.6    1.052632
None    Downstream-L    57  0.96    55  0.78    44.4    1.238739
None    Connected-L     57  0.96    55  0.92    52.25   1.052632
```


```
  for(i in 1:length(dfsToTest_simple_r)) {
    printTDAracneEvaluation(dfsToTest_simple_r[i], aracneSplinedResults_r[[i]], variousSplinesResult_r[[i]])
  }
```


```
3   Downstream      71  0.42    30  0.5 35.15   0.8534851
3   Connected       71  0.99    70  0.99    70  1
3   Downstream-L    43  0.4 17  0.46    19.65   0.8651399
3   Connected-L     43  1   43  1   43  1
4   Downstream      71  0.01    1   0.62    44.25   0.02259887
4   Connected       71  0.99    70  0.99    70  1
4   Downstream-L    50  0.02    1   0.68    34  0.02941176
4   Connected-L     50  1   50  1   50  1
5   Downstream      71  0.8 57  0.38    26.9    2.118959
5   Connected       71  0.99    70  0.99    70  1
5   Downstream-L    51  0.88    45  0.41    21  2.142857
5   Connected-L     51  1   51  1   51  1
6   Downstream      71  0.89    63  0.57    40.3    1.563275
6   Connected       71  0.99    70  0.99    70  1
6   Downstream-L    51  0.96    49  0.6 30.6    1.601307
6   Connected-L     51  1   51  1   51  1
7   Downstream      71  0.76    54  0.59    41.8    1.291866
7   Connected       71  0.99    70  0.89    63  1.111111
7   Downstream-L    54  0.83    45  0.62    33.75   1.333333
7   Connected-L     54  1   54  0.9 48.6    1.111111
8   Downstream      71  0.93    66  0.47    33.4    1.976048
8   Connected       71  0.99    70  0.99    70  1
8   Downstream-L    53  1   53  0.5 26.6    1.992481
8   Connected-L     53  1   53  1   53  1
9   Downstream      71  0.86    61  0.51    36.55   1.668947
9   Connected       71  0.99    70  0.84    59.5    1.176471
9   Downstream-L    52  0.96    50  0.56    29.15   1.715266
9   Connected-L     52  1   52  0.85    44.2    1.176471
10  Downstream      71  0.01    1   0.61    43.3    0.02309469
10  Connected       71  0.99    70  0.79    56  1.25
10  Downstream-L    55  0.02    1   0.63    34.55   0.02894356
10  Connected-L     55  1   55  0.8 44  1.25
11  Downstream      71  0   0   0.68    48.3    0
11  Connected       71  0.99    70  0.94    66.5    1.052632
11  Downstream-L    57  0   0   0.7 39.9    0
11  Connected-L     57  1   57  0.95    54.15   1.052632
```


```
  cat(paste0("Raw time per round (on ", numCoresUsed," cores): ", aracneRawTimePerRound_r , " seconds\n"))
```


```
Raw time per round (on 7 cores): 181.435238095239 seconds
```


```
  cat(paste0("Splined time per round (on ", numCoresUsed," cores): ", aracneSplinedTimePerRound_r, " seconds\n"))
```


```
Splined time per round (on 7 cores): 183.573333333333 seconds
```


### Simple TD-ARACNE on SigR, Kim et al.


```
  aracneSplinedStart_r_kim =  proc.time();
  aracneSplinedResults_r_kim = list()
  for(i in 1:length(dfsToTest_simple_r)) {
    aracneSplinedResults_r_kim[[i]] = evaluateTDAracne(
      rounds = randomRoundsAracneSimple, profilesRaw = gse44415_raw, time = gse44415_raw_time, 
      rawTime = gse44415_raw_time, splineDFs = dfsToTest_simple_r[i],
      randomScale = sigRRandomScale, randomLength = sigRRandomLength,errorDef =errorDef, regulatorName = sigRSco, regulonNames = sigRRegulon_Kim, numBins = defaultAracneNumBins)
  }
  aracneSplinedEnd_r_kim = proc.time();
  aracneSplinedTimePerRound_r_kim = (aracneSplinedEnd_r_kim["elapsed"] - aracneSplinedStart_r_kim["elapsed"]) / ((randomRoundsAracneSimple + 1) * length(dfsToTest_simple_r));
  
  aracneRawStart_r_kim =  proc.time();
  aracneRawResult_r_kim = evaluateTDAracne(
      rounds = randomRoundsAracneSimple, profilesRaw = gse44415_raw, 
      time = NULL, 
      rawTime = gse44415_raw_time, splineDFs = NULL,
      randomScale = sigRRandomScale, randomLength = sigRRandomLength,errorDef =errorDef, regulatorName = sigRSco, regulonNames = sigRRegulon_Kim, numBins = defaultAracneNumBins)
    

  aracneRawEnd_r_kim =  proc.time();
  aracneRawTimePerRound_r_kim = (aracneRawEnd_r["elapsed"] - aracneRawStart_r["elapsed"]) / (randomRoundsAracneSimple + 1);
```

## Results for simple TD-Aracne, sigR Kim et al.


```
  numCoresUsed = detectCores()[1] - 1; #The script leaves one CPU idle to let you work while it computes
  printTDAracneEvaluationHeader();
```


```
DF  Type    numTested   Regulator       Random      Ratio
```


```
  printTDAracneEvaluation("None", aracneRawResult_r_kim, variousSplinesResult_r_kim[[length(variousSplinesResult_r_kim)]])
```


```
None    Downstream      146 0.95    139 0.77    112.25  1.238307
None    Connected       146 0.96    140 0.91    133 1.052632
None    Downstream-L    97  0.97    94  0.78    75.55   1.244209
None    Connected-L     97  0.98    95  0.93    90.25   1.052632
```


```
  for(i in 1:length(dfsToTest_simple_r)) {
    printTDAracneEvaluation(dfsToTest_simple_r[i], aracneSplinedResults_r_kim[[i]], variousSplinesResult_r_kim[[i]])
  }
```


```
3   Downstream      146 0.66    97  0.67    98.15   0.9882832
3   Connected       146 0.99    145 0.99    145 1
3   Downstream-L    67  0.58    39  0.6 40.15   0.9713574
3   Connected-L     67  1   67  1   67  1
4   Downstream      146 0   0   0.67    97.95   0
4   Connected       146 0.98    143 0.98    143 1
4   Downstream-L    80  0   0   0.73    58.55   0
4   Connected-L     80  1   80  1   80  1
5   Downstream      146 0.84    123 0.43    63.2    1.946203
5   Connected       146 0.99    144 0.99    144 1
5   Downstream-L    82  0.93    76  0.47    38.5    1.974026
5   Connected-L     82  1   82  1   82  1
6   Downstream      146 0.01    2   0.34    50.1    0.03992016
6   Connected       146 0.98    143 0.98    142.7   1.002102
6   Downstream-L    82  0   0   0.35    29  0
6   Connected-L     82  1   82  1   82  1
7   Downstream      146 0.03    4   0.61    88.4    0.04524887
7   Connected       146 0.97    142 0.97    142.05  0.999648
7   Downstream-L    89  0.04    4   0.63    56.05   0.07136485
7   Connected-L     89  1   89  1   88.9    1.001125
8   Downstream      146 0.95    138 0.65    94.75   1.456464
8   Connected       146 0.99    144 0.94    136.8   1.052632
8   Downstream-L    90  0.99    89  0.69    61.7    1.442464
8   Connected-L     90  1   90  0.95    85.5    1.052632
9   Downstream      146 0.03    4   0.72    105 0.03809524
9   Connected       146 0.99    144 0.89    129.6   1.111111
9   Downstream-L    93  0.03    3   0.72    66.5    0.04511278
9   Connected-L     93  1   93  0.9 83.7    1.111111
10  Downstream      146 0.01    2   0.77    113.05  0.01769129
10  Connected       146 0.99    145 0.94    137.75  1.052632
10  Downstream-L    99  0.02    2   0.79    77.95   0.02565747
10  Connected-L     99  1   99  0.95    94.05   1.052632
11  Downstream      146 0.14    20  0.68    98.8    0.2024291
11  Connected       146 0.99    145 0.94    137.75  1.052632
11  Downstream-L    97  0.11    11  0.67    65.45   0.1680672
11  Connected-L     97  1   97  0.95    92.15   1.052632
```


```
  cat(paste0("Raw time per round (on ", numCoresUsed," cores): ", aracneRawTimePerRound_r_kim, " seconds\n"))
```


```
Raw time per round (on 7 cores): 181.435238095239 seconds
```


```
  cat(paste0("Splined time per round (on ", numCoresUsed," cores): ", aracneSplinedTimePerRound_r_kim, " seconds\n"))
```


```
Splined time per round (on 7 cores): 446.204708994709 seconds
```

## TD-Aracne Pairwise and sigRRegulon


```
aracneSplinedPairwiseStart_r =  proc.time();
aracneSplinedPairwiseResults_r = list()

for(i in 1:length(dfsToTest_r)) {
  aracneSplinedPairwiseResults_r[[i]] = evaluateTDAracnePairwise(
    title = paste("Spline_",dfsToTest_r[i]), rounds = randomRoundsAracne, 
    profilesRaw = gse44415_raw[rownames(gse44415_raw) %in% c(sigRSco, sigRRegulon_Kallifidas),], 
    time =  gse44415_raw_time, rawTime = gse44415_raw_time, splineDFs = dfsToTest_r[i],
    randomScale = sigRRandomScale, randomLength = sigRRandomLength,errorDef =errorDef, regulatorName = sigRSco, regulonNames = sigRRegulon_Kallifidas, numBins = defaultAracneNumBins)
}
aracneSplinedPairwiseEnd_r = proc.time();
aracneSplinedPairwiseTimePerRound_r = (aracneSplinedPairwiseEnd_r["elapsed"] - aracneSplinedPairwiseStart_r["elapsed"]) / ((randomRoundsAracne + 1) * length(dfsToTest_r));


aracneRawPairwiseStart_r =  proc.time();
aracneRawPairwiseResult_r = evaluateTDAracnePairwise(
  title = "Raw",rounds = randomRoundsAracne, 
  profilesRaw = gse44415_raw[rownames(gse44415_raw) %in% c(sigRSco, sigRRegulon_Kallifidas),], 
  rawTime =  gse44415_raw_time, randomScale = sigRRandomScale, randomLength = sigRRandomLength,
  regulatorName = sigRSco, regulonNames = sigRRegulon_Kallifidas, errorDef = errorDef, numBins = defaultAracneNumBins,
  splineDFs = NULL, time = NULL)
aracneRawPairwiseEnd_r =  proc.time();
aracneRawPairwiseTimePerRound_r = (aracneRawPairwiseEnd_r["elapsed"] - aracneRawPairwiseStart_r["elapsed"]) / (randomRoundsAracne + 1);


numCoresUsed = detectCores()[1] - 1; #The script leaves one CPU idle to let you work while it computes
```


### Results for pairwise TD-ARACNE on SigR Regulon of Kallifidas et al.


```
printTDAracneEvaluationHeader();
```


```
DF  Type    numTested   Regulator       Random      Ratio
```


```
#Run on raw data is compared against the highest definition spline
printTDAracnePairwiseEvaluation("None", aracneRawPairwiseResult_r, variousSplinesResult_r[[length(variousSplinesResult_r)]])
```


```
None    Direct      70  0.2 14  0.17    11.58   1.208981
None    Any         70  0.41    29  0.32    22.48   1.290036
None    Direct-L    56  0.21    12  0.17    9.4 1.276596
None    Any-L       56  0.43    24  0.33    18.66   1.286174
```


```
for(i in 1:length(dfsToTest_r)) {
  printTDAracnePairwiseEvaluation(dfsToTest_r[i], aracneSplinedPairwiseResults_r[[i]], variousSplinesResult_r[[i]])
}
```


```
3   Direct      70  0.03    2   0.29    20.26   0.09871668
3   Any         70  0.47    33  0.38    26.38   1.250948
3   Direct-L    43  0   0   0.34    14.82   0
3   Any-L       43  0.63    27  0.43    18.56   1.454741
4   Direct      70  0.04    3   0.21    14.78   0.202977
4   Any         70  0.33    23  0.35    24.46   0.9403107
4   Direct-L    50  0.06    3   0.23    11.34   0.2645503
4   Any-L       50  0.18    9   0.35    17.54   0.5131129
5   Direct      70  0.21    15  0.1 6.74    2.225519
5   Any         70  0.27    19  0.27    18.74   1.013874
5   Direct-L    51  0.22    11  0.09    4.48    2.455357
5   Any-L       51  0.27    14  0.26    13.48   1.038576
6   Direct      70  0.16    11  0.15    10.38   1.05973
6   Any         70  0.36    25  0.31    21.42   1.167134
6   Direct-L    51  0.16    8   0.15    7.82    1.023018
6   Any-L       51  0.31    16  0.33    16.94   0.94451
7   Direct      70  0.23    16  0.18    12.86   1.244168
7   Any         70  0.36    25  0.31    21.46   1.164958
7   Direct-L    54  0.28    15  0.19    10.12   1.482213
7   Any-L       54  0.39    21  0.32    17.32   1.212471
8   Direct      70  0.26    18  0.21    14.78   1.217862
8   Any         70  0.37    26  0.32    22.66   1.147396
8   Direct-L    53  0.32    17  0.22    11.82   1.43824
8   Any-L       53  0.43    23  0.34    18.2    1.263736
9   Direct      70  0.21    15  0.17    12.24   1.22549
9   Any         70  0.46    32  0.32    22.14   1.445348
9   Direct-L    52  0.23    12  0.17    8.96    1.339286
9   Any-L       52  0.46    24  0.33    16.94   1.416765
10  Direct      70  0.16    11  0.21    14.68   0.7493188
10  Any         70  0.43    30  0.35    24.72   1.213592
10  Direct-L    55  0.18    10  0.21    11.68   0.8561644
10  Any-L       55  0.45    25  0.37    20.48   1.220703
11  Direct      70  0.2 14  0.2 14.28   0.9803922
11  Any         70  0.41    29  0.41    28.92   1.002766
11  Direct-L    56  0.25    14  0.21    11.62   1.204819
11  Any-L       56  0.48    27  0.42    23.56   1.14601
```


```
cat(paste0("Raw time per round (on ", numCoresUsed," cores): ", aracneRawPairwiseTimePerRound_r, " seconds\n"))
```


```
Raw time per round (on 7 cores): 19.1333333333334 seconds
```


```
cat(paste0("Splined time per round (on ", numCoresUsed," cores): ", aracneSplinedPairwiseTimePerRound_r , " seconds\n"))
```


```
Splined time per round (on 7 cores): 21.4573638344227 seconds
```


```
aracneSplinedPairwiseStart_r_Kim =  proc.time();
aracneSplinedPairwiseResults_r_Kim = list()

for(i in 1:length(dfsToTest_r)) {
  aracneSplinedPairwiseResults_r_Kim[[i]] = evaluateTDAracnePairwise(
    title = paste("Spline_",dfsToTest_r[i]), rounds = randomRoundsAracne, 
    profilesRaw = gse44415_raw[rownames(gse44415_raw) %in% c(sigRSco, sigRRegulon_Kim),], 
    time =  gse44415_raw_time, rawTime = gse44415_raw_time, splineDFs = dfsToTest_r[i],
    randomScale = sigRRandomScale, randomLength = sigRRandomLength,errorDef =errorDef, regulatorName = sigRSco, regulonNames = sigRRegulon_Kim, numBins = defaultAracneNumBins)
}
aracneSplinedPairwiseEnd_r_Kim = proc.time();
aracneSplinedPairwiseTimePerRound_r_Kim = (aracneSplinedPairwiseEnd_r_Kim["elapsed"] - aracneSplinedPairwiseStart_r_Kim["elapsed"]) / ((randomRoundsAracne + 1) * length(dfsToTest_r));


aracneRawPairwiseStart_r_Kim =  proc.time();
aracneRawPairwiseResult_r_Kim = evaluateTDAracnePairwise(
  title = "Raw",rounds = randomRoundsAracne, 
  profilesRaw = gse44415_raw[rownames(gse44415_raw) %in% c(sigRSco, sigRRegulon_Kim),], 
  rawTime =  gse44415_raw_time, randomScale = sigRRandomScale, randomLength = sigRRandomLength,
  regulatorName = sigRSco, regulonNames = sigRRegulon_Kim, errorDef = errorDef, numBins = defaultAracneNumBins,
  splineDFs = NULL, time = NULL)
aracneRawPairwiseEnd_r_Kim =  proc.time();
aracneRawPairwiseTimePerRound_r_Kim = (aracneRawPairwiseEnd_r_Kim["elapsed"] - aracneRawPairwiseStart_r_Kim["elapsed"]) / (randomRoundsAracne + 1);


numCoresUsed = detectCores()[1] - 1; #The script leaves one CPU idle to let you work while it computes
```

### Results for pairwise TD-ARACNE - Kim et al.


```
#Run on raw data is compared against the highest definition spline
printTDAracneEvaluationHeader();
```


```
DF  Type    numTested   Regulator       Random      Ratio
```


```
printTDAracnePairwiseEvaluation("None", aracneRawPairwiseResult_r_Kim, variousSplinesResult_r_kim[[length(variousSplinesResult_r_kim)]])
```


```
None    Direct      145 0.15    22  0.19    27.54   0.7988381
None    Any         145 0.37    53  0.35    50.5    1.049505
None    Direct-L    144 0.15    21  0.19    27.38   0.7669832
None    Any-L       144 0.36    52  0.35    50.32   1.033386
```


```
for(i in 1:length(dfsToTest_r)) {
  printTDAracnePairwiseEvaluation(dfsToTest_r[i], aracneSplinedPairwiseResults_r_Kim[[i]], variousSplinesResult_r_kim[[i]])
}
```


```
3   Direct      145 0.06    9   0.24    34.52   0.2607184
3   Any         145 0.43    63  0.33    48.52   1.298434
3   Direct-L    142 0.06    8   0.24    33.8    0.2366864
3   Any-L       142 0.44    62  0.34    47.6    1.302521
4   Direct      145 0.02    3   0.13    18.66   0.1607717
4   Any         145 0.3 44  0.34    49.56   0.8878128
4   Direct-L    142 0.02    3   0.13    18.36   0.1633987
4   Any-L       142 0.31    44  0.34    48.82   0.90127
5   Direct      145 0.12    17  0.1 14.96   1.136364
5   Any         145 0.23    33  0.3 42.94   0.7685142
5   Direct-L    142 0.12    17  0.1 14.58   1.165981
5   Any-L       142 0.23    33  0.3 42.18   0.7823613
6   Direct      145 0.08    12  0.13    18.18   0.660066
6   Any         145 0.36    52  0.28    41.2    1.262136
6   Direct-L    143 0.08    12  0.13    18.04   0.6651885
6   Any-L       143 0.36    51  0.28    40.44   1.261128
7   Direct      145 0.19    27  0.13    19.18   1.407716
7   Any         145 0.35    51  0.27    38.74   1.316469
7   Direct-L    143 0.18    26  0.13    18.92   1.374207
7   Any-L       143 0.35    50  0.27    38.16   1.310273
8   Direct      145 0.23    33  0.11    15.9    2.075472
8   Any         145 0.39    57  0.23    32.86   1.734632
8   Direct-L    144 0.23    33  0.11    15.76   2.093909
8   Any-L       144 0.4 57  0.23    32.62   1.747394
9   Direct      145 0.19    27  0.15    21.84   1.236264
9   Any         145 0.41    59  0.3 43.38   1.360074
9   Direct-L    144 0.19    27  0.15    21.68   1.245387
9   Any-L       144 0.4 58  0.3 42.94   1.350722
10  Direct      145 0.17    25  0.19    27.28   0.9164223
10  Any         145 0.42    61  0.36    51.62   1.181713
10  Direct-L    144 0.17    25  0.19    26.98   0.9266123
10  Any-L       144 0.42    60  0.35    51.08   1.174628
11  Direct      145 0.18    26  0.23    33.32   0.7803121
11  Any         145 0.35    51  0.38    54.68   0.9326993
11  Direct-L    144 0.18    26  0.23    33.06   0.7864489
11  Any-L       144 0.35    51  0.38    54.28   0.9395726
```


```
cat(paste0("Raw time per round (on ", numCoresUsed," cores): ", aracneRawPairwiseTimePerRound_r_Kim, " seconds\n"))
```


```
Raw time per round (on 7 cores): 43.72568627451 seconds
```


```
cat(paste0("Splined time per round (on ", numCoresUsed," cores): ", aracneSplinedPairwiseTimePerRound_r_Kim , " seconds\n"))
```


```
Splined time per round (on 7 cores): 44.4295424836601 seconds
```


```
aracneSplinedPairwiseStart_r_Kim_Strong =  proc.time();
aracneSplinedPairwiseResults_r_Kim_Strong = list()

for(i in 1:length(dfsToTest_r)) {
  aracneSplinedPairwiseResults_r_Kim_Strong[[i]] = evaluateTDAracnePairwise(
    title = paste("Spline_",dfsToTest_r[i]), rounds = randomRoundsAracne, 
    profilesRaw = gse44415_raw[rownames(gse44415_raw) %in% c(sigRSco, sigRRegulon_Kim_Strong),], 
    time =  gse44415_raw_time, rawTime = gse44415_raw_time, splineDFs = dfsToTest_r[i],
    randomScale = sigRRandomScale, randomLength = sigRRandomLength,errorDef =errorDef, regulatorName = sigRSco, regulonNames = sigRRegulon_Kim_Strong, numBins = defaultAracneNumBins)
}
aracneSplinedPairwiseEnd_r_Kim_Strong = proc.time();
aracneSplinedPairwiseTimePerRound_r_Kim_Strong = (aracneSplinedPairwiseEnd_r_Kim_Strong["elapsed"] - aracneSplinedPairwiseStart_r_Kim_Strong["elapsed"]) / ((randomRoundsAracne + 1) * length(dfsToTest_r));


aracneRawPairwiseStart_r_Kim_Strong =  proc.time();
aracneRawPairwiseResult_r_Kim_Strong = evaluateTDAracnePairwise(
  title = "Raw",rounds = randomRoundsAracne, 
  profilesRaw = gse44415_raw[rownames(gse44415_raw) %in% c(sigRSco, sigRRegulon_Kim_Strong),], 
  rawTime =  gse44415_raw_time, randomScale = sigRRandomScale, randomLength = sigRRandomLength,
  regulatorName = sigRSco, regulonNames = sigRRegulon_Kim_Strong, errorDef = errorDef, numBins = defaultAracneNumBins,
  splineDFs = NULL, time = NULL)
aracneRawPairwiseEnd_r_Kim_Strong =  proc.time();
aracneRawPairwiseTimePerRound_r_Kim_Strong = (aracneRawPairwiseEnd_r_Kim_Strong["elapsed"] - aracneRawPairwiseStart_r_Kim_Strong["elapsed"]) / (randomRoundsAracne + 1);


numCoresUsed = detectCores()[1] - 1; #The script leaves one CPU idle to let you work while it computes
```

### Results for pairwise TD-ARACNE Kim et al. Strong


```
#Run on raw data is compared against the highest definition spline
printTDAracneEvaluationHeader();
```


```
DF  Type    numTested   Regulator       Random      Ratio
```


```
printTDAracnePairwiseEvaluation("None", aracneRawPairwiseResult_r_Kim_Strong, variousSplinesResult_r_kim_strong[[length(variousSplinesResult_r_kim_strong)]])
```


```
None    Direct      96  0.16    15  0.16    15.12   0.9920635
None    Any         96  0.41    39  0.33    32.1    1.214953
None    Direct-L    82  0.15    12  0.16    13.4    0.8955224
None    Any-L       82  0.41    34  0.35    28.5    1.192982
```


```
for(i in 1:length(dfsToTest_r)) {
  printTDAracnePairwiseEvaluation(paste0(dfsToTest_r[i]), aracneSplinedPairwiseResults_r_Kim_Strong[[i]], variousSplinesResult_r_kim_strong[[i]])
}
```


```
3   Direct      96  0.05    5   0.18    17.52   0.2853881
3   Any         96  0.46    44  0.25    24.08   1.827243
3   Direct-L    58  0   0   0.22    12.86   0
3   Any-L       58  0.59    34  0.29    16.74   2.031063
4   Direct      96  0.03    3   0.12    11.52   0.2604167
4   Any         96  0.27    26  0.33    31.62   0.8222644
4   Direct-L    69  0.04    3   0.13    9.3 0.3225806
4   Any-L       69  0.17    12  0.36    24.7    0.48583
5   Direct      96  0.15    14  0.12    11.66   1.200686
5   Any         96  0.27    26  0.34    33  0.7878788
5   Direct-L    71  0.13    9   0.14    9.76    0.9221311
5   Any-L       71  0.24    17  0.39    27.64   0.6150507
6   Direct      96  0.11    11  0.09    8.78    1.252847
6   Any         96  0.34    33  0.27    25.82   1.278079
6   Direct-L    70  0.13    9   0.1 6.9 1.304348
6   Any-L       70  0.3 21  0.29    20.54   1.022395
7   Direct      96  0.24    23  0.15    14.22   1.61744
7   Any         96  0.39    37  0.26    25.4    1.456693
7   Direct-L    74  0.26    19  0.16    12  1.583333
7   Any-L       74  0.41    30  0.29    21.76   1.378676
8   Direct      96  0.25    24  0.16    15.62   1.536492
8   Any         96  0.42    40  0.33    31.66   1.263424
8   Direct-L    75  0.27    20  0.18    13.36   1.497006
8   Any-L       75  0.44    33  0.35    26.12   1.2634
9   Direct      96  0.26    25  0.18    16.84   1.484561
9   Any         96  0.47    45  0.32    31.12   1.446015
9   Direct-L    78  0.27    21  0.19    14.54   1.444292
9   Any-L       78  0.47    37  0.34    26.2    1.412214
10  Direct      96  0.2 19  0.19    18.2    1.043956
10  Any         96  0.42    40  0.34    32.58   1.227747
10  Direct-L    83  0.2 17  0.2 16.56   1.02657
10  Any-L       83  0.43    36  0.36    29.66   1.213756
11  Direct      96  0.22    21  0.2 19.28   1.089212
11  Any         96  0.41    39  0.36    34.1    1.143695
11  Direct-L    82  0.24    20  0.22    17.98   1.112347
11  Any-L       82  0.44    36  0.38    30.92   1.164295
```


```
cat(paste0("Raw time per round (on ", numCoresUsed," cores): ", aracneRawPairwiseTimePerRound_r_Kim_Strong, " seconds\n"))
```


```
Raw time per round (on 7 cores): 30.3223529411765 seconds
```


```
cat(paste0("Splined time per round (on ", numCoresUsed," cores): ", aracneSplinedPairwiseTimePerRound_r_Kim_Strong , " seconds\n"))
```


```
Splined time per round (on 7 cores): 30.6353812636166 seconds
```

LS0tDQp0aXRsZTogIkdlbmV4cGkgZXZhbHVhdGlvbiINCm91dHB1dDogaHRtbF9ub3RlYm9vaw0KLS0tDQoNClRoaXMgUiBub3RlYm9vayBwZXJmb3JtcyB0aGUgZXZhbHVhdGlvbiBvZiBHZW5leHBpIGFnYWluc3QgVEQtQXJhY25lIG9uIHJlZ3Vsb24gaWRlbnRpZmlhY3Rpb24gdGFza3MuIE5vdGUgdGhhdCB0aGUgZnVsbCBldmFsdWF0aW9uIGNhbiB0YWtlIG92ZXIgYSB3ZWVrLiBJZiB5b3Ugd2FudCB0byBkbyBxdWljayByZXJ1biB0byB1bmRlcnN0YW5kIHdoYXQgaXMgZ29pbmcgb24sIHlvdSBjYW4gYWx0ZXIgdGhlIGBgYHNpZ0JSZWd1bG9uTmFtZXNgYGAsIGBgYHNpZ1JSZWd1bG9uX0thbGxpZmlkYXNgYGAsIGBgYHNpZ1JSZWd1bG9uX0tpbWBgYCBhbmQgLCBgYGBzaWdSUmVndWxvbl9LaW1fU3Ryb25nYGBgIHZhcmlhYmxlcyB0byBjb250YWluIGp1c3QgYSBmZXcgZ2VuZXMsIHdoaWNoIHNob3VsZCBnaXZlIHlvdSBhbnN3ZXJzIGluIHJlYXNvbmFibGUgdGltZS4NCg0KIyBTZXR0aW5nIHVwDQoNCldlIHdpbGwga2VlcCBhIGxvdCBvZiBpbnRlcm1lZGlhdGUgcmVzdWx0cyBpbiB0aGUgZm9ybSBvZiBKYXZhIG9iamVjdHMgaW4gbWVtb3J5LCBzbyBsZXQgdXMgaW5jcmVhc2UgdGhlIG1heCBtZW1vcnkuIFBsZWFzZSBzdGFydCB0aGUgbm90ZWJvb2sgaW4gYSBmcmVzaCBzZXNzaW9uLg0KDQpgYGB7ciBzZXR1cH0NCiAgb3B0aW9ucyhqYXZhLnBhcmFtZXRlcnMgPSAiLVhteDIwNDhtIikNCiAgZGV2ZWxSdW4gPSBGQUxTRTsNCmBgYA0KDQpGaXJzdCB3ZSBpbnN0YWxsIGFuZCBzdGFydCBhbGwgdGhlIHJlcXVpcmVkIGxpYnJhcmllcy4NCg0KYGBge3J9DQogICMgSGVscGVyIGZ1bmN0aW9uIHRvIGluc3RhbGwgcGFja2FnZXMgaWYgdGhleSBhcmUgbm90IGF2YWlsYWJsZQ0KICBwa2dUZXN0IDwtIGZ1bmN0aW9uKHgpDQogIHsNCiAgICBpZiAoIXJlcXVpcmUoeCxjaGFyYWN0ZXIub25seSA9IFRSVUUpKQ0KICAgIHsNCiAgICAgIGluc3RhbGwucGFja2FnZXMoeCxkZXA9VFJVRSkNCiAgICAgICAgaWYoIXJlcXVpcmUoeCxjaGFyYWN0ZXIub25seSA9IFRSVUUpKSBzdG9wKCJQYWNrYWdlIG5vdCBmb3VuZCIpDQogICAgfQ0KICB9DQoNCiAgIyBJbnN0YWxsIGFuZCBsb2FkIEdlbmV4cGkgZnJvbSBHaXRIdWIgdmllIGRldnRvb2xzDQogIGlmKCFkZXZlbFJ1bikgew0KICAgIHBrZ1Rlc3QoImRldnRvb2xzIikNCiAgICBpbnN0YWxsX2dpdGh1YigiY2FzLWJpb2luZi9nZW5leHBpIiwgcmVmID0gImJtYy1yZWxlYXNlIiwgc3ViZGlyPSJycGFja2FnZSIpDQogIH0NCg0KICAjIEluc3RhbGwgYW5kIGxvYWQgdGhlIHBhY2thZ2VzIHJlcXVpcmVkIGZvciBldmFsdWF0aW9uIGFnYWluc3QgVEQtQXJhY25lDQogIHNvdXJjZSgiaHR0cHM6Ly9iaW9jb25kdWN0b3Iub3JnL2Jpb2NMaXRlLlIiKQ0KICBiaW9jTGl0ZShjKCJCaW9iYXNlIiwiVERBUkFDTkUiLCAiUkJHTCIpKQ0KICBsaWJyYXJ5KEJpb2Jhc2UpDQogIGxpYnJhcnkoVERBUkFDTkUpDQogIGxpYnJhcnkoUkJHTCkNCiAgDQogIHBrZ1Rlc3QoInNwbGluZXMiKQ0KICBwa2dUZXN0KCJmb3JlYWNoIikNCiAgcGtnVGVzdCgiZG9QYXJhbGxlbCIpDQogIHBrZ1Rlc3QoInJKYXZhIikNCiAgcGtnVGVzdCgiZ2dwbG90MiIpDQoNCmBgYA0KDQoNCkdlbmV4cGkgcmVsaWVzIG9uIE9wZW5DTCwgc28gd2UgbmVlZCB0byBjaGVjayB0aGUgYXZhaWxhYmxlIE9wZW5DTCBkZXZpY2VzOg0KDQpgYGB7cn0NCmxpc3RPcGVuQ0xEZXZpY2VzKCkNCmBgYA0KSWYgdGhlIGxpc3QgaXMgZW1wdHkgb3IgdGhlIGNhbGwgZW5kcyBpbiBhbiBlcnJvciwgaXQgaXMgcHJvYmFibHkgYmVjYXVzZSB5b3VyIGNvbXB1dGVyIGhhcyBubyBPcGVuQ0wgZHJpdmVyIGluc3RhbGxlZC4gQWxtb3N0IGFsbCByZWNlbnQgR1BVcyBhbmQgQ1BVcyAocHJvY2Vzc29ycykgc3VwcG9ydCBPcGVuQ0wsIHNvIHlvdSBzaG91bGQgYmUgYWJsZSB0byBydW4gR2VuZXhwaS4NCg0KKiBGb3IgR1BVcywgdHJ5IHVwZGF0aW5nIHlvdXIgZGV2aWNlIGRyaXZlci4gV2UgYWxzbyBlbmNvdW50ZXJlZCBjYXNlcyB3aGVyZSBHUFVzIHN0YXJ0ZWQgYmVpbmcgYXZhaWxhYmxlIG9ubHkgYWZ0ZXIgaW5zdGFsbGluZyBhIENQVSBkcml2ZXIgKHNlZSBuZXh0IGJ1bGxldCkuDQoqIEZvciBDUFVzICh0aGUgdXN1YWwgcHJvY2Vzc29ycyksIHlvdSBuZWVkIHRvIGluc3RhbGwgc3BlY2lmaWMgZHJpdmVycy4gQXMgb2YgMjAxNy0wNS0yMiwgZHJpdmVycyBmb3IgSW50ZWwgQ1BVcyBjYW4gYmUgZG93bmxvYWRlZCBhdCBodHRwczovL3NvZnR3YXJlLmludGVsLmNvbS9lbi11cy9hcnRpY2xlcy9vcGVuY2wtZHJpdmVycyNsYXRlc3RfQ1BVX3J1bnRpbWUgLSAoeW91IHdhbnQg4oCccnVudGltZS1vbmx54oCdKS4gQU1E4oCZcyBkcml2ZXJzIGNhbiBiZSBmb3VuZCBhdCBodHRwOi8vc3VwcG9ydC5hbWQuY29tL2VuLXVzL2tiLWFydGljbGVzL1BhZ2VzL09wZW5DTDItRHJpdmVyLmFzcHguDQoNCklmIHRoZSBsaXN0IGlzIG5vbi1lbXB0eSwgeW91IGNhbiBjaG9vc2Ugd2hpY2ggZGV2aWNlIHRvIHVzZSBmb3IgY29tcHV0YXRpb24uIFRoZSBiZXN0IGRldmljZXMgdG8gdXNlIHdpdGggR2VuZXhwaSBhcmUgKGluIHRoZSBmb2xsb3dpbmcgb3JkZXIpOg0KDQoqIEEgZGVkaWNhdGVkIGdyYXBoaWNzIGNhcmQgKEdQVSkgZm9yIGNvbXB1dGluZyAobm90IGNvbm5lY3RlZCB0byBhIGRpc3BsYXkpDQoqIEEgWGVvbiBQaGkgY2FyZA0KKiBZb3VyIHByb2Nlc3NvciAoQ1BVKQ0KKiBBIEdQVSBjb25uZWN0ZWQgdG8gYSBkaXNwbGF5DQoNCldoaWxlIEdQVXMgd2lsbCBydW4gR2VuZXhwaSB0aGUgZmFzdGVzdCwgdXNpbmcgYSBHUFUgY29ubmVjdGVkIHRvIGEgZGlzcGxheSBpcyBkaXNjb3VyYWdlZCBhcyBpdCBpbnRlcmZlcmVzIHdpdGggdGhlIG9wZXJhdGluZyBzeXN0ZW0gKE9TKSBhbmQgbWF5IGNhdXNlIHlvdXIgY29tcHV0ZXIgdG8gZnJlZXplIGFuZC9vciB0aGUgY29tcHV0YXRpb25zIHRvIGJlIHJlc2V0IGJ5IHRoZSBvcGVyYXRpbmcgc3lzdGVtLiBJZiB5b3UgYWJzb2x1dGVseSBuZWVkIHRvIHVzZSB0aGUgR1BVIHJ1bm5pbmcgeW91ciBkaXNwbGF5LCBhbmQgdXNlIFdpbmRvd3MsIHlvdSBtYXkgd2FudCB0byBkaXNhYmxlIFtURFIgLSBUaW1lb3V0IGRldGVjdGlvbiBhbmQgcmVjb3ZlcnldKGh0dHBzOi8vbXNkbi5taWNyb3NvZnQuY29tL2VuLXVzL2xpYnJhcnkvd2luZG93cy9oYXJkd2FyZS9mZjU2OTkxOCh2PXZzLjg1KS5hc3B4KSAoZXhwZXJ0IG9ubHkpLg0KDQpXaXRoIHRoYXQgaW4gbWluZCwgbGV0IHVzIHNlbGVjdCBhIGRldmljZS4NCg0KYGBge3J9DQojU2VsZWN0IHRoZSBkZXZpY2UgYXV0b21hdGljYWxseQ0KZGV2aWNlU3BlY3MgPSBnZXREZXZpY2VTcGVjcygpDQoNCiNTZWxlY3QgdGhlIGJlc3QgR1BVIGRldmljZQ0KI2RldmljZVNwZWNzID0gZ2V0RGV2aWNlU3BlY3MoZGV2aWNlVHlwZSA9ICJncHUiKQ0KDQojU2VsZWN0IHRoZSBiZXN0IENQVSBkZXZpY2UNCiNkZXZpY2VTcGVjcyA9IGdldERldmljZVNwZWNzKGRldmljZVR5cGUgPSAicHJvY2Vzc29yIikNCg0KI1NlbGVjdCBkZXZpY2UgYnkgSUQgcmVwb3J0ZWQgaW4gbGlzdE9wZW5DTERldmljZXMoKQ0KI2RldmljZVNwZWNzID0gZ2V0RGV2aWNlU3BlY3MoZGV2aWNlID0gMykNCg0KI0Rpc3BsYXkgdGhlIHNlbGVjdGVkIGRldmljZQ0KaWYoaXMubnVsbChkZXZpY2VTcGVjcykpIHsNCiAgc3RvcCgiSW52YWxpZCBkZXZpY2UiKQ0KfSBlbHNlIHsNCiAgcGFzdGUwKCJVc2luZyBkZXZpY2U6ICIsIGdldEphdmFEZXZpY2VTcGVjcyhkZXZpY2VTcGVjcykkZ2V0RGV2aWNlKCkkdG9TdHJpbmcoKSkNCn0NCmBgYA0KDQojIExvYWQgdGhlIHNpZ0IgZGF0YSBmcm9tIEdTRTY4NjUNCg0KVGhlIG9yaWdpbmFsIHNvdXJjZSBvZiBkYXRhOiAoaHR0cHM6Ly93d3cubmNiaS5ubG0ubmloLmdvdi9nZW8vcXVlcnkvYWNjLmNnaT9hY2M9R1NFNjg2NSkNCg0KYGBge3J9DQp0ZW1wIDwtIHRlbXBmaWxlKCk7DQpkb3dubG9hZC5maWxlKCJmdHA6Ly9mdHAubmNiaS5ubG0ubmloLmdvdi9nZW8vc2VyaWVzL0dTRTZubm4vR1NFNjg2NS9tYXRyaXgvR1NFNjg2NV9zZXJpZXNfbWF0cml4LnR4dC5neiIsIHRlbXApDQpnc2U2ODY1X3Jhd19kZiA9IHJlYWQuZGVsaW0oZ3pmaWxlKHRlbXApLCBjb21tZW50LmNoYXIgPSAiISIpICNJbnRlcm1lZGlhdGUgZGF0YSBmcmFtZSByZXByZXNlbnRhdGlvbg0KDQojIFJhdyBwcm9maWxlIGRhdGEuIFdlIHNjYWxlIGJ5IDEwMDAgdG8gZ2V0IHRvIG1vcmUgd2llbGR5IHZhbHVlcw0KZ3NlNjg2NV9yYXcgPSBhcy5tYXRyaXgoZ3NlNjg2NV9yYXdfZGZbLDI6MTVdKSAvIDEwMDANCnJvd25hbWVzKGdzZTY4NjVfcmF3KSA9IGdzZTY4NjVfcmF3X2RmJElEX1JFRg0KDQojVGltZXMgKGluIG1pbnV0ZXMpIGZvciB0aGUgaW5kaXZpZHVhbCBzYW1wbGVzDQpnc2U2ODY1X3Jhd190aW1lID0gYygwLDUsMTAsMTUsMjAsMjUsMzAsNDAsNTAsNjAsNzAsODAsOTAsMTAwKQ0KY29sbmFtZXMoZ3NlNjg2NV9yYXcpIDwtIHNhcHBseShnc2U2ODY1X3Jhd190aW1lLCAgRlVOID0gZnVuY3Rpb24oeCkgeyBwYXN0ZTAoeCwibWluIil9KQ0KDQojV2Ugd2lsbCBjb21wdXRlIGF0IDEgbWludXRlIHJlc29sdXRpb24NCnNtb290aFRpbWUgPSAwOjEwMA0KZXJyb3JEZWYgPSBsaXN0KHJlbGF0aXZlID0gMC4yLCBhYnNvbHV0ZSA9IDAsIG1pbmltYWwgPSAwLjUpDQoNCiMgVGhlcmUgYXJlIGEgZmV3IGdlbmVzIHRoYXQgaGF2ZSBOQSB2YWx1ZXMgZm9yIHRoZSBmaXJzdCBvciBzZWNvbmQgbWVhc3VyZW1lbnQNCmFwcGx5KGdzZTY4NjVfcmF3LCBNQVJHSU4gID0gMiwgRlVOID0gZnVuY3Rpb24oeCkgeyBzdW0oaXMubmEoeCkpIH0pDQoNCmBgYA0KQWJvdmUsIHRoZSBudW1iZXIgb2YgdmFsdWVzIHRoYXQgYXJlIE5BIGZvciBlYWNoIHRpbWUgcG9pbnQgaXMgc2hvd24uIFRob3NlIHdpbGwgYmUgaW1wdXRlZC4NCg0KYGBge3J9DQojIEZvciB0aGUgZmlyc3QgbWVhc3VyZW1lbnQsIHdlIGNhbiBleHBlY3QgdGhlIHZhbHVlIHRvIGJlIDAgKHRoZSBzZXJpZXMgYXJlIGZyb20gZ2VybWluYXRpb24pDQpnc2U2ODY1X3Jhd1tpcy5uYShnc2U2ODY1X3Jhd1ssMV0pLDFdID0gMA0KDQojIEZvciB0aGUgc2Vjb25kIG1lYXN1cmVtZW50IHdlIHdpbGwgZ28gd2l0aCBhIGxpbmVhciBpbnRlcnBvbGF0aW9uDQpuYTIgPSBpcy5uYShnc2U2ODY1X3Jhd1ssMl0pDQpnc2U2ODY1X3Jhd1tuYTIsMl0gPSAwLjUgKiBnc2U2ODY1X3Jhd1tuYTIsMV0gKyAwLjUgKiBnc2U2ODY1X3Jhd1tuYTIsM10NCg0KDQoNCiMgR2VuZXMgaW4gdGhlIHJlZ3Vsb24gYWNjb3JkaW5nIHRvIGh0dHA6Ly9zdWJ0aXdpa2kudW5pLWdvZXR0aW5nZW4uZGUvd2lraS9pbmRleC5waHAvU2lnQl9yZWd1bG9uIGFzIG9mIDIwMTctMDItMDkNCnNpZ0JSZWd1bG9uTmFtZXMgPSBjKCJhYWciLCAiYWxkWSIsICJibXIiLCAiYm1yUiIsICJibXJVIiwgImJvZkMiLCAiY2RkIiwgImNoYUEiLCAiY2xwQyIsICJjbHBQIiwgImNvckEiLCAiY3NiQSIsICJjc2JCIiwgImNzYkMiLCAiY3NiRCIsICJjc2JYIiwgImN0YyIsICJjdHNSIiwgImN5cEMiLCAiZGlzQSIsICJkcHMiLCAiZXJhIiwgImdhYkQiLCAiZ3NpQiIsICJnc3BBIiwgImd0YUIiLCAiZ3VhRCIsICJocGYiLCAiaW9sVyIsICJpc3BEIiwgImlzcEYiLCAia2F0RSIsICJrYXRYIiwgIm1jc0EiLCAibWNzQiIsICJtZ3NSIiwgIm1ocU8iLCAibWhxUCIsICJuYWRFIiwgIm5oYVgiLCAib2hyQiIsICJvcHVFIiwgInBob1AiLCAicGhvUiIsICJwbHNDIiwgInJhZEEiLCAicm5yIiwgInJwbUVCIiwgInJzYlJEIiwgInJzYlYiLCAicnNiVyIsICJyc2JYIiwgICJzb2RBIiwgInNwbzBFIiwgInNwb1ZDIiwgInNweCIsICJ0cnhBIiwgInlhYUgiLCAieWFhSSIsICJ5YWNMIiwgInlieUIiLCAieWNiUCIsICJ5Y2RGIiwgInljZEciLCAieWNlQyIsICJ5Y2VEIiwgInljZUUiLCAieWNlRiIsICJ5Y2VHIiwgInljZUgiLCAieWRhRCIsICJ5ZGFFIiwgInlkYUYiLCAieWRhRyIsICJ5ZGFKIiwgInlkYUsiLCAieWRhTCIsICJ5ZGFNIiwgInlkYU4iLCAieWRhUCIsICJ5ZGFTIiwgInlkYVQiLCAieWRiRCIsICJ5ZGVDIiwgInlkaEsiLCAieWVyRCIsICJ5ZmhEIiwgInlmaEUiLCAieWZoRiIsICJ5ZmhLIiwgInlmaEwiLCAieWZoTSIsICJ5ZmtEIiwgInlma0giLCAieWZrSSIsICJ5ZmtKIiwgInlma00iLCAieWZrUyIsICJ5ZmtUIiwgInlmbEEiLCAieWZsSCIsICJ5ZmxUIiwgInlneEIiLCAieWhjTSIsICJ5aGRGIiwgInloZE4iLCAieWh4RCIsICJ5aXRUIiwgInlqYkMiLCAieWpnQiIsICJ5amdDIiwgInlqZ0QiLCAieWp6RSIsICJ5a2dBIiwgInlrZ0IiLCAieWt1VCIsICJ5a3pJIiwgInlseFAiLCAieW16QiIsICJ5b2FBIiwgInlvY0IiLCAieW9jSyIsICJ5b3hCIiwgInlveEMiLCAieXB1QiIsICJ5cHVDIiwgInlwdUQiLCAieXFoQiIsICJ5cWhQIiwgInlxaFEiLCAieXFqTCIsICJ5cmFBIiwgInlzZEIiLCAieXNuRiIsICJ5dGFCIiwgInl0a0wiLCAieXR4RyIsICJ5dHhIIiwgInl0eEoiLCAieXVnVSIsICJ5dXpBIiwgInl2YUsiLCAieXZnTiIsICJ5dmdPIiwgInl2ckUiLCAieXdpRSIsICJ5d2pDIiwgInl3bEIiLCAieXdtRSIsICJ5d21GIiwgInl3c0IiLCAieXd0RyIsICJ5d3pBIiwgInl4YUIiLCAieXhiRyIsICJ5eGlTIiwgInl4akkiLCAieXhqSiIsICJ5eGtPIiwgInl4bkEiLCAieXh6RiIsICJ5eWNEIiwgInljek8iLCAieWRhQyIsICJ5ZWJFIiwgInllYkciLCAieWZsRCIsICJ5ZmxCIiwgInlpc1AiLCAiaXBpIiwgInlqbEIiLCAieWt6TiIsICJzcG8wRSIsICJ5a3RDIiwgInlrekMiLCAicmJmQSIsICJ5dGtDIiwgInl0aUIiLCAibWVuQyIsICJtZW5FIiwgInl1ekgiLCAieXZiRyIsICJ5dnpFIiwgInl3c0EiLCAieXdqQSIsICJ5eWRDIiwgInl5ekciLCAieXl6SCIsICJ5eWJPIikNCg0KDQojY2xlYW51cCB0aGUgaW50ZXJtZWRpYXRlIHJlc3VsdHMNCnJtKGdzZTY4NjVfcmF3X2RmKQ0Kcm0obmEyKQ0Kcm0odGVtcCkNCg0KYGBgDQoNCg0KDQojIFJhbmRvbSBQcm9maWxlcw0KDQpUaGUgcmFuZG9tIHByb2ZpbGVzIGFyZSBkcmF3biBmcm9tIGEgW0dhdXNzaWFuIHByb2Nlc3NdKGh0dHBzOi8vZW4ud2lraXBlZGlhLm9yZy93aWtpL0dhdXNzaWFuX3Byb2Nlc3MpIHdpdGggc3F1YXJlZCBleHBvbmVudGlhbCBrZXJuZWwgYW5kIHplcm8gbWVhbiBmdW5jdGlvbi4gVG8ga2VlcCB0aGUgcHJvZmlsZSBzdHJpY3RseSBwb3NpdGl2ZSwgaXQgaXMgdHJhbnNmb3JtZWQgdmlhICQkZih4KSA9IGxvZygxICsgZV54KSQkDQoNClRoZSBzb3VyY2Ugb2YgdGhlIHJhbmRvbSBwcm9maWxlcyByZWxhdGVkIGZ1bmN0aW9ucyBjYW4gYmUgZm91bmQgYXQgaHR0cHM6Ly9naXRodWIuY29tL2Nhcy1iaW9pbmYvZ2VuZXhwaS9ibG9iL21hc3Rlci9ycGFja2FnZS9SL3JhbmRvbVByb2ZpbGVzLlINCg0KYGBge3J9DQoNCiMgUGxvdCAxMCByYW5kb20gcHJvZmlsZXMgd2l0aCBzY2FsZSA1IGFuZCBsZW5ndGggMjANCnBsb3RSYW5kb21Qcm9maWxlcygxMCwgZ3NlNjg2NV9yYXdfdGltZSwgc2NhbGUgPSA1LCBsZW5ndGggPSAyMCwgbWFpbiA9ICJTYW1wbGVkIHJhbmRvbSBwcm9maWxlcyIsIHlsYWI9IkV4cHJlc3Npb24iKTsNCmBgYA0KDQpIb3cgZG8gd2UgaWRlbnRpZnkgdGhlIGNvcnJlY3Qgc2NhbGUgYW5kIGxlbmd0aCBmb3IgdGhlIEdhdXNzaWFuIHByb2Nlc3M/IExldHMganVzdCBmaW5kIHNvbWUgdGhhdCBsb29rIHNpbWlsYXIgZW5vdWdoIHRvIHRoZSBzaWdCIHByb2ZpbGUuIEFmdGVyIGEgYml0IG9mIGV4cGVyaW1lbnRhdGlvbiB3ZSBjaG9zZSBzY2FsZSA9IDcgYW5kIGxlbmd0aCA9IDIwIG1pbnV0ZXMuIEJlbG93IGlzIGEgc2FtcGxlIG9mIGhvdyB0aG9zZSByYW5kb20gdHJhamVjdG9yaWVzIGxvb2ssIGNvbXBhcmVkIHRvIHRoZSBzaWdCIHByb2ZpbGUgZnJvbSB0aGUgb3JpZ2luYWwgZGF0YS4gKHRoaXMgaXMgc2hvd24gaW4gdGhlIHBhcGVyIGFzIEZpZy4gMikNCg0KYGBge3J9DQpzaWdCUmFuZG9tU2NhbGUgPSA3DQpzaWdCUmFuZG9tTGVuZ3RoID0gMjANCnNpZ0JJbmRleCA9IHdoaWNoKHJvd25hbWVzKGdzZTY4NjVfcmF3KSA9PSAic2lnQiIpDQpwbG90UmFuZG9tUHJvZmlsZXMoMTAsZ3NlNjg2NV9yYXdfdGltZSwgc2NhbGUgPSBzaWdCUmFuZG9tU2NhbGUsIGxlbmd0aCA9IHNpZ0JSYW5kb21MZW5ndGgsIHRydWVUaW1lID0gZ3NlNjg2NV9yYXdfdGltZSwgdHJ1ZVByb2ZpbGUgPSBnc2U2ODY1X3Jhd1tzaWdCSW5kZXgsXSwgbWFpbiA9ICJTYW1wbGVkIHJhbmRvbSBwcm9maWxlcyBhbmQgdGhlIHNpZ0IgcHJvZmlsZSAoZG90cykiLCB5bGFiPSJleHByZXNzaW9uIikNCmBgYA0KDQojIFNwbGluaW5nDQpTcGxpbmluZyBpcyBkb25lIHZpYSBsaW5lYXIgcmVncmVzc2lvbiBvZiBCLXNwbGluZSBiYXNpcy4gVGhlIHNvdXJjZSBmb3Igc3BsaW5pbmcgaXMgaW4gdGhlIGZ1bmN0aW9uIGBgYHNwbGluZVByb2ZpbGVNYXRyaXhgYGAgd2hpY2ggY2FuIGJlIGZvdW5kIGF0IGh0dHBzOi8vZ2l0aHViLmNvbS9jYXMtYmlvaW5mL2dlbmV4cGkvYmxvYi9tYXN0ZXIvcnBhY2thZ2UvUi93b3JrZmxvdy5SIFRoZSBzcGxpbmVzIGFyZSBnZW5lcmF0ZWQgdXNpbmcgdGhlIGBgYGJzYGBgIGZ1bmN0aW9uIGZyb20gdGhlIGBgYHNwbGluZXNgYGAgcGFja2FnZSBhbmQgdGhlIGNvZWZmaWNpZW50cyBhcmUgZm91bmQgdmlhIHN0YW5kYXJkIGBgYGxtYGBgIGZ1bmN0aW9uLiBIZXJlIGlzIGFuIGV4YW1wbGU6DQoNCmBgYHtyfQ0KI0dlbmVyYXRlZCBhIG5vaXN5IG1lYXN1cmVtZXRzDQp0aW1lID0gc2VxKDAsNi4yOCwgYnkgPSAwLjIpDQp0ZXN0UHJvZmlsZSA9IHNpbih0aW1lKSBeIDIgKyBybm9ybShsZW5ndGgodGltZSksIDAsIDAuMSkNCg0KI1NwbGluZSB3aXRoIDMgZGVncmVlcyBvZiBmcmVlZG9tIC0gbm90IHZlcnkgYWNjdXJhdGUNCnNwbGluZUJhc2lzMyA9IGJzKHRpbWUsIGRmPTMsIGRlZ3JlZSA9IDMpDQpzcGxpbmVGaXQzID0gbG0odGVzdFByb2ZpbGUgfiAwICsgc3BsaW5lQmFzaXMzKQ0Kc3BsaW5lZFByb2ZpbGUzID0gc3BsaW5lQmFzaXMzICUqJSBzcGxpbmVGaXQzJGNvZWZmaWNpZW50cw0KbWF0cGxvdCh0aW1lLHNwbGluZWRQcm9maWxlMywgdHlwZSA9ICJsIiwgbWFpbiA9ICJTcGxpbmUgM0RGIiwgeWxpbSA9IGMoMCwxLjEpKQ0KcG9pbnRzKHRpbWUsIHRlc3RQcm9maWxlKQ0KDQojU3BsaW5lIHdpdGggNiBkZWdyZWVzIG9mIGZyZWVkb20gLSBuaWNlbHkgcmVtb3ZlcyBub2lzZSBmcm9tIGRhdGENCnNwbGluZUJhc2lzNiA9IGJzKHRpbWUsIGRmPTYsIGRlZ3JlZSA9IDMpDQpzcGxpbmVGaXQ2ID0gbG0odGVzdFByb2ZpbGUgfiAwICsgc3BsaW5lQmFzaXM2KQ0Kc3BsaW5lZFByb2ZpbGU2ID0gc3BsaW5lQmFzaXM2ICUqJSBzcGxpbmVGaXQ2JGNvZWZmaWNpZW50cw0KbWF0cGxvdCh0aW1lLHNwbGluZWRQcm9maWxlNiwgdHlwZSA9ICJsIiwgbWFpbiA9ICJTcGxpbmUgNkRGIiwgeWxpbSA9IGMoMCwxLjEpKQ0KcG9pbnRzKHRpbWUsIHRlc3RQcm9maWxlKQ0KDQpgYGANCg0KDQojIFRlc3RpbmcgU2lnQiByZWd1bG9uIG9mIEJhY2lsdXMgc3VidGlsaXMNCg0KDQojIyBHZW5leHBpIGluIHRoZSBzaWdCIHJlZ3Vsb24NCg0KVGhlIHNvdXJjZSBmb3IgZnVuY3Rpb25zIHVzZWQgaGVyZSBjYW4gYmUgZm91bmQgYXQNCmh0dHBzOi8vZ2l0aHViLmNvbS9jYXMtYmlvaW5mL2dlbmV4cGkvYmxvYi9tYXN0ZXIvcnBhY2thZ2UvUi9ldmFsdWF0aW9uVG9vbHMuUg0KDQpGaXJzdCBsZXQgdXMgdGFrZSBhIHNuZWFrIHBlZWsgKGp1c3QgMyByYW5kb20gcm91bmRzKSB3aGVuIHdvcmtpbmcgNiBkZWdyZWVzIG9mIGZyZWVkb20gZm9yIHRoZSBzcGxpbmU/IA0KDQpgYGB7cn0NCnJlc3VsdDZERiA9IGV2YWx1YXRlUmFuZG9tRm9yUmVndWxvbihkZXZpY2VTcGVjcywgcm91bmRzID0gMywgcmF3UHJvZmlsZXMgPSBnc2U2ODY1X3JhdyxyZWd1bGF0b3JOYW1lID0gICJzaWdCIiwgcmVndWxvbk5hbWVzID0gc2lnQlJlZ3Vsb25OYW1lcywgdGltZSA9IHNtb290aFRpbWUsIHJhd1RpbWUgPSBnc2U2ODY1X3Jhd190aW1lLCByYW5kb21TY2FsZSA9IHNpZ0JSYW5kb21TY2FsZSwgcmFuZG9tTGVuZ3RoID0gc2lnQlJhbmRvbUxlbmd0aCwgZXJyb3JEZWYgPSBlcnJvckRlZiwgc3BsaW5lREZzID0gNikNCmBgYA0KYGBge3J9DQpwYXN0ZTAoIlByb3BvcnRpb24gb2YgdHJ1ZSByZWd1bGF0aW9ucyBkaXNjb3ZlcmVkOiAiLHJlc3VsdDZERiR0cnVlUmF0aW8pDQpwYXN0ZTAoIk92ZXJhbGwgcHJvcG9ydGlvbiBvZiByZWd1bGF0aW9ucyBieSByYW5kb20gcHJvZmlsZXM6ICIsIHJlc3VsdDZERiRvdmVyYWxsUmFuZG9tUmF0aW8pDQpgYGANCg0KTm93LCBsZXQncyB0ZXN0IGZvciB2YXJpb3VzIHNwbGluZXMgKHdlIGNhbid0IGdldCBsZXNzIERGcyB0aGFuIDMsIDEwIERGcyBkbyBjbGVhcmx5IG92ZXJmaXQpIGFuZCB3aXRoIG1vcmUgcm91bmRzLg0KDQpgYGB7cn0NCmRmc1RvVGVzdCA9IDM6MTANCnJhbmRvbVJvdW5kcyA9IDUwDQpnZW5leHBpU3RhcnQgPSBwcm9jLnRpbWUoKTsNCmdlbmV4cGlPcHRpb25zKHZlcmJvc2UgPSBGQUxTRSkgIyBIaWRlIHVubmVjZXNzYXJ5IG91dHB1dA0KDQp2YXJpb3VzU3BsaW5lc1Jlc3VsdCA9IHRlc3RWYXJpb3VzU3BsaW5lcyhkZXZpY2VTcGVjcywgcm91bmRzID0gcmFuZG9tUm91bmRzLCByYXdQcm9maWxlcyA9IGdzZTY4NjVfcmF3LCByYXdUaW1lID0gZ3NlNjg2NV9yYXdfdGltZSwgdGFyZ2V0VGltZSA9IHNtb290aFRpbWUsIGRmc1RvVGVzdCA9IGRmc1RvVGVzdCwgcmVndWxhdG9yTmFtZSA9ICJzaWdCIiwgcmVndWxvbk5hbWVzID0gc2lnQlJlZ3Vsb25OYW1lcyxyYW5kb21TY2FsZSA9IHNpZ0JSYW5kb21TY2FsZSwgcmFuZG9tTGVuZ3RoID0gc2lnQlJhbmRvbUxlbmd0aCwgZXJyb3JEZWYgPSBlcnJvckRlZiApDQoNCmdlbmV4cGlFbmQgPSBwcm9jLnRpbWUoKTsNCiMgQ2FsY3VsYXRlIHRpbWUgcGVyIHJvdW5kLiBGb3IgZWFjaCBzcGxpbmUgKGRmKSB2YXJpYW50IEkgaGF2ZSAxIG5vbi1yYW5kb20gcm91bmQgaW4gYWRkaXRpb24gdG8gdGhlIHJhbmRvbSByb3VkbnMNCmdlbmV4cGlUaW1lUGVyUm91bmQgPSAoZ2VuZXhwaUVuZFsiZWxhcHNlZCJdIC0gZ2VuZXhwaVN0YXJ0WyJlbGFwc2VkIl0pIC8gKGxlbmd0aChkZnNUb1Rlc3QpICogKHJhbmRvbVJvdW5kcyArIDEpKQ0Kc2F2ZS5pbWFnZSgiLlJEYXRhIikNCmBgYA0KDQpgYGB7cn0NCnByaW50VmFyaW91c1NwbGluZXNSZXN1bHRzSGVhZGVyKCkNCnByaW50VmFyaW91c1NwbGluZXNSZXN1bHRzKHZhcmlvdXNTcGxpbmVzUmVzdWx0KQ0KY2F0KHBhc3RlMCgiR2VuZXhwaSB0aW1lIHBlciByb3VuZDogIiwgZ2VuZXhwaVRpbWVQZXJSb3VuZCwgIiBzZWNvbmRzXG4iKSkNCmBgYA0KDQoNCkxldCdzIHBsb3QgaGlzdG9ncmFtIG9mIHRoZSByYW5kb20gcmVzdWx0cyBmb3IgNkRGczoNCmBgYHtyfQ0KaGlzdCh2YXJpb3VzU3BsaW5lc1Jlc3VsdFtbd2hpY2goZGZzVG9UZXN0ID09IDYpXV0kcmVzdWx0JHJhbmRvbVJhdGlvcywgbWFpbj0iIiwgeGxhYiA9ICJQcm9wb3J0aW9uIHByZWRpY3RlZCByZWd1bGF0ZWQgYnkgcmFuZG9tIHByb2ZpbGUuIikNCmBgYA0KDQpXZSBzZWUgdGhhdCB2ZXJ5IGZldyByYW5kb20gcHJvZmlsZXMgY2FuIG1pbWljIHRoZSBiZWhhdmlvciBvZiBzaWdCIGFuZCBtb3N0IGFyZSBwb29ybHkgcHJlZGljdGVkLg0KDQojIyBUZXN0aW5nIFRELUFyYWNuZSBpbiB0aGUgc2lnQiByZWd1bG9uDQoNCldlIHRyaWVkIFRELUFyYWNuZSBpbiB0d28gbW9kZXM6IHBhaXJ3aXNlIGFuZCBzaW1wbGUuIEluIHRoZSBzaW1wbGUgbW9kZSwgVEQtQXJhY25lIGlzIHJ1biBvbiB0aGUgd2hvbGUgcmVndWxvbiBhdCBvbmNlLiBUaGlzIGhvd2V2ZXIgcHJvdmVkIHJhdGhlciBzbG93IGFuZCBkaWRuJ3QgZ2V0IHZlcnkgZ29vZCByZXN1bHRzLiBTbyB3ZSBpbnN0ZWFkIHJhbiBURC1BcmFjbmUgc2ltaWxhcmx5IHRvIHRoZSB3YXkgd2UgdXNlIEdlbmV4cGkgLSBhIHNlcGFyYXRlIHJ1biBmb3IgZXZlcnkgVEYtVGFyZ2V0IGNvbWJpbmF0aW9uLiBTaW5jZSBURC1BcmFjbmUgcHJvZHVjZSBvcmllbnRlZCBlZGdlcywgd2UgaGF2ZSB0d28gd2F5cyB0byBjbGFzc2lmeSB0aGUgcmVzdWx0cyAtIGVpdGhlciB3ZSBjb25zaWRlciByZWd1bGF0aW9uIGFzIHByZWRpY3RlZCBpZiB0aGUgZWRnZSBoYXMgdGhlIGNvcnJlY3QgZGlyZWN0aW9uIChsYWJlbGxlZCAiRGlyZWN0IiksIG9yIHdlIGFyZSBzaW1wbHkgaW50ZXJlc3RlZCBpbiBwcmVzZW5jZSB2ZXJzdXMgYWJzZW5jZSBvZiBhbiBlZGdlIChsYWJlbGxlZCAiQW55IikuIEZ1cnRoZXIgd2UgY2FuIHRlc3QgdGhlIHdob2xlIHJlZ3Vsb24gb3Igb25seSB0aGUgZ2VuZXMgdGVzdGVkIGJ5IEdlbmV4cGkgKGkuZS4gcmVtb3ZlIGdlbmVzIHRoYXQgZG8gbm90IGNoYW5nZSBvciBjYW4gYmUgZml0IGJ5IGNvbnN0YW50IHN5bnRoZXNpcykuDQoNClRoZSBzb3VyY2VzIGZvciB0aGUgZnVuY3Rpb25zIHVzZWQgaW4gdGhpcyBzZWN0aW9uIGFuZCByZWxhdGVkIGNvZGUgY2FuIGJlIGZvdW5kIGF0DQpodHRwczovL2dpdGh1Yi5jb20vY2FzLWJpb2luZi9nZW5leHBpL2Jsb2IvbWFzdGVyL3JwYWNrYWdlL1IvdGRhcmFjbmVCcmlkZ2UuUg0KYGBge3J9DQojQXJhY25lIHJlcXVpcmVzIHVuaWZvcm0gdGltZSBpbnRlcnZhbHMNCmFyYWNuZVNtb290aFRpbWVJbmRpY2VzID0gYygxLDExLDIxLDMxLDQxLDUxLDYxLDcxLDgxLDkxLDEwMSkNCmFyYWNuZVJhd1RpbWVJbmRpY2VzID0gYygxLDMsNSw3OjE0KQ0KI1RoaXMgd2FzIGNob3NlbiBhcyB0aGUgbWluaW1hbCB2YWx1ZSB0aGF0IGRvZXMgbm90IHJpc2Ugd2FybmluZ3MNCmRlZmF1bHRBcmFjbmVOdW1CaW5zID0gMTANCg0KcmFuZG9tUm91bmRzQXJhY25lID0gNTANCmBgYA0KDQojIFRELUFyYWNuZSBpbiBzaW1wbGUgbW9kZSBvbiBTaWdCDQoNCkZvciBjb21wbGV0ZW5lc3MsIGhlcmUgaXMgdGhlIGNvZGUgdG8gcnVuIFRELUFyYWNuZSBpbiBzaW1wbGUgbW9kZSAob24gdGhlIHdob2xlIHNpZ0IgcmVndWxvbikuIFRoaXMgdGFrZXMgZGF5cyB0byBjb21wdXRlISBUaGUgb3V0cHV0IGhhcyB0d28gY2F0ZWdvcmllczogIkRvd25zdHJlYW0iIHdoZXJlIGV2ZXJ5IGdlbmUgdGhhdCBoYXMgYSBkaXJlY3RlZCBwYXRoIGZyb20gdGhlIHJlZ3VsYXRvciBpcyB0cmVhdGVkIGFzIHJlZ3VsYXRlZCBhbmQgIkNvbm5lY3RlZCIgd2hlcmUgdGhlIHBhdGggbmVlZCBub3QgYmUgZGlyZWN0ZWQuIFRha2luZyBvbmx5IHRoZSBpbW1lZGlhdGUgY29ubmVjdGlvbnMgb2YgdGhlIHJlZ3VsYXRvciBoYWQgdmVyeSBsaXR0bGUgcGVyZm9ybWFuY2UuIChOb3RlIHRoYXQgVEQtQXJhY25lIGlzIHNvbHZpbmcgYSBtdWNoIG1vcmUgZGlmZmljdWx0IHRhc2sgaGVyZSkNCg0KYGBge3J9DQogICNBcmFjbmUgaW4gc2ltcGxlIG1vZGUgaXMgdG9vIHNsb3cNCiAgcmFuZG9tUm91bmRzQXJhY25lU2ltcGxlID0gMjANCiAgZGZzVG9UZXN0X3NpbXBsZSA9IGRmc1RvVGVzdDsNCiAgYXJhY25lU3BsaW5lZFN0YXJ0ID0gIHByb2MudGltZSgpOw0KICBhcmFjbmVTcGxpbmVkUmVzdWx0cyA9IGxpc3QoKQ0KICBmb3IoaSBpbiAxOmxlbmd0aChkZnNUb1Rlc3Rfc2ltcGxlKSkgew0KICAgIGFyYWNuZVNwbGluZWRSZXN1bHRzW1tpXV0gPSBldmFsdWF0ZVREQXJhY25lKA0KICAgICAgcm91bmRzID0gcmFuZG9tUm91bmRzQXJhY25lU2ltcGxlLCBwcm9maWxlc1JhdyA9IGdzZTY4NjVfcmF3LCB0aW1lID0gc21vb3RoVGltZVthcmFjbmVTbW9vdGhUaW1lSW5kaWNlc10sIA0KICAgICAgcmF3VGltZSA9IGdzZTY4NjVfcmF3X3RpbWUsIHNwbGluZURGcyA9IGRmc1RvVGVzdF9zaW1wbGVbaV0sDQogICAgICByYW5kb21TY2FsZSA9IHNpZ0JSYW5kb21TY2FsZSwgcmFuZG9tTGVuZ3RoID0gc2lnQlJhbmRvbUxlbmd0aCxlcnJvckRlZiA9ZXJyb3JEZWYsIHJlZ3VsYXRvck5hbWUgPSAic2lnQiIsIHJlZ3Vsb25OYW1lcyA9IHNpZ0JSZWd1bG9uTmFtZXMsIG51bUJpbnMgPSBkZWZhdWx0QXJhY25lTnVtQmlucykNCiAgfQ0KICBhcmFjbmVTcGxpbmVkRW5kID0gcHJvYy50aW1lKCk7DQogIGFyYWNuZVNwbGluZWRUaW1lUGVyUm91bmQgPSAoYXJhY25lU3BsaW5lZEVuZFsiZWxhcHNlZCJdIC0gYXJhY25lU3BsaW5lZFN0YXJ0WyJlbGFwc2VkIl0pIC8gKChyYW5kb21Sb3VuZHNBcmFjbmVTaW1wbGUgKyAxKSAqIGxlbmd0aChkZnNUb1Rlc3Rfc2ltcGxlKSk7DQogIA0KICANCiAgYXJhY25lUmF3U3RhcnQgPSAgcHJvYy50aW1lKCk7DQogIGFyYWNuZVJhd1Jlc3VsdCA9IGV2YWx1YXRlVERBcmFjbmUoDQogICAgICByb3VuZHMgPSByYW5kb21Sb3VuZHNBcmFjbmVTaW1wbGUsIHByb2ZpbGVzUmF3ID0gZ3NlNjg2NV9yYXdbLGFyYWNuZVJhd1RpbWVJbmRpY2VzLCBkcm9wPUZBTFNFXSwgDQogICAgICB0aW1lID0gTlVMTCwgDQogICAgICByYXdUaW1lID0gZ3NlNjg2NV9yYXdfdGltZVthcmFjbmVSYXdUaW1lSW5kaWNlc10sIHNwbGluZURGcyA9IE5VTEwsDQogICAgICByYW5kb21TY2FsZSA9IHNpZ0JSYW5kb21TY2FsZSwgcmFuZG9tTGVuZ3RoID0gc2lnQlJhbmRvbUxlbmd0aCxlcnJvckRlZiA9ZXJyb3JEZWYsIHJlZ3VsYXRvck5hbWUgPSAic2lnQiIsIHJlZ3Vsb25OYW1lcyA9IHNpZ0JSZWd1bG9uTmFtZXMsIG51bUJpbnMgPSBkZWZhdWx0QXJhY25lTnVtQmlucykNCiAgICANCg0KICBhcmFjbmVSYXdFbmQgPSAgcHJvYy50aW1lKCk7DQogIGFyYWNuZVJhd1RpbWVQZXJSb3VuZCA9IChhcmFjbmVSYXdFbmRbImVsYXBzZWQiXSAtIGFyYWNuZVJhd1N0YXJ0WyJlbGFwc2VkIl0pIC8gKHJhbmRvbVJvdW5kc0FyYWNuZVNpbXBsZSArIDEpOw0KICANCiAgc2F2ZS5pbWFnZSgiLlJEYXRhIikNCmBgYA0KDQojIyBSZXN1bHRzIGZvciBzaW1wbGUgVEQtQXJhY25lLCBzaWdCDQpgYGB7cn0NCiAgbnVtQ29yZXNVc2VkID0gZGV0ZWN0Q29yZXMoKVsxXSAtIDE7ICNUaGUgc2NyaXB0IGxlYXZlcyBvbmUgQ1BVIGlkbGUgdG8gbGV0IHlvdSB3b3JrIHdoaWxlIGl0IGNvbXB1dGVzDQogIHByaW50VERBcmFjbmVFdmFsdWF0aW9uSGVhZGVyKCk7DQogIHByaW50VERBcmFjbmVFdmFsdWF0aW9uKCJOb25lIiwgYXJhY25lUmF3UmVzdWx0LCB2YXJpb3VzU3BsaW5lc1Jlc3VsdFtbbGVuZ3RoKHZhcmlvdXNTcGxpbmVzUmVzdWx0KV1dKQ0KICBmb3IoaSBpbiAxOmxlbmd0aChkZnNUb1Rlc3Rfc2ltcGxlKSkgew0KICAgIHByaW50VERBcmFjbmVFdmFsdWF0aW9uKGRmc1RvVGVzdF9zaW1wbGVbaV0sIGFyYWNuZVNwbGluZWRSZXN1bHRzW1tpXV0sIHZhcmlvdXNTcGxpbmVzUmVzdWx0W1tpXV0pDQogIH0NCiAgY2F0KHBhc3RlMCgiUmF3IHRpbWUgcGVyIHJvdW5kIChvbiAiLCBudW1Db3Jlc1VzZWQsIiBjb3Jlcyk6ICIsIGFyYWNuZVJhd1RpbWVQZXJSb3VuZCwgIiBzZWNvbmRzXG4iKSkNCiAgY2F0KHBhc3RlMCgiU3BsaW5lZCB0aW1lIHBlciByb3VuZCAob24gIiwgbnVtQ29yZXNVc2VkLCIgY29yZXMpOiAiLCBhcmFjbmVTcGxpbmVkVGltZVBlclJvdW5kLCAiIHNlY29uZHNcbiIpKQ0KDQpgYGANCg0KDQpgYGB7cn0NCg0KYXJhY25lU3BsaW5lZFBhaXJ3aXNlU3RhcnQgPSAgcHJvYy50aW1lKCk7DQphcmFjbmVTcGxpbmVkUGFpcndpc2VSZXN1bHRzID0gbGlzdCgpDQpmb3IoaSBpbiAxOmxlbmd0aChkZnNUb1Rlc3QpKSB7DQogIGFyYWNuZVNwbGluZWRQYWlyd2lzZVJlc3VsdHNbW2ldXSA9IGV2YWx1YXRlVERBcmFjbmVQYWlyd2lzZSgNCiAgICB0aXRsZSA9IHBhc3RlKCJTcGxpbmVfIixkZnNUb1Rlc3RbaV0pLCByb3VuZHMgPSByYW5kb21Sb3VuZHNBcmFjbmUsIHByb2ZpbGVzUmF3ID0gZ3NlNjg2NV9yYXcsIA0KICAgIHRpbWUgPSAgc21vb3RoVGltZVthcmFjbmVTbW9vdGhUaW1lSW5kaWNlc10sIHJhd1RpbWUgPSBnc2U2ODY1X3Jhd190aW1lLCBzcGxpbmVERnMgPSBkZnNUb1Rlc3RbaV0sDQogICAgcmFuZG9tU2NhbGUgPSBzaWdCUmFuZG9tU2NhbGUsIHJhbmRvbUxlbmd0aCA9IHNpZ0JSYW5kb21MZW5ndGgsZXJyb3JEZWYgPWVycm9yRGVmLCByZWd1bGF0b3JOYW1lID0gInNpZ0IiLCByZWd1bG9uTmFtZXMgPSBzaWdCUmVndWxvbk5hbWVzLCBudW1CaW5zID0gZGVmYXVsdEFyYWNuZU51bUJpbnMpDQp9DQphcmFjbmVTcGxpbmVkUGFpcndpc2VFbmQgPSBwcm9jLnRpbWUoKTsNCmFyYWNuZVNwbGluZWRQYWlyd2lzZVRpbWVQZXJSb3VuZCA9IChhcmFjbmVTcGxpbmVkUGFpcndpc2VFbmRbImVsYXBzZWQiXSAtIGFyYWNuZVNwbGluZWRQYWlyd2lzZVN0YXJ0WyJlbGFwc2VkIl0pIC8gKChyYW5kb21Sb3VuZHNBcmFjbmUgKyAxKSAqIGxlbmd0aChkZnNUb1Rlc3QpKTsNCg0KDQphcmFjbmVSYXdQYWlyd2lzZVN0YXJ0ID0gIHByb2MudGltZSgpOw0KYXJhY25lUmF3UGFpcndpc2VSZXN1bHQgPSBldmFsdWF0ZVREQXJhY25lUGFpcndpc2UoDQogIHRpdGxlID0gIlJhdyIscm91bmRzID0gcmFuZG9tUm91bmRzQXJhY25lLCBwcm9maWxlc1JhdyA9IGdzZTY4NjVfcmF3WyxhcmFjbmVSYXdUaW1lSW5kaWNlcywgZHJvcD1GQUxTRV0sIA0KICByYXdUaW1lID0gIGdzZTY4NjVfcmF3X3RpbWVbYXJhY25lUmF3VGltZUluZGljZXNdLCByYW5kb21TY2FsZSA9IHNpZ0JSYW5kb21TY2FsZSwgcmFuZG9tTGVuZ3RoID0gc2lnQlJhbmRvbUxlbmd0aCwNCiAgcmVndWxhdG9yTmFtZSA9ICJzaWdCIiwgcmVndWxvbk5hbWVzID0gc2lnQlJlZ3Vsb25OYW1lcywgZXJyb3JEZWYgPWVycm9yRGVmLCBudW1CaW5zID0gZGVmYXVsdEFyYWNuZU51bUJpbnMsDQogIHNwbGluZURGcyA9IE5VTEwsIHRpbWUgPSBOVUxMKQ0KYXJhY25lUmF3UGFpcndpc2VFbmQgPSAgcHJvYy50aW1lKCk7DQphcmFjbmVSYXdQYWlyd2lzZVRpbWVQZXJSb3VuZCA9IChhcmFjbmVSYXdQYWlyd2lzZUVuZFsiZWxhcHNlZCJdIC0gYXJhY25lUmF3UGFpcndpc2VTdGFydFsiZWxhcHNlZCJdKSAvIChyYW5kb21Sb3VuZHNBcmFjbmUgKyAxKTsNCg0Kc2F2ZS5pbWFnZSgiLlJEYXRhIikNCg0KbnVtQ29yZXNVc2VkID0gZGV0ZWN0Q29yZXMoKVsxXSAtIDE7ICNUaGUgc2NyaXB0IGxlYXZlcyBvbmUgQ1BVIGlkbGUgdG8gbGV0IHlvdSB3b3JrIHdoaWxlIGl0IGNvbXB1dGVzDQpgYGANCg0KIyMjIFJlc3VsdHMgZm9yIHBhaXJ3aXNlIFRELUFSQUNORSwgc2lnQg0KYGBge3J9DQpwcmludFREQXJhY25lRXZhbHVhdGlvbkhlYWRlcigpOw0KI1J1biBvbiByYXcgZGF0YSBpcyBjb21wYXJlZCBhZ2FpbnN0IHRoZSBoaWdoZXN0IGRlZmluaXRpb24gc3BsaW5lDQpwcmludFREQXJhY25lUGFpcndpc2VFdmFsdWF0aW9uKCJOb25lIiwgYXJhY25lUmF3UGFpcndpc2VSZXN1bHQsIHZhcmlvdXNTcGxpbmVzUmVzdWx0W1tsZW5ndGgodmFyaW91c1NwbGluZXNSZXN1bHQpXV0pDQoNCmZvcihpIGluIDE6bGVuZ3RoKGRmc1RvVGVzdCkpIHsNCiAgcHJpbnRUREFyYWNuZVBhaXJ3aXNlRXZhbHVhdGlvbihkZnNUb1Rlc3RbaV0sIGFyYWNuZVNwbGluZWRQYWlyd2lzZVJlc3VsdHNbW2ldXSwgdmFyaW91c1NwbGluZXNSZXN1bHRbW2ldXSkNCn0NCmNhdChwYXN0ZTAoIlJhdyB0aW1lIHBlciByb3VuZCAob24gIiwgbnVtQ29yZXNVc2VkLCIgY29yZXMpOiAiLCBhcmFjbmVSYXdQYWlyd2lzZVRpbWVQZXJSb3VuZCwgIiBzZWNvbmRzXG4iKSkgDQpjYXQocGFzdGUwKCJTcGxpbmVkIHRpbWUgcGVyIHJvdW5kIChvbiAiLCBudW1Db3Jlc1VzZWQsIiBjb3Jlcyk6ICIsIGFyYWNuZVNwbGluZWRQYWlyd2lzZVRpbWVQZXJSb3VuZCAsICIgc2Vjb25kc1xuIikpDQpgYGANCg0KDQojIFRoZSBTaWdSIHJlZ3Vsb24gb2YgUy4gY29lbGljb2xvcg0KVGhpcyBpcyBiYXNpY2FsbHkgdGhlIHNhbWUgYXMgcHJldmlvdXNseSwganVzdCB3aXRoIGRpZmZlcmVudCBkYXRhLg0KDQpgYGB7cn0NCiNUaGUgZGF0YSBhcmUgaW5jbHVkZWQgaW4gdGhlIHBhY2thZ2UNCmRhdGEoZ3NlNDQ0MTVfcmF3KQ0KZ3NlNDQ0MTVfcmF3X3RpbWUgPSBhcy5udW1lcmljKGNvbG5hbWVzKGdzZTQ0NDE1X3JhdykpDQpzbW9vdGhUaW1lX3IgPSAtMzA6MzMwDQoNCnNpZ1JTY28gPSAiNTIxNiINCnNpZ1JSZWd1bG9uX0thbGxpZmlkYXMgPSBjKCIwNTY0IiwgIjA1NjkiLCAiMDU3MCIsICIwODgyIiwgIjA4ODUiLCAiMTM0MCIsICIxMzg0IiwgIjEzOTIiLCAiMTQyMSIsICIxNTEzIiwgIjE4MzEiLCAiMTgzOSIsICIxODY5IiwgIjE5MTkiLCAiMTkyMCIsICIxOTU4IiwgIjE5OTUiLCAiMTk5NiIsICIxOTk3IiwgIjIxNjEiLCAiMjUzNyIsICIyNjE4IiwgIjI2MTkiLCAiMjYzNCIsICIyNjQzIiwgIjI4NDkiLCAiMjkxMCIsICIyOTExIiwgIjI5MTIiLCAiMzA4MyIsICIzMDkxIiwgIjMxNjIiLCAiMzE4NyIsICIzMjAyIiwgIjMyMDYiLCAiMzM3MyIsICIzNDAzIiwgIjM0MTUiLCAiMzc2NCIsICIzODg5IiwgIjM4OTAiLCAiNDAzOSIsICI0MTk4IiwgIjQxOTkiLCAiNDIwNCIsICI0Mjk3IiwgIjQzMTYiLCAiNDUwMSIsICI0NzM2IiwgIjQ3NzAiLCAiNDk1NiIsICI0OTY3IiwgIjQ5NjgiLCAiNTA0MiIsICI1MTYzIiwgIjUxNzciLCAiNTE3OCIsICI1MTg3IiwgIjUyMTciLCAiNTI4NSIsICI1MzU5IiwgIjU0NjUiLCAiNTU0NSIsICI1NjU1IiwgIjU3NTQiLCAiNTc1NSIsICI1Nzk2IiwgIjYwNjEiLCAiNjQyMyIsICI2NDI0IiwgIjY1NTEiLCAiNjU3NyIsICI2NzU5IiwgIjcxMTciLCAiNzIwMyIsICI3NjMyIik7DQoNCg0Kc2lnUlJlZ3Vsb25fS2ltID0gYygiMDU2OSIsICIwNTcwIiwgIjA4ODIiLCAiMDg4NCIsICIwODg1IiwgIjA5MTciLCAiMDk3MyIsICIxMDg0IiwgIjEwODUiLCAiMTE0MiIsICIxMjM4IiwgIjE0MjUiLCAiMTQyNiIsICIxNTEzIiwgIjE1OTgiLCAiMTU5OSIsICIxNjAwIiwgIjE2MTgiLCAiMTYxOSIsICIxNjQzIiwgIjE2NDQiLCAiMTY0NSIsICIxNjQ2IiwgIjE2NDciLCAiMTY0OCIsICIxNzU4IiwgIjE4NjkiLCAiMTkxOSIsICIxOTIwIiwgIjE5MzYiLCAiMTkzNyIsICIxOTM4IiwgIjE5MzkiLCAiMTk1OCIsICIxOTk1IiwgIjE5OTYiLCAiMTk5NyIsICIyMTI0IiwgIjIxNTQiLCAiMjE2MSIsICIyMTYyIiwgIjIxOTQiLCAiMjI1NCIsICIyMzEwIiwgIjIzMzEiLCAiMjQ4MSIsICIyNTM3IiwgIjI1MzgiLCAiMjUzOSIsICIyNTk1IiwgIjI2MTciLCAiMjYxOCIsICIyNjE5IiwgIjI2MzQiLCAiMjYzNSIsICIyNjQyIiwgIjI2NDMiLCAiMjc2MyIsICIyODE2IiwgIjI4NDkiLCAiMjkxMCIsICIyOTExIiwgInMwMiIsICIzMDgzIiwgIjMwOTEiLCAiMzE2MiIsICIzMTg3IiwgIjMyMDYiLCAiMzIwNyIsICIzMjk1IiwgIjMyOTYiLCAiMzM3MyIsICIzNDAzIiwgIjM0NDIiLCAiMzQ0OSIsICIzNDUwIiwgIjM0NTEiLCAiMzUwOSIsICIzNzY0IiwgIjM3NjUiLCAiMzc2NiIsICIzNzY3IiwgIjM4ODkiLCAiMzg5MCIsICI0MDM5IiwgIjQwNDAiLCAiNDEwOSIsICI0MjAzIiwgIjQyMDQiLCAiNDIwNSIsICI0Mjk3IiwgIjQyOTgiLCAiNDI5OSIsICI0NDE4IiwgIjQ0MTkiLCAiNDQyMCIsICI0NTYxIiwgIjQ3NzAiLCAiNDc3MSIsICI0Nzk3IiwgIjQ4MzMiLCAiNDgzNCIsICI0ODM1IiwgIjQ5NTYiLCAiNDk2NiIsICI0OTY3IiwgIjQ5NjgiLCAiNTA0MiIsICI1MDY1IiwgIjUxNjMiLCAiNTE3OCIsICI1MTg3IiwgIjUxODgiLCAiNTIxNyIsICI1Mjg0IiwgIjUyODUiLCAiNTM1NyIsICI1MzU5IiwgIjUzNjAiLCAiNTM2MSIsICI1NDY1IiwgIjU0OTAiLCAiNTU0NSIsICI1NTUyIiwgIjU3MDUiLCAiNTcwNiIsICI1NzA3IiwgIjU3MDgiLCAiNTcwOSIsICI1NzU0IiwgIjU3OTYiLCAiNTgyMCIsICI1ODY0IiwgIjU4NjUiLCAiNjA2MSIsICI2MTI2IiwgIjYxMjciLCAiNjQyMyIsICI2NTUxIiwgIjY3NTkiLCAiNjc2MCIsICI2NzYxIiwgIjY3NjIiLCAiNjc2MyIsICI2NzY0IiwgIjY3NjUiLCAiNjc2NiIsICI2NzY3IiwgIjY3NjgiLCAiNjc2OSIsICI2NzcwIiwgIjY3NzEiLCAiNjc3NSIsICI2Nzc2IiwgIjcxNDAiLCAiNzYzMSIsICI3NjMyIiwgIjc3ODQiLCAiNzc4NSIpDQoNCnNpZ1JSZWd1bG9uX0tpbV9TdHJvbmcgPSBjKCIwNTcwIiwgIjA4ODIiLCAiMDg4NCIsICIwODg1IiwgIjA5MTciLCAiMDk3MyIsICIxMDg0IiwgIjEwODUiLCAiMTE0MiIsICIxMjM4IiwgIjE0MjUiLCAiMTQyNiIsICIxNTEzIiwgIjE2MDAiLCAiMTYxOSIsICIxNjQ4IiwgIjE3NTgiLCAiMTg2OSIsICIxOTIwIiwgIjE5MzYiLCAiMTk1OCIsICIxOTk1IiwgIjE5OTciLCAiMjEyNCIsICIyMTU0IiwgIjIxNjEiLCAiMjE2MiIsICIyMTk0IiwgIjIyNTQiLCAiMjMxMCIsICIyMzMxIiwgIjI0ODEiLCAiMjUzNyIsICIyNTk1IiwgIjI2MTkiLCAiMjYzNCIsICIyNjM1IiwgIjI2NDIiLCAiMjY0MyIsICIyNzYzIiwgIjI4MTYiLCAiMjg0OSIsICIyOTExIiwgInMwMiIsICIzMDgzIiwgIjMwOTEiLCAiMzE2MiIsICIzMTg3IiwgIjMyMDYiLCAiMzIwNyIsICIzMjk2IiwgIjMzNzMiLCAiMzQwMyIsICIzNDQyIiwgIjM0NTAiLCAiMzQ1MSIsICIzNTA5IiwgIjM3NjUiLCAiMzc2NiIsICIzNzY3IiwgIjM4OTAiLCAiNDAzOSIsICI0MDQwIiwgIjQxMDkiLCAiNDIwMyIsICI0MjA0IiwgIjQyOTciLCAiNDQxOSIsICI0NDIwIiwgIjQ1NjEiLCAiNDc3MCIsICI0Nzk3IiwgIjQ4MzUiLCAiNDk1NiIsICI0OTY2IiwgIjQ5NjciLCAiNTA0MiIsICI1MDY1IiwgIjUxNjMiLCAiNTE3OCIsICI1MTg3IiwgIjUxODgiLCAiNTI4NCIsICI1Mjg1IiwgIjUzNTciLCAiNTM1OSIsICI1NDY1IiwgIjU0OTAiLCAiNTU0NSIsICI1NTUyIiwgIjU3MDUiLCAiNTc1NCIsICI1Nzk2IiwgIjU4MjAiLCAiNTg2NCIsICI2MDYxIiwgIjYxMjYiLCAiNjEyNyIsICI2NDIzIiwgIjY1NTEiLCAiNjc1OSIsICI2Nzc1IiwgIjY3NzYiLCAiNzE0MCIsICI3NjMxIiwgIjc2MzIiLCAiNzc4NCIpDQoNCiNGaWx0ZXIgb3V0IHRoZSBnZW5lcyBJIGRvIG5vdCBoYXZlIGluIGRhdGE6DQpzaWdSUmVndWxvbl9LYWxsaWZpZGFzID0gc2lnUlJlZ3Vsb25fS2FsbGlmaWRhc1tzaWdSUmVndWxvbl9LYWxsaWZpZGFzICVpbiUgcm93bmFtZXMoZ3NlNDQ0MTVfcmF3KV0NCnNpZ1JSZWd1bG9uX0tpbSA9IHNpZ1JSZWd1bG9uX0tpbVtzaWdSUmVndWxvbl9LaW0gJWluJSByb3duYW1lcyhnc2U0NDQxNV9yYXcpXQ0Kc2lnUlJlZ3Vsb25fS2ltX1N0cm9uZyA9IHNpZ1JSZWd1bG9uX0tpbV9TdHJvbmdbc2lnUlJlZ3Vsb25fS2ltX1N0cm9uZyAlaW4lIHJvd25hbWVzKGdzZTQ0NDE1X3JhdyldDQoNCmBgYA0KDQpPbmNlIGFnYWluLCB3ZSB0cnkgdG8gZmluZCBhIHNldHRpbmcgZm9yIHRoZSBwcm9maWxlIGdlbmVyYXRvciB0byByb3VnaGx5IG1hdGNoIHRoZSBzaWdSIHByb2ZpbGUuDQoNCmBgYHtyfQ0Kc2lnUlJhbmRvbVNjYWxlID0gMw0Kc2lnUlJhbmRvbUxlbmd0aCA9IDMwDQpzaWdSSW5kZXggPSB3aGljaChyb3duYW1lcyhnc2U0NDQxNV9yYXcpID09IHNpZ1JTY28pDQpwbG90UmFuZG9tUHJvZmlsZXMoMTAsZ3NlNDQ0MTVfcmF3X3RpbWUsIHNpZ1JSYW5kb21TY2FsZSwgc2lnUlJhbmRvbUxlbmd0aCwgdHJ1ZVRpbWUgPSBnc2U0NDQxNV9yYXdfdGltZSwgdHJ1ZVByb2ZpbGUgPSBnc2U0NDQxNV9yYXdbc2lnUkluZGV4LF0sIG1haW4gPSAiU2FtcGxlZCByYW5kb20gcHJvZmlsZXMgYW5kIHRoZSBzaWdSIHByb2ZpbGUgKGRvdHMpIiwgeWxhYj0iZXhwcmVzc2lvbiIpDQpgYGANCg0KDQojIyBHZW5leHBpIGFuZCBTaWdSIHJlZ3Vsb24NCmBgYHtyfQ0KZGZzVG9UZXN0X3IgPSAzOjExDQpnZW5leHBpU3RhcnRfciA9IHByb2MudGltZSgpOw0KZ2VuZXhwaU9wdGlvbnModmVyYm9zZSA9IEZBTFNFKSAjIEhpZGUgdW5uZWNlc3Nhcnkgb3V0cHV0DQp2YXJpb3VzU3BsaW5lc1Jlc3VsdF9yID0gdGVzdFZhcmlvdXNTcGxpbmVzKGRldmljZVNwZWNzLCByb3VuZHMgPSByYW5kb21Sb3VuZHMsIHJhd1Byb2ZpbGVzID0gZ3NlNDQ0MTVfcmF3W3Jvd25hbWVzKGdzZTQ0NDE1X3JhdykgJWluJSBjKHNpZ1JTY28sIHNpZ1JSZWd1bG9uX0thbGxpZmlkYXMpLF0sIHJhd1RpbWUgPSBnc2U0NDQxNV9yYXdfdGltZSwgdGFyZ2V0VGltZSA9IHNtb290aFRpbWVfciwgZGZzVG9UZXN0ID0gZGZzVG9UZXN0X3IsIHJlZ3VsYXRvck5hbWUgPSBzaWdSU2NvLCByZWd1bG9uTmFtZXMgPSBzaWdSUmVndWxvbl9LYWxsaWZpZGFzLHJhbmRvbVNjYWxlID0gc2lnUlJhbmRvbVNjYWxlLCByYW5kb21MZW5ndGggPSBzaWdSUmFuZG9tTGVuZ3RoLCBlcnJvckRlZiA9IGVycm9yRGVmICkNCg0KZ2VuZXhwaUVuZF9yID0gcHJvYy50aW1lKCk7DQojIENhbGN1bGF0ZSB0aW1lIHBlciByb3VuZC4gRm9yIGVhY2ggc3BsaW5lIChkZikgdmFyaWFudCBJIGhhdmUgMSBub24tcmFuZG9tIHJvdW5kIGluIGFkZGl0aW9uIHRvIHRoZSByYW5kb20gcm91ZG5zDQpnZW5leHBpVGltZVBlclJvdW5kX3IgPSAoZ2VuZXhwaUVuZF9yWyJlbGFwc2VkIl0gLSBnZW5leHBpU3RhcnRfclsiZWxhcHNlZCJdKSAvIChsZW5ndGgoZGZzVG9UZXN0KSAqIChyYW5kb21Sb3VuZHMgKyAxKSkNCnNhdmUuaW1hZ2UoIi5SRGF0YSIpDQpgYGANCg0KIyMjIFJlc3VsdHMgZm9yIEdlbmV4cGkgYW5kIHRoZSBTaWdSIHJlZ3Vsb24gKEthbGxpZmlkYXMgZXQgYWwuKQ0KYGBge3J9DQpwcmludFZhcmlvdXNTcGxpbmVzUmVzdWx0c0hlYWRlcigpDQpwcmludFZhcmlvdXNTcGxpbmVzUmVzdWx0cyh2YXJpb3VzU3BsaW5lc1Jlc3VsdF9yKQ0KDQpjYXQocGFzdGUwKCJHZW5leHBpIHRpbWUgcGVyIHJvdW5kOiAiLCBnZW5leHBpVGltZVBlclJvdW5kX3IsICIgc2Vjb25kc1xuIikpDQoNCmBgYA0KDQoNCmBgYHtyfQ0KZ2VuZXhwaVN0YXJ0X3Jfa2ltID0gcHJvYy50aW1lKCk7DQpnZW5leHBpT3B0aW9ucyh2ZXJib3NlID0gRkFMU0UpICMgSGlkZSB1bm5lY2Vzc2FyeSBvdXRwdXQNCnZhcmlvdXNTcGxpbmVzUmVzdWx0X3Jfa2ltID0gdGVzdFZhcmlvdXNTcGxpbmVzKGRldmljZVNwZWNzLCByb3VuZHMgPSByYW5kb21Sb3VuZHMsIHJhd1Byb2ZpbGVzID0gZ3NlNDQ0MTVfcmF3W3Jvd25hbWVzKGdzZTQ0NDE1X3JhdykgJWluJSBjKHNpZ1JTY28sIHNpZ1JSZWd1bG9uX0tpbSksXSwgcmF3VGltZSA9IGdzZTQ0NDE1X3Jhd190aW1lLCB0YXJnZXRUaW1lID0gc21vb3RoVGltZV9yLCBkZnNUb1Rlc3QgPSBkZnNUb1Rlc3RfciwgcmVndWxhdG9yTmFtZSA9IHNpZ1JTY28sIHJlZ3Vsb25OYW1lcyA9IHNpZ1JSZWd1bG9uX0tpbSxyYW5kb21TY2FsZSA9IHNpZ1JSYW5kb21TY2FsZSwgcmFuZG9tTGVuZ3RoID0gc2lnUlJhbmRvbUxlbmd0aCwgZXJyb3JEZWYgPSBlcnJvckRlZiApDQoNCmdlbmV4cGlFbmRfcl9raW0gPSBwcm9jLnRpbWUoKTsNCiMgQ2FsY3VsYXRlIHRpbWUgcGVyIHJvdW5kLiBGb3IgZWFjaCBzcGxpbmUgKGRmKSB2YXJpYW50IEkgaGF2ZSAxIG5vbi1yYW5kb20gcm91bmQgaW4gYWRkaXRpb24gdG8gdGhlIHJhbmRvbSByb3VkbnMNCmdlbmV4cGlUaW1lUGVyUm91bmRfcl9raW0gPSAoZ2VuZXhwaUVuZF9yX2tpbVsiZWxhcHNlZCJdIC0gZ2VuZXhwaVN0YXJ0X3Jfa2ltWyJlbGFwc2VkIl0pIC8gKGxlbmd0aChkZnNUb1Rlc3QpICogKHJhbmRvbVJvdW5kcyArIDEpKQ0Kc2F2ZS5pbWFnZSgiLlJEYXRhIikNCmBgYA0KDQojIyMgUmVzdWx0cyBmb3IgdGhlIHdob2xlIHNpZ1IgcmVndWxvbiBvZiBLaW0gZXQgYWwuDQpgYGB7cn0NCnByaW50VmFyaW91c1NwbGluZXNSZXN1bHRzSGVhZGVyKCkNCnByaW50VmFyaW91c1NwbGluZXNSZXN1bHRzKHZhcmlvdXNTcGxpbmVzUmVzdWx0X3Jfa2ltKQ0KY2F0KHBhc3RlMCgiR2VuZXhwaSB0aW1lIHBlciByb3VuZDogIiwgZ2VuZXhwaVRpbWVQZXJSb3VuZF9yX2tpbSwgIiBzZWNvbmRzXG4iKSkNCmBgYA0KDQpgYGB7cn0NCmdlbmV4cGlTdGFydF9yX2tpbV9zdHJvbmcgPSBwcm9jLnRpbWUoKTsNCmdlbmV4cGlPcHRpb25zKHZlcmJvc2UgPSBGQUxTRSkgIyBIaWRlIHVubmVjZXNzYXJ5IG91dHB1dA0KdmFyaW91c1NwbGluZXNSZXN1bHRfcl9raW1fc3Ryb25nID0gdGVzdFZhcmlvdXNTcGxpbmVzKGRldmljZVNwZWNzLCByb3VuZHMgPSByYW5kb21Sb3VuZHMsIHJhd1Byb2ZpbGVzID0gZ3NlNDQ0MTVfcmF3W3Jvd25hbWVzKGdzZTQ0NDE1X3JhdykgJWluJSBjKHNpZ1JTY28sIHNpZ1JSZWd1bG9uX0tpbV9TdHJvbmcpLF0sIHJhd1RpbWUgPSBnc2U0NDQxNV9yYXdfdGltZSwgdGFyZ2V0VGltZSA9IHNtb290aFRpbWVfciwgZGZzVG9UZXN0ID0gZGZzVG9UZXN0X3IsIHJlZ3VsYXRvck5hbWUgPSBzaWdSU2NvLCByZWd1bG9uTmFtZXMgPSBzaWdSUmVndWxvbl9LaW1fU3Ryb25nLCByYW5kb21TY2FsZSA9IHNpZ1JSYW5kb21TY2FsZSwgcmFuZG9tTGVuZ3RoID0gc2lnUlJhbmRvbUxlbmd0aCwgZXJyb3JEZWYgPSBlcnJvckRlZiApDQoNCmdlbmV4cGlFbmRfcl9raW1fc3Ryb25nID0gcHJvYy50aW1lKCk7DQojIENhbGN1bGF0ZSB0aW1lIHBlciByb3VuZC4gRm9yIGVhY2ggc3BsaW5lIChkZikgdmFyaWFudCBJIGhhdmUgMSBub24tcmFuZG9tIHJvdW5kIGluIGFkZGl0aW9uIHRvIHRoZSByYW5kb20gcm91ZG5zDQpnZW5leHBpVGltZVBlclJvdW5kX3Jfa2ltX3N0cm9uZyA9IChnZW5leHBpRW5kX3Jfa2ltX3N0cm9uZ1siZWxhcHNlZCJdIC0gZ2VuZXhwaVN0YXJ0X3Jfa2ltX3N0cm9uZ1siZWxhcHNlZCJdKSAvIChsZW5ndGgoZGZzVG9UZXN0KSAqIChyYW5kb21Sb3VuZHMgKyAxKSkNCnNhdmUuaW1hZ2UoIi5SRGF0YSIpDQpgYGANCg0KIyMjIFJlc3VsdHMgZm9yIHRoZSAnU1RST05HJyBzdWJwYXJ0IG9mIHNpZ1IgcmVndWxvbiBvZiBLaW0gZXQgYWwuIA0KDQpgYGB7cn0NCnByaW50VmFyaW91c1NwbGluZXNSZXN1bHRzSGVhZGVyKCkNCnByaW50VmFyaW91c1NwbGluZXNSZXN1bHRzKHZhcmlvdXNTcGxpbmVzUmVzdWx0X3Jfa2ltX3N0cm9uZykNCg0KY2F0KHBhc3RlMCgiR2VuZXhwaSB0aW1lIHBlciByb3VuZDogIiwgZ2VuZXhwaVRpbWVQZXJSb3VuZF9yX2tpbV9zdHJvbmcsICIgc2Vjb25kc1xuIikpDQoNCmBgYA0KDQojIyMgU2ltcGxlIFRELUFSQUNORSBvbiBTaWdSLCBLYWxsaWZpZGFzIGV0IGFsLg0KYGBge3J9DQogIGRmc1RvVGVzdF9zaW1wbGVfciA9IGRmc1RvVGVzdF9yOw0KICBhcmFjbmVTcGxpbmVkU3RhcnRfciA9ICBwcm9jLnRpbWUoKTsNCiAgYXJhY25lU3BsaW5lZFJlc3VsdHNfciA9IGxpc3QoKQ0KICBmb3IoaSBpbiAxOmxlbmd0aChkZnNUb1Rlc3Rfc2ltcGxlX3IpKSB7DQogICAgYXJhY25lU3BsaW5lZFJlc3VsdHNfcltbaV1dID0gZXZhbHVhdGVUREFyYWNuZSgNCiAgICAgIHJvdW5kcyA9IHJhbmRvbVJvdW5kc0FyYWNuZVNpbXBsZSwgcHJvZmlsZXNSYXcgPSBnc2U0NDQxNV9yYXcsIHRpbWUgPSBnc2U0NDQxNV9yYXdfdGltZSwgDQogICAgICByYXdUaW1lID0gZ3NlNDQ0MTVfcmF3X3RpbWUsIHNwbGluZURGcyA9IGRmc1RvVGVzdF9zaW1wbGVfcltpXSwNCiAgICAgIHJhbmRvbVNjYWxlID0gc2lnUlJhbmRvbVNjYWxlLCByYW5kb21MZW5ndGggPSBzaWdSUmFuZG9tTGVuZ3RoLGVycm9yRGVmID1lcnJvckRlZiwgcmVndWxhdG9yTmFtZSA9IHNpZ1JTY28sIHJlZ3Vsb25OYW1lcyA9IHNpZ1JSZWd1bG9uX0thbGxpZmlkYXMsIG51bUJpbnMgPSBkZWZhdWx0QXJhY25lTnVtQmlucykNCiAgfQ0KICBhcmFjbmVTcGxpbmVkRW5kX3IgPSBwcm9jLnRpbWUoKTsNCiAgYXJhY25lU3BsaW5lZFRpbWVQZXJSb3VuZF9yID0gKGFyYWNuZVNwbGluZWRFbmRfclsiZWxhcHNlZCJdIC0gYXJhY25lU3BsaW5lZFN0YXJ0X3JbImVsYXBzZWQiXSkgLyAoKHJhbmRvbVJvdW5kc0FyYWNuZVNpbXBsZSArIDEpICogbGVuZ3RoKGRmc1RvVGVzdF9zaW1wbGVfcikpOw0KICANCiAgYXJhY25lUmF3U3RhcnRfciA9ICBwcm9jLnRpbWUoKTsNCiAgYXJhY25lUmF3UmVzdWx0X3IgPSBldmFsdWF0ZVREQXJhY25lKA0KICAgICAgcm91bmRzID0gcmFuZG9tUm91bmRzQXJhY25lU2ltcGxlLCBwcm9maWxlc1JhdyA9IGdzZTQ0NDE1X3JhdywgDQogICAgICB0aW1lID0gTlVMTCwgDQogICAgICByYXdUaW1lID0gZ3NlNDQ0MTVfcmF3X3RpbWUsIHNwbGluZURGcyA9IE5VTEwsDQogICAgICByYW5kb21TY2FsZSA9IHNpZ1JSYW5kb21TY2FsZSwgcmFuZG9tTGVuZ3RoID0gc2lnUlJhbmRvbUxlbmd0aCxlcnJvckRlZiA9ZXJyb3JEZWYsIHJlZ3VsYXRvck5hbWUgPSBzaWdSU2NvLCByZWd1bG9uTmFtZXMgPSBzaWdSUmVndWxvbl9LYWxsaWZpZGFzLCBudW1CaW5zID0gZGVmYXVsdEFyYWNuZU51bUJpbnMpDQogICAgDQoNCiAgYXJhY25lUmF3RW5kX3IgPSAgcHJvYy50aW1lKCk7DQogIGFyYWNuZVJhd1RpbWVQZXJSb3VuZF9yID0gKGFyYWNuZVJhd0VuZF9yWyJlbGFwc2VkIl0gLSBhcmFjbmVSYXdTdGFydF9yWyJlbGFwc2VkIl0pIC8gKHJhbmRvbVJvdW5kc0FyYWNuZVNpbXBsZSArIDEpOw0KYGBgDQoNCiMjIFJlc3VsdHMgZm9yIHNpbXBsZSBURC1BcmFjbmUsIHNpZ1IgDQpgYGB7cn0NCiAgbnVtQ29yZXNVc2VkID0gZGV0ZWN0Q29yZXMoKVsxXSAtIDE7ICNUaGUgc2NyaXB0IGxlYXZlcyBvbmUgQ1BVIGlkbGUgdG8gbGV0IHlvdSB3b3JrIHdoaWxlIGl0IGNvbXB1dGVzDQogIHByaW50VERBcmFjbmVFdmFsdWF0aW9uSGVhZGVyKCk7DQogIHByaW50VERBcmFjbmVFdmFsdWF0aW9uKCJOb25lIiwgYXJhY25lUmF3UmVzdWx0X3IsIHZhcmlvdXNTcGxpbmVzUmVzdWx0X3JbW2xlbmd0aCh2YXJpb3VzU3BsaW5lc1Jlc3VsdF9yKV1dKQ0KICBmb3IoaSBpbiAxOmxlbmd0aChkZnNUb1Rlc3Rfc2ltcGxlX3IpKSB7DQogICAgcHJpbnRUREFyYWNuZUV2YWx1YXRpb24oZGZzVG9UZXN0X3NpbXBsZV9yW2ldLCBhcmFjbmVTcGxpbmVkUmVzdWx0c19yW1tpXV0sIHZhcmlvdXNTcGxpbmVzUmVzdWx0X3JbW2ldXSkNCiAgfQ0KICBjYXQocGFzdGUwKCJSYXcgdGltZSBwZXIgcm91bmQgKG9uICIsIG51bUNvcmVzVXNlZCwiIGNvcmVzKTogIiwgYXJhY25lUmF3VGltZVBlclJvdW5kX3IgLCAiIHNlY29uZHNcbiIpKQ0KICBjYXQocGFzdGUwKCJTcGxpbmVkIHRpbWUgcGVyIHJvdW5kIChvbiAiLCBudW1Db3Jlc1VzZWQsIiBjb3Jlcyk6ICIsIGFyYWNuZVNwbGluZWRUaW1lUGVyUm91bmRfciwgIiBzZWNvbmRzXG4iKSkNCg0KYGBgDQoNCiMjIyBTaW1wbGUgVEQtQVJBQ05FIG9uIFNpZ1IsIEtpbSBldCBhbC4NCmBgYHtyfQ0KICBhcmFjbmVTcGxpbmVkU3RhcnRfcl9raW0gPSAgcHJvYy50aW1lKCk7DQogIGFyYWNuZVNwbGluZWRSZXN1bHRzX3Jfa2ltID0gbGlzdCgpDQogIGZvcihpIGluIDE6bGVuZ3RoKGRmc1RvVGVzdF9zaW1wbGVfcikpIHsNCiAgICBhcmFjbmVTcGxpbmVkUmVzdWx0c19yX2tpbVtbaV1dID0gZXZhbHVhdGVUREFyYWNuZSgNCiAgICAgIHJvdW5kcyA9IHJhbmRvbVJvdW5kc0FyYWNuZVNpbXBsZSwgcHJvZmlsZXNSYXcgPSBnc2U0NDQxNV9yYXcsIHRpbWUgPSBnc2U0NDQxNV9yYXdfdGltZSwgDQogICAgICByYXdUaW1lID0gZ3NlNDQ0MTVfcmF3X3RpbWUsIHNwbGluZURGcyA9IGRmc1RvVGVzdF9zaW1wbGVfcltpXSwNCiAgICAgIHJhbmRvbVNjYWxlID0gc2lnUlJhbmRvbVNjYWxlLCByYW5kb21MZW5ndGggPSBzaWdSUmFuZG9tTGVuZ3RoLGVycm9yRGVmID1lcnJvckRlZiwgcmVndWxhdG9yTmFtZSA9IHNpZ1JTY28sIHJlZ3Vsb25OYW1lcyA9IHNpZ1JSZWd1bG9uX0tpbSwgbnVtQmlucyA9IGRlZmF1bHRBcmFjbmVOdW1CaW5zKQ0KICB9DQogIGFyYWNuZVNwbGluZWRFbmRfcl9raW0gPSBwcm9jLnRpbWUoKTsNCiAgYXJhY25lU3BsaW5lZFRpbWVQZXJSb3VuZF9yX2tpbSA9IChhcmFjbmVTcGxpbmVkRW5kX3Jfa2ltWyJlbGFwc2VkIl0gLSBhcmFjbmVTcGxpbmVkU3RhcnRfcl9raW1bImVsYXBzZWQiXSkgLyAoKHJhbmRvbVJvdW5kc0FyYWNuZVNpbXBsZSArIDEpICogbGVuZ3RoKGRmc1RvVGVzdF9zaW1wbGVfcikpOw0KICANCiAgYXJhY25lUmF3U3RhcnRfcl9raW0gPSAgcHJvYy50aW1lKCk7DQogIGFyYWNuZVJhd1Jlc3VsdF9yX2tpbSA9IGV2YWx1YXRlVERBcmFjbmUoDQogICAgICByb3VuZHMgPSByYW5kb21Sb3VuZHNBcmFjbmVTaW1wbGUsIHByb2ZpbGVzUmF3ID0gZ3NlNDQ0MTVfcmF3LCANCiAgICAgIHRpbWUgPSBOVUxMLCANCiAgICAgIHJhd1RpbWUgPSBnc2U0NDQxNV9yYXdfdGltZSwgc3BsaW5lREZzID0gTlVMTCwNCiAgICAgIHJhbmRvbVNjYWxlID0gc2lnUlJhbmRvbVNjYWxlLCByYW5kb21MZW5ndGggPSBzaWdSUmFuZG9tTGVuZ3RoLGVycm9yRGVmID1lcnJvckRlZiwgcmVndWxhdG9yTmFtZSA9IHNpZ1JTY28sIHJlZ3Vsb25OYW1lcyA9IHNpZ1JSZWd1bG9uX0tpbSwgbnVtQmlucyA9IGRlZmF1bHRBcmFjbmVOdW1CaW5zKQ0KICAgIA0KDQogIGFyYWNuZVJhd0VuZF9yX2tpbSA9ICBwcm9jLnRpbWUoKTsNCiAgYXJhY25lUmF3VGltZVBlclJvdW5kX3Jfa2ltID0gKGFyYWNuZVJhd0VuZF9yWyJlbGFwc2VkIl0gLSBhcmFjbmVSYXdTdGFydF9yWyJlbGFwc2VkIl0pIC8gKHJhbmRvbVJvdW5kc0FyYWNuZVNpbXBsZSArIDEpOw0KDQpgYGANCg0KIyMgUmVzdWx0cyBmb3Igc2ltcGxlIFRELUFyYWNuZSwgc2lnUiBLaW0gZXQgYWwuDQpgYGB7cn0NCiAgbnVtQ29yZXNVc2VkID0gZGV0ZWN0Q29yZXMoKVsxXSAtIDE7ICNUaGUgc2NyaXB0IGxlYXZlcyBvbmUgQ1BVIGlkbGUgdG8gbGV0IHlvdSB3b3JrIHdoaWxlIGl0IGNvbXB1dGVzDQogIHByaW50VERBcmFjbmVFdmFsdWF0aW9uSGVhZGVyKCk7DQogIHByaW50VERBcmFjbmVFdmFsdWF0aW9uKCJOb25lIiwgYXJhY25lUmF3UmVzdWx0X3Jfa2ltLCB2YXJpb3VzU3BsaW5lc1Jlc3VsdF9yX2tpbVtbbGVuZ3RoKHZhcmlvdXNTcGxpbmVzUmVzdWx0X3Jfa2ltKV1dKQ0KICBmb3IoaSBpbiAxOmxlbmd0aChkZnNUb1Rlc3Rfc2ltcGxlX3IpKSB7DQogICAgcHJpbnRUREFyYWNuZUV2YWx1YXRpb24oZGZzVG9UZXN0X3NpbXBsZV9yW2ldLCBhcmFjbmVTcGxpbmVkUmVzdWx0c19yX2tpbVtbaV1dLCB2YXJpb3VzU3BsaW5lc1Jlc3VsdF9yX2tpbVtbaV1dKQ0KICB9DQogIGNhdChwYXN0ZTAoIlJhdyB0aW1lIHBlciByb3VuZCAob24gIiwgbnVtQ29yZXNVc2VkLCIgY29yZXMpOiAiLCBhcmFjbmVSYXdUaW1lUGVyUm91bmRfcl9raW0sICIgc2Vjb25kc1xuIikpDQogIGNhdChwYXN0ZTAoIlNwbGluZWQgdGltZSBwZXIgcm91bmQgKG9uICIsIG51bUNvcmVzVXNlZCwiIGNvcmVzKTogIiwgYXJhY25lU3BsaW5lZFRpbWVQZXJSb3VuZF9yX2tpbSwgIiBzZWNvbmRzXG4iKSkNCg0KYGBgDQoNCiMjIFRELUFyYWNuZSBQYWlyd2lzZSBhbmQgc2lnUlJlZ3Vsb24NCmBgYHtyfQ0KYXJhY25lU3BsaW5lZFBhaXJ3aXNlU3RhcnRfciA9ICBwcm9jLnRpbWUoKTsNCmFyYWNuZVNwbGluZWRQYWlyd2lzZVJlc3VsdHNfciA9IGxpc3QoKQ0KDQpmb3IoaSBpbiAxOmxlbmd0aChkZnNUb1Rlc3RfcikpIHsNCiAgYXJhY25lU3BsaW5lZFBhaXJ3aXNlUmVzdWx0c19yW1tpXV0gPSBldmFsdWF0ZVREQXJhY25lUGFpcndpc2UoDQogICAgdGl0bGUgPSBwYXN0ZSgiU3BsaW5lXyIsZGZzVG9UZXN0X3JbaV0pLCByb3VuZHMgPSByYW5kb21Sb3VuZHNBcmFjbmUsIA0KICAgIHByb2ZpbGVzUmF3ID0gZ3NlNDQ0MTVfcmF3W3Jvd25hbWVzKGdzZTQ0NDE1X3JhdykgJWluJSBjKHNpZ1JTY28sIHNpZ1JSZWd1bG9uX0thbGxpZmlkYXMpLF0sIA0KICAgIHRpbWUgPSAgZ3NlNDQ0MTVfcmF3X3RpbWUsIHJhd1RpbWUgPSBnc2U0NDQxNV9yYXdfdGltZSwgc3BsaW5lREZzID0gZGZzVG9UZXN0X3JbaV0sDQogICAgcmFuZG9tU2NhbGUgPSBzaWdSUmFuZG9tU2NhbGUsIHJhbmRvbUxlbmd0aCA9IHNpZ1JSYW5kb21MZW5ndGgsZXJyb3JEZWYgPWVycm9yRGVmLCByZWd1bGF0b3JOYW1lID0gc2lnUlNjbywgcmVndWxvbk5hbWVzID0gc2lnUlJlZ3Vsb25fS2FsbGlmaWRhcywgbnVtQmlucyA9IGRlZmF1bHRBcmFjbmVOdW1CaW5zKQ0KfQ0KYXJhY25lU3BsaW5lZFBhaXJ3aXNlRW5kX3IgPSBwcm9jLnRpbWUoKTsNCmFyYWNuZVNwbGluZWRQYWlyd2lzZVRpbWVQZXJSb3VuZF9yID0gKGFyYWNuZVNwbGluZWRQYWlyd2lzZUVuZF9yWyJlbGFwc2VkIl0gLSBhcmFjbmVTcGxpbmVkUGFpcndpc2VTdGFydF9yWyJlbGFwc2VkIl0pIC8gKChyYW5kb21Sb3VuZHNBcmFjbmUgKyAxKSAqIGxlbmd0aChkZnNUb1Rlc3RfcikpOw0KDQoNCmFyYWNuZVJhd1BhaXJ3aXNlU3RhcnRfciA9ICBwcm9jLnRpbWUoKTsNCmFyYWNuZVJhd1BhaXJ3aXNlUmVzdWx0X3IgPSBldmFsdWF0ZVREQXJhY25lUGFpcndpc2UoDQogIHRpdGxlID0gIlJhdyIscm91bmRzID0gcmFuZG9tUm91bmRzQXJhY25lLCANCiAgcHJvZmlsZXNSYXcgPSBnc2U0NDQxNV9yYXdbcm93bmFtZXMoZ3NlNDQ0MTVfcmF3KSAlaW4lIGMoc2lnUlNjbywgc2lnUlJlZ3Vsb25fS2FsbGlmaWRhcyksXSwgDQogIHJhd1RpbWUgPSAgZ3NlNDQ0MTVfcmF3X3RpbWUsIHJhbmRvbVNjYWxlID0gc2lnUlJhbmRvbVNjYWxlLCByYW5kb21MZW5ndGggPSBzaWdSUmFuZG9tTGVuZ3RoLA0KICByZWd1bGF0b3JOYW1lID0gc2lnUlNjbywgcmVndWxvbk5hbWVzID0gc2lnUlJlZ3Vsb25fS2FsbGlmaWRhcywgZXJyb3JEZWYgPSBlcnJvckRlZiwgbnVtQmlucyA9IGRlZmF1bHRBcmFjbmVOdW1CaW5zLA0KICBzcGxpbmVERnMgPSBOVUxMLCB0aW1lID0gTlVMTCkNCmFyYWNuZVJhd1BhaXJ3aXNlRW5kX3IgPSAgcHJvYy50aW1lKCk7DQphcmFjbmVSYXdQYWlyd2lzZVRpbWVQZXJSb3VuZF9yID0gKGFyYWNuZVJhd1BhaXJ3aXNlRW5kX3JbImVsYXBzZWQiXSAtIGFyYWNuZVJhd1BhaXJ3aXNlU3RhcnRfclsiZWxhcHNlZCJdKSAvIChyYW5kb21Sb3VuZHNBcmFjbmUgKyAxKTsNCg0KDQpudW1Db3Jlc1VzZWQgPSBkZXRlY3RDb3JlcygpWzFdIC0gMTsgI1RoZSBzY3JpcHQgbGVhdmVzIG9uZSBDUFUgaWRsZSB0byBsZXQgeW91IHdvcmsgd2hpbGUgaXQgY29tcHV0ZXMNCmBgYA0KDQoNCg0KDQojIyMgUmVzdWx0cyBmb3IgcGFpcndpc2UgVEQtQVJBQ05FIG9uIFNpZ1IgUmVndWxvbiBvZiBLYWxsaWZpZGFzIGV0IGFsLg0KYGBge3J9DQpwcmludFREQXJhY25lRXZhbHVhdGlvbkhlYWRlcigpOw0KI1J1biBvbiByYXcgZGF0YSBpcyBjb21wYXJlZCBhZ2FpbnN0IHRoZSBoaWdoZXN0IGRlZmluaXRpb24gc3BsaW5lDQpwcmludFREQXJhY25lUGFpcndpc2VFdmFsdWF0aW9uKCJOb25lIiwgYXJhY25lUmF3UGFpcndpc2VSZXN1bHRfciwgdmFyaW91c1NwbGluZXNSZXN1bHRfcltbbGVuZ3RoKHZhcmlvdXNTcGxpbmVzUmVzdWx0X3IpXV0pDQpmb3IoaSBpbiAxOmxlbmd0aChkZnNUb1Rlc3RfcikpIHsNCiAgcHJpbnRUREFyYWNuZVBhaXJ3aXNlRXZhbHVhdGlvbihkZnNUb1Rlc3RfcltpXSwgYXJhY25lU3BsaW5lZFBhaXJ3aXNlUmVzdWx0c19yW1tpXV0sIHZhcmlvdXNTcGxpbmVzUmVzdWx0X3JbW2ldXSkNCn0NCmNhdChwYXN0ZTAoIlJhdyB0aW1lIHBlciByb3VuZCAob24gIiwgbnVtQ29yZXNVc2VkLCIgY29yZXMpOiAiLCBhcmFjbmVSYXdQYWlyd2lzZVRpbWVQZXJSb3VuZF9yLCAiIHNlY29uZHNcbiIpKSANCmNhdChwYXN0ZTAoIlNwbGluZWQgdGltZSBwZXIgcm91bmQgKG9uICIsIG51bUNvcmVzVXNlZCwiIGNvcmVzKTogIiwgYXJhY25lU3BsaW5lZFBhaXJ3aXNlVGltZVBlclJvdW5kX3IgLCAiIHNlY29uZHNcbiIpKQ0KDQpgYGANCg0KDQoNCmBgYHtyfQ0KYXJhY25lU3BsaW5lZFBhaXJ3aXNlU3RhcnRfcl9LaW0gPSAgcHJvYy50aW1lKCk7DQphcmFjbmVTcGxpbmVkUGFpcndpc2VSZXN1bHRzX3JfS2ltID0gbGlzdCgpDQoNCmZvcihpIGluIDE6bGVuZ3RoKGRmc1RvVGVzdF9yKSkgew0KICBhcmFjbmVTcGxpbmVkUGFpcndpc2VSZXN1bHRzX3JfS2ltW1tpXV0gPSBldmFsdWF0ZVREQXJhY25lUGFpcndpc2UoDQogICAgdGl0bGUgPSBwYXN0ZSgiU3BsaW5lXyIsZGZzVG9UZXN0X3JbaV0pLCByb3VuZHMgPSByYW5kb21Sb3VuZHNBcmFjbmUsIA0KICAgIHByb2ZpbGVzUmF3ID0gZ3NlNDQ0MTVfcmF3W3Jvd25hbWVzKGdzZTQ0NDE1X3JhdykgJWluJSBjKHNpZ1JTY28sIHNpZ1JSZWd1bG9uX0tpbSksXSwgDQogICAgdGltZSA9ICBnc2U0NDQxNV9yYXdfdGltZSwgcmF3VGltZSA9IGdzZTQ0NDE1X3Jhd190aW1lLCBzcGxpbmVERnMgPSBkZnNUb1Rlc3RfcltpXSwNCiAgICByYW5kb21TY2FsZSA9IHNpZ1JSYW5kb21TY2FsZSwgcmFuZG9tTGVuZ3RoID0gc2lnUlJhbmRvbUxlbmd0aCxlcnJvckRlZiA9ZXJyb3JEZWYsIHJlZ3VsYXRvck5hbWUgPSBzaWdSU2NvLCByZWd1bG9uTmFtZXMgPSBzaWdSUmVndWxvbl9LaW0sIG51bUJpbnMgPSBkZWZhdWx0QXJhY25lTnVtQmlucykNCn0NCmFyYWNuZVNwbGluZWRQYWlyd2lzZUVuZF9yX0tpbSA9IHByb2MudGltZSgpOw0KYXJhY25lU3BsaW5lZFBhaXJ3aXNlVGltZVBlclJvdW5kX3JfS2ltID0gKGFyYWNuZVNwbGluZWRQYWlyd2lzZUVuZF9yX0tpbVsiZWxhcHNlZCJdIC0gYXJhY25lU3BsaW5lZFBhaXJ3aXNlU3RhcnRfcl9LaW1bImVsYXBzZWQiXSkgLyAoKHJhbmRvbVJvdW5kc0FyYWNuZSArIDEpICogbGVuZ3RoKGRmc1RvVGVzdF9yKSk7DQoNCg0KYXJhY25lUmF3UGFpcndpc2VTdGFydF9yX0tpbSA9ICBwcm9jLnRpbWUoKTsNCmFyYWNuZVJhd1BhaXJ3aXNlUmVzdWx0X3JfS2ltID0gZXZhbHVhdGVUREFyYWNuZVBhaXJ3aXNlKA0KICB0aXRsZSA9ICJSYXciLHJvdW5kcyA9IHJhbmRvbVJvdW5kc0FyYWNuZSwgDQogIHByb2ZpbGVzUmF3ID0gZ3NlNDQ0MTVfcmF3W3Jvd25hbWVzKGdzZTQ0NDE1X3JhdykgJWluJSBjKHNpZ1JTY28sIHNpZ1JSZWd1bG9uX0tpbSksXSwgDQogIHJhd1RpbWUgPSAgZ3NlNDQ0MTVfcmF3X3RpbWUsIHJhbmRvbVNjYWxlID0gc2lnUlJhbmRvbVNjYWxlLCByYW5kb21MZW5ndGggPSBzaWdSUmFuZG9tTGVuZ3RoLA0KICByZWd1bGF0b3JOYW1lID0gc2lnUlNjbywgcmVndWxvbk5hbWVzID0gc2lnUlJlZ3Vsb25fS2ltLCBlcnJvckRlZiA9IGVycm9yRGVmLCBudW1CaW5zID0gZGVmYXVsdEFyYWNuZU51bUJpbnMsDQogIHNwbGluZURGcyA9IE5VTEwsIHRpbWUgPSBOVUxMKQ0KYXJhY25lUmF3UGFpcndpc2VFbmRfcl9LaW0gPSAgcHJvYy50aW1lKCk7DQphcmFjbmVSYXdQYWlyd2lzZVRpbWVQZXJSb3VuZF9yX0tpbSA9IChhcmFjbmVSYXdQYWlyd2lzZUVuZF9yX0tpbVsiZWxhcHNlZCJdIC0gYXJhY25lUmF3UGFpcndpc2VTdGFydF9yX0tpbVsiZWxhcHNlZCJdKSAvIChyYW5kb21Sb3VuZHNBcmFjbmUgKyAxKTsNCg0KDQpudW1Db3Jlc1VzZWQgPSBkZXRlY3RDb3JlcygpWzFdIC0gMTsgI1RoZSBzY3JpcHQgbGVhdmVzIG9uZSBDUFUgaWRsZSB0byBsZXQgeW91IHdvcmsgd2hpbGUgaXQgY29tcHV0ZXMNCg0KYGBgDQoNCiMjIyBSZXN1bHRzIGZvciBwYWlyd2lzZSBURC1BUkFDTkUgLSBLaW0gZXQgYWwuDQpgYGB7cn0NCiNSdW4gb24gcmF3IGRhdGEgaXMgY29tcGFyZWQgYWdhaW5zdCB0aGUgaGlnaGVzdCBkZWZpbml0aW9uIHNwbGluZQ0KcHJpbnRUREFyYWNuZUV2YWx1YXRpb25IZWFkZXIoKTsNCnByaW50VERBcmFjbmVQYWlyd2lzZUV2YWx1YXRpb24oIk5vbmUiLCBhcmFjbmVSYXdQYWlyd2lzZVJlc3VsdF9yX0tpbSwgdmFyaW91c1NwbGluZXNSZXN1bHRfcl9raW1bW2xlbmd0aCh2YXJpb3VzU3BsaW5lc1Jlc3VsdF9yX2tpbSldXSkNCmZvcihpIGluIDE6bGVuZ3RoKGRmc1RvVGVzdF9yKSkgew0KICBwcmludFREQXJhY25lUGFpcndpc2VFdmFsdWF0aW9uKGRmc1RvVGVzdF9yW2ldLCBhcmFjbmVTcGxpbmVkUGFpcndpc2VSZXN1bHRzX3JfS2ltW1tpXV0sIHZhcmlvdXNTcGxpbmVzUmVzdWx0X3Jfa2ltW1tpXV0pDQp9DQpjYXQocGFzdGUwKCJSYXcgdGltZSBwZXIgcm91bmQgKG9uICIsIG51bUNvcmVzVXNlZCwiIGNvcmVzKTogIiwgYXJhY25lUmF3UGFpcndpc2VUaW1lUGVyUm91bmRfcl9LaW0sICIgc2Vjb25kc1xuIikpIA0KY2F0KHBhc3RlMCgiU3BsaW5lZCB0aW1lIHBlciByb3VuZCAob24gIiwgbnVtQ29yZXNVc2VkLCIgY29yZXMpOiAiLCBhcmFjbmVTcGxpbmVkUGFpcndpc2VUaW1lUGVyUm91bmRfcl9LaW0gLCAiIHNlY29uZHNcbiIpKQ0KDQpgYGANCg0KYGBge3J9DQoNCg0KYXJhY25lU3BsaW5lZFBhaXJ3aXNlU3RhcnRfcl9LaW1fU3Ryb25nID0gIHByb2MudGltZSgpOw0KYXJhY25lU3BsaW5lZFBhaXJ3aXNlUmVzdWx0c19yX0tpbV9TdHJvbmcgPSBsaXN0KCkNCg0KZm9yKGkgaW4gMTpsZW5ndGgoZGZzVG9UZXN0X3IpKSB7DQogIGFyYWNuZVNwbGluZWRQYWlyd2lzZVJlc3VsdHNfcl9LaW1fU3Ryb25nW1tpXV0gPSBldmFsdWF0ZVREQXJhY25lUGFpcndpc2UoDQogICAgdGl0bGUgPSBwYXN0ZSgiU3BsaW5lXyIsZGZzVG9UZXN0X3JbaV0pLCByb3VuZHMgPSByYW5kb21Sb3VuZHNBcmFjbmUsIA0KICAgIHByb2ZpbGVzUmF3ID0gZ3NlNDQ0MTVfcmF3W3Jvd25hbWVzKGdzZTQ0NDE1X3JhdykgJWluJSBjKHNpZ1JTY28sIHNpZ1JSZWd1bG9uX0tpbV9TdHJvbmcpLF0sIA0KICAgIHRpbWUgPSAgZ3NlNDQ0MTVfcmF3X3RpbWUsIHJhd1RpbWUgPSBnc2U0NDQxNV9yYXdfdGltZSwgc3BsaW5lREZzID0gZGZzVG9UZXN0X3JbaV0sDQogICAgcmFuZG9tU2NhbGUgPSBzaWdSUmFuZG9tU2NhbGUsIHJhbmRvbUxlbmd0aCA9IHNpZ1JSYW5kb21MZW5ndGgsZXJyb3JEZWYgPWVycm9yRGVmLCByZWd1bGF0b3JOYW1lID0gc2lnUlNjbywgcmVndWxvbk5hbWVzID0gc2lnUlJlZ3Vsb25fS2ltX1N0cm9uZywgbnVtQmlucyA9IGRlZmF1bHRBcmFjbmVOdW1CaW5zKQ0KfQ0KYXJhY25lU3BsaW5lZFBhaXJ3aXNlRW5kX3JfS2ltX1N0cm9uZyA9IHByb2MudGltZSgpOw0KYXJhY25lU3BsaW5lZFBhaXJ3aXNlVGltZVBlclJvdW5kX3JfS2ltX1N0cm9uZyA9IChhcmFjbmVTcGxpbmVkUGFpcndpc2VFbmRfcl9LaW1fU3Ryb25nWyJlbGFwc2VkIl0gLSBhcmFjbmVTcGxpbmVkUGFpcndpc2VTdGFydF9yX0tpbV9TdHJvbmdbImVsYXBzZWQiXSkgLyAoKHJhbmRvbVJvdW5kc0FyYWNuZSArIDEpICogbGVuZ3RoKGRmc1RvVGVzdF9yKSk7DQoNCg0KYXJhY25lUmF3UGFpcndpc2VTdGFydF9yX0tpbV9TdHJvbmcgPSAgcHJvYy50aW1lKCk7DQphcmFjbmVSYXdQYWlyd2lzZVJlc3VsdF9yX0tpbV9TdHJvbmcgPSBldmFsdWF0ZVREQXJhY25lUGFpcndpc2UoDQogIHRpdGxlID0gIlJhdyIscm91bmRzID0gcmFuZG9tUm91bmRzQXJhY25lLCANCiAgcHJvZmlsZXNSYXcgPSBnc2U0NDQxNV9yYXdbcm93bmFtZXMoZ3NlNDQ0MTVfcmF3KSAlaW4lIGMoc2lnUlNjbywgc2lnUlJlZ3Vsb25fS2ltX1N0cm9uZyksXSwgDQogIHJhd1RpbWUgPSAgZ3NlNDQ0MTVfcmF3X3RpbWUsIHJhbmRvbVNjYWxlID0gc2lnUlJhbmRvbVNjYWxlLCByYW5kb21MZW5ndGggPSBzaWdSUmFuZG9tTGVuZ3RoLA0KICByZWd1bGF0b3JOYW1lID0gc2lnUlNjbywgcmVndWxvbk5hbWVzID0gc2lnUlJlZ3Vsb25fS2ltX1N0cm9uZywgZXJyb3JEZWYgPSBlcnJvckRlZiwgbnVtQmlucyA9IGRlZmF1bHRBcmFjbmVOdW1CaW5zLA0KICBzcGxpbmVERnMgPSBOVUxMLCB0aW1lID0gTlVMTCkNCmFyYWNuZVJhd1BhaXJ3aXNlRW5kX3JfS2ltX1N0cm9uZyA9ICBwcm9jLnRpbWUoKTsNCmFyYWNuZVJhd1BhaXJ3aXNlVGltZVBlclJvdW5kX3JfS2ltX1N0cm9uZyA9IChhcmFjbmVSYXdQYWlyd2lzZUVuZF9yX0tpbV9TdHJvbmdbImVsYXBzZWQiXSAtIGFyYWNuZVJhd1BhaXJ3aXNlU3RhcnRfcl9LaW1fU3Ryb25nWyJlbGFwc2VkIl0pIC8gKHJhbmRvbVJvdW5kc0FyYWNuZSArIDEpOw0KDQoNCm51bUNvcmVzVXNlZCA9IGRldGVjdENvcmVzKClbMV0gLSAxOyAjVGhlIHNjcmlwdCBsZWF2ZXMgb25lIENQVSBpZGxlIHRvIGxldCB5b3Ugd29yayB3aGlsZSBpdCBjb21wdXRlcw0KDQpgYGANCg0KIyMjIFJlc3VsdHMgZm9yIHBhaXJ3aXNlIFRELUFSQUNORSBLaW0gZXQgYWwuIFN0cm9uZw0KYGBge3J9DQojUnVuIG9uIHJhdyBkYXRhIGlzIGNvbXBhcmVkIGFnYWluc3QgdGhlIGhpZ2hlc3QgZGVmaW5pdGlvbiBzcGxpbmUNCnByaW50VERBcmFjbmVFdmFsdWF0aW9uSGVhZGVyKCk7DQpwcmludFREQXJhY25lUGFpcndpc2VFdmFsdWF0aW9uKCJOb25lIiwgYXJhY25lUmF3UGFpcndpc2VSZXN1bHRfcl9LaW1fU3Ryb25nLCB2YXJpb3VzU3BsaW5lc1Jlc3VsdF9yX2tpbV9zdHJvbmdbW2xlbmd0aCh2YXJpb3VzU3BsaW5lc1Jlc3VsdF9yX2tpbV9zdHJvbmcpXV0pDQpmb3IoaSBpbiAxOmxlbmd0aChkZnNUb1Rlc3RfcikpIHsNCiAgcHJpbnRUREFyYWNuZVBhaXJ3aXNlRXZhbHVhdGlvbihwYXN0ZTAoZGZzVG9UZXN0X3JbaV0pLCBhcmFjbmVTcGxpbmVkUGFpcndpc2VSZXN1bHRzX3JfS2ltX1N0cm9uZ1tbaV1dLCB2YXJpb3VzU3BsaW5lc1Jlc3VsdF9yX2tpbV9zdHJvbmdbW2ldXSkNCn0NCmNhdChwYXN0ZTAoIlJhdyB0aW1lIHBlciByb3VuZCAob24gIiwgbnVtQ29yZXNVc2VkLCIgY29yZXMpOiAiLCBhcmFjbmVSYXdQYWlyd2lzZVRpbWVQZXJSb3VuZF9yX0tpbV9TdHJvbmcsICIgc2Vjb25kc1xuIikpIA0KY2F0KHBhc3RlMCgiU3BsaW5lZCB0aW1lIHBlciByb3VuZCAob24gIiwgbnVtQ29yZXNVc2VkLCIgY29yZXMpOiAiLCBhcmFjbmVTcGxpbmVkUGFpcndpc2VUaW1lUGVyUm91bmRfcl9LaW1fU3Ryb25nICwgIiBzZWNvbmRzXG4iKSkNCg0KYGBgDQoNCg0K
