# Supplementary material for: Genexpi: a toolset for identifying regulons and validating gene regulatory networks using time-course expression data
Source: BMC Bioinformatics. 2018 Apr 13;19:137. doi: 10.1186/s12859-018-2138-x (PMC5899412; doi:10.1186/s12859-018-2138-x)
Supplement: Supplementary file 1 — evaluation.zip - an archive containing: • evaluation.Rmd – R Markdown notebook (best used with RStudio, https://www.rstudio.com/) to reproduce the evaluation on bacterial regulons in this paper. evaluation.nb.html – Compiled version of evaluation.Rmd for easy reading, including stored results produced by running all the code. • evaluation_sacharomyces.Rmd – R Markdown notebook to reproduce the evaluation on Sacharomyces data. • evaluation_sacharomyces.nb.html – Compiled version of evaluation_sacharomyces.Rmd, including stored results produced by running all the code. [file 12859_2018_2138_MOESM1_ESM.zip › evaluation_sacharomyces.nb.html]

Evaluating Sacharomyces cell cycle genes


Code 

- Show All Code
- Hide All Code
- Download Rmd

# Evaluating Sacharomyces cell cycle genes

# Setting up


```
  options(java.parameters = "-Xmx2048m")
```


The code below should install all required packages for the evaluation.


```
  # Helper function to install packages if they are not available
  pkgTest <- function(x)
  {
    if (!require(x,character.only = TRUE))
    {
      install.packages(x,dep=TRUE)
        if(!require(x,character.only = TRUE)) stop("Package not found")
    }
  }

  source("https://bioconductor.org/biocLite.R")
  biocLite(c("Biobase","TDARACNE", "RBGL"))

  pkgTest("splines")
  pkgTest("foreach")
  pkgTest("doParallel")
  pkgTest("rJava")
  pkgTest("tidyverse")
  
  pkgTest("devtools")
  install_github("cas-bioinf/genexpi", ref = "bmc-release", subdir="rpackage")
```


Load the packages


```
  library(Biobase)
  library(TDARACNE)
  library(RBGL)
  
  library(splines)
  library(foreach)
  library(doParallel)
  library(rJava)
  library(tidyverse)
  library(rGenexpi)
```


## Download the data from Yeastract and GEO datasets


```
yeastract_file <- 'yeastract.tsv.gz'
if(!file.exists(yeastract_file)) {
  download.file("http://www.yeastract.com/download/RegulationTwoColumnTable_Documented_2013927.tsv.gz", yeastract_file)
}
```


```
trying URL 'http://www.yeastract.com/download/RegulationTwoColumnTable_Documented_2013927.tsv.gz'
Content type 'application/x-gzip' length 714492 bytes (697 KB)
downloaded 697 KB
```


```
yeastract_df <- read.delim(gzfile(yeastract_file),sep = ";",header = FALSE, col.names = c("regulator","target"))
```


```
gds38_file <- 'gds38.soft.gz';
if(!file.exists(gds38_file)) {
  download.file("ftp://ftp.ncbi.nlm.nih.gov/geo/datasets/GDSnnn/GDS38/soft/GDS38_full.soft.gz", gds38_file)
}
```


```
trying URL 'ftp://ftp.ncbi.nlm.nih.gov/geo/datasets/GDSnnn/GDS38/soft/GDS38_full.soft.gz'
downloaded 1.2 MB
```


```
#Intermediate data frame representation
gds38_raw_df = read.delim(gzfile(gds38_file), skip = 149, comment.char = "!", na.strings = c("null",""))
```

## Data processing


```
#SWI4/6, MBP1 a re suspect as they act in complexes (BTW good test case for the cooperative model)
regulators <- c("FKH1", "FKH2","MCM1","MBP1","SWI4","SWI6","NDD1", "ACE2")
yeastract_filtered <- yeastract_df%>% filter(regulator %in% regulators) %>%
  filter(target %in% gds38_raw_df$IDENTIFIER) %>%
  group_by(regulator)
all_targets <- as.character(yeastract_filtered$target)
genes_of_interest <- unique(as.character(c(regulators, all_targets)))
gds38_raw <- aggregate(gds38_raw_df[,3:18], gds38_raw_df["IDENTIFIER"], mean, na.rm = TRUE) %>%
  column_to_rownames("IDENTIFIER") %>%
  as.matrix() %>% exp()
  
gds38_raw_filtered <- gds38_raw[genes_of_interest,]
time_raw <- seq(0,by = 7, length.out = 16)
time_smooth <- 0:max(time_raw)
```

## Splining the profiles + inspecting results


```
splineDF = 6
gds38_smooth_filtered <- splineProfileMatrix(gds38_raw_filtered, time_raw, time_smooth, df = splineDF, intercept = TRUE)
```


```
Some values in profileMatrix are NA, computation of splines will be slower
```


```
inspectSmoothing(time_raw, gds38_raw_filtered,time_smooth, gds38_smooth_filtered, c("ACE2","FKH2","SWI4"))
```


```
ACE2 - orange 
FKH2 - blue 
SWI4 - green
```

# The evaluation itself


```
errorDef <- defaultErrorDef()
errorDef$relative <- 0.1
errorDef$minimal <- 0.2
deviceSpecs <- getDeviceSpecs(deviceType = "processor")
minFitQuality <- 0.8
randomRounds <- 20
```


## Filter out constant synthesis


```
regulators_logical <- rownames(gds38_smooth_filtered) %in% regulators 
constantProfiles = testConstant(gds38_smooth_filtered, errorDef)
profilesToTestConstantSynthesisIndices = which(!constantProfiles & !regulators_logical)

constantSynthesisResults = computeConstantSynthesis(deviceSpecs, gds38_smooth_filtered, tasks = profilesToTestConstantSynthesisIndices);

constantSynthesisProfiles = testConstantSynthesis(constantSynthesisResults, errorDef, minFitQuality);

profilesToTest_logical <- regulators_logical | (!constantProfiles & !constantSynthesisProfiles)

gds38_raw_to_test <- gds38_raw_filtered[profilesToTest_logical,]
gds38_smooth_to_test <- gds38_smooth_filtered[profilesToTest_logical,]

targets_to_test <- rownames(gds38_smooth_filtered)[(!constantProfiles & !constantSynthesisProfiles)]
```

## Test random profile generation

Here the dots are actual measured profiles of several regulators, while the lines are randomly generated profiles.


```
randomScale = 1.6
randomLength = 10
#See ACE2
plotRandomProfiles(20, time_raw, scale = randomScale, length = randomLength,trueTime = time_raw,  trueProfile = gds38_raw["ACE2",])
```


```
#See FKH1
plotRandomProfiles(20, time_raw, scale = randomScale, length = randomLength,trueTime = time_raw,  trueProfile = gds38_raw["FKH1",])
```


```
#See SWI4
plotRandomProfiles(20, time_raw, scale = randomScale, length =                randomLength,trueTime = time_raw,  trueProfile = gds38_raw["SWI4",])
```

## Subsample the regulons


```
results_genexpi <- list()
results_aracne <- list()
set.seed(21345678)
regulons_to_test = list()
for(regulator_to_test in regulators) {
  regulon_to_test_df <- yeastract_filtered %>% filter(regulator == regulator_to_test & target %in% targets_to_test)
  if(nrow(regulon_to_test_df) > 30) {
    regulon_to_test_df <- regulon_to_test_df %>% sample_n(30)
  }
  regulons_to_test[[regulator_to_test]] <- regulon_to_test_df$target %>% as.character()
}
```

## Execute Genexpi and TD-Aracne


```
for(regulator_to_test in regulators) {
  cat("======", regulator_to_test, "=====\n")
  regulon_to_test <- regulons_to_test[[regulator_to_test]]
  
  #This is tu support restarts of the computation
  if(is.null(results_genexpi[[regulator_to_test]])) {
    evaluation_res <- evaluateRandomForRegulon(deviceSpecs = deviceSpecs,rawProfiles = gds38_raw_to_test, rounds = randomRounds, regulatorName = regulator_to_test, regulonNames = regulon_to_test, checkConstantSynthesis = FALSE, time = time_smooth, rawTime = time_raw, randomScale = randomScale, randomLength = randomLength, splineDFs = splineDF, splineIntercept = TRUE, errorDef = errorDef)
    results_genexpi[[regulator_to_test]] <- evaluation_res
    save.image("evaluation_sacharomyces.RData")
  }
}
for(regulator_to_test in regulators) {
  cat("====== Aracne:", regulator_to_test, "=====\n")
  regulon_to_test <- regulons_to_test[[regulator_to_test]]
  #This is tu support restarts of the computation
  if(is.null(results_aracne[[regulator_to_test]])) {
    res_aracne <- evaluateTDAracne(randomRounds, gds38_raw, time_raw, c(splineDF), time_smooth, randomScale, randomLength, regulatorName = regulator_to_test, regulonNames = regulon_to_test, errorDef = errorDef, numBins = 10)
    results_aracne[[regulator_to_test]] <- res_aracne
    save.image("evaluation_sacharomyces.RData")
  }
  
}
```

## Output the results

For the publication, we chose the better result for TD-Aracne (either downstream or connected) separately for each regulator.


```
results_formatted <- list()
step = 1
for(regulator in regulators) {
  if(!is.null(results_genexpi[[regulator]])) {
    res_genexpi <- results_genexpi[[regulator]]
    results_formatted[[step]] <- data.frame(regulator = regulator, type = "Genexpi", true = res_genexpi$trueRatio, random = mean(res_genexpi$randomRatios))
    step <- step + 1
  }
  
  if(!is.null(results_aracne[[regulator]])) {
    res_aracne <- results_aracne[[regulator]]
    results_formatted[[step]] <- data.frame(regulator = regulator, type = "TD-Aracne-Downstream", true = res_aracne$trueRatioDownstream, random = res_aracne$overallRandomRatioDownstream)
    step <- step + 1
    results_formatted[[step]] <- data.frame(regulator = regulator, type = "TD-Aracne-Connected", true = res_aracne$trueRatioConnected, random = res_aracne$overallRandomRatioConnected)
    step <- step + 1
  }
}
results_formatted <- do.call(rbind, results_formatted)
results_formatted
```

LS0tDQp0aXRsZTogIkV2YWx1YXRpbmcgU2FjaGFyb215Y2VzIGNlbGwgY3ljbGUgZ2VuZXMiDQpvdXRwdXQ6IGh0bWxfbm90ZWJvb2sNCi0tLQ0KIyBTZXR0aW5nIHVwDQoNCmBgYHtyIHNldHVwfQ0KICBvcHRpb25zKGphdmEucGFyYW1ldGVycyA9ICItWG14MjA0OG0iKQ0KYGBgDQoNClRoZSBjb2RlIGJlbG93IHNob3VsZCBpbnN0YWxsIGFsbCByZXF1aXJlZCBwYWNrYWdlcyBmb3IgdGhlIGV2YWx1YXRpb24uDQoNCmBgYHtyIGV2YWw9RkFMU0V9DQogICMgSGVscGVyIGZ1bmN0aW9uIHRvIGluc3RhbGwgcGFja2FnZXMgaWYgdGhleSBhcmUgbm90IGF2YWlsYWJsZQ0KICBwa2dUZXN0IDwtIGZ1bmN0aW9uKHgpDQogIHsNCiAgICBpZiAoIXJlcXVpcmUoeCxjaGFyYWN0ZXIub25seSA9IFRSVUUpKQ0KICAgIHsNCiAgICAgIGluc3RhbGwucGFja2FnZXMoeCxkZXA9VFJVRSkNCiAgICAgICAgaWYoIXJlcXVpcmUoeCxjaGFyYWN0ZXIub25seSA9IFRSVUUpKSBzdG9wKCJQYWNrYWdlIG5vdCBmb3VuZCIpDQogICAgfQ0KICB9DQoNCiAgc291cmNlKCJodHRwczovL2Jpb2NvbmR1Y3Rvci5vcmcvYmlvY0xpdGUuUiIpDQogIGJpb2NMaXRlKGMoIkJpb2Jhc2UiLCJUREFSQUNORSIsICJSQkdMIikpDQoNCiAgcGtnVGVzdCgic3BsaW5lcyIpDQogIHBrZ1Rlc3QoImZvcmVhY2giKQ0KICBwa2dUZXN0KCJkb1BhcmFsbGVsIikNCiAgcGtnVGVzdCgickphdmEiKQ0KICBwa2dUZXN0KCJ0aWR5dmVyc2UiKQ0KICANCiAgcGtnVGVzdCgiZGV2dG9vbHMiKQ0KICBpbnN0YWxsX2dpdGh1YigiY2FzLWJpb2luZi9nZW5leHBpIiwgcmVmID0gImJtYy1yZWxlYXNlIiwgc3ViZGlyPSJycGFja2FnZSIpDQoNCmBgYA0KDQpMb2FkIHRoZSBwYWNrYWdlcw0KDQpgYGB7ciByZXN1bHRzPSdoaWRlJ30NCiAgbGlicmFyeShCaW9iYXNlKQ0KICBsaWJyYXJ5KFREQVJBQ05FKQ0KICBsaWJyYXJ5KFJCR0wpDQogIA0KICBsaWJyYXJ5KHNwbGluZXMpDQogIGxpYnJhcnkoZm9yZWFjaCkNCiAgbGlicmFyeShkb1BhcmFsbGVsKQ0KICBsaWJyYXJ5KHJKYXZhKQ0KICBsaWJyYXJ5KHRpZHl2ZXJzZSkNCiAgbGlicmFyeShyR2VuZXhwaSkNCmBgYA0KDQojIyBEb3dubG9hZCB0aGUgZGF0YSBmcm9tIFllYXN0cmFjdCBhbmQgR0VPIGRhdGFzZXRzDQoNCmBgYHtyfQ0KeWVhc3RyYWN0X2ZpbGUgPC0gJ3llYXN0cmFjdC50c3YuZ3onDQppZighZmlsZS5leGlzdHMoeWVhc3RyYWN0X2ZpbGUpKSB7DQogIGRvd25sb2FkLmZpbGUoImh0dHA6Ly93d3cueWVhc3RyYWN0LmNvbS9kb3dubG9hZC9SZWd1bGF0aW9uVHdvQ29sdW1uVGFibGVfRG9jdW1lbnRlZF8yMDEzOTI3LnRzdi5neiIsIHllYXN0cmFjdF9maWxlKQ0KfQ0KDQp5ZWFzdHJhY3RfZGYgPC0gcmVhZC5kZWxpbShnemZpbGUoeWVhc3RyYWN0X2ZpbGUpLHNlcCA9ICI7IixoZWFkZXIgPSBGQUxTRSwgY29sLm5hbWVzID0gYygicmVndWxhdG9yIiwidGFyZ2V0IikpDQpgYGANCg0KDQoNCmBgYHtyfQ0KZ2RzMzhfZmlsZSA8LSAnZ2RzMzguc29mdC5neic7DQppZighZmlsZS5leGlzdHMoZ2RzMzhfZmlsZSkpIHsNCiAgZG93bmxvYWQuZmlsZSgiZnRwOi8vZnRwLm5jYmkubmxtLm5paC5nb3YvZ2VvL2RhdGFzZXRzL0dEU25ubi9HRFMzOC9zb2Z0L0dEUzM4X2Z1bGwuc29mdC5neiIsIGdkczM4X2ZpbGUpDQp9DQoNCiNJbnRlcm1lZGlhdGUgZGF0YSBmcmFtZSByZXByZXNlbnRhdGlvbg0KZ2RzMzhfcmF3X2RmID0gcmVhZC5kZWxpbShnemZpbGUoZ2RzMzhfZmlsZSksIHNraXAgPSAxNDksIGNvbW1lbnQuY2hhciA9ICIhIiwgbmEuc3RyaW5ncyA9IGMoIm51bGwiLCIiKSkgDQoNCmBgYA0KDQojIyBEYXRhIHByb2Nlc3NpbmcNCg0KYGBge3J9DQojU1dJNC82LCBNQlAxIGEgcmUgc3VzcGVjdCBhcyB0aGV5IGFjdCBpbiBjb21wbGV4ZXMgKEJUVyBnb29kIHRlc3QgY2FzZSBmb3IgdGhlIGNvb3BlcmF0aXZlIG1vZGVsKQ0KDQpyZWd1bGF0b3JzIDwtIGMoIkZLSDEiLCAiRktIMiIsIk1DTTEiLCJNQlAxIiwiU1dJNCIsIlNXSTYiLCJOREQxIiwgIkFDRTIiKQ0KeWVhc3RyYWN0X2ZpbHRlcmVkIDwtIHllYXN0cmFjdF9kZiU+JSBmaWx0ZXIocmVndWxhdG9yICVpbiUgcmVndWxhdG9ycykgJT4lDQogIGZpbHRlcih0YXJnZXQgJWluJSBnZHMzOF9yYXdfZGYkSURFTlRJRklFUikgJT4lDQogIGdyb3VwX2J5KHJlZ3VsYXRvcikNCg0KYWxsX3RhcmdldHMgPC0gYXMuY2hhcmFjdGVyKHllYXN0cmFjdF9maWx0ZXJlZCR0YXJnZXQpDQpnZW5lc19vZl9pbnRlcmVzdCA8LSB1bmlxdWUoYXMuY2hhcmFjdGVyKGMocmVndWxhdG9ycywgYWxsX3RhcmdldHMpKSkNCg0KZ2RzMzhfcmF3IDwtIGFnZ3JlZ2F0ZShnZHMzOF9yYXdfZGZbLDM6MThdLCBnZHMzOF9yYXdfZGZbIklERU5USUZJRVIiXSwgbWVhbiwgbmEucm0gPSBUUlVFKSAlPiUNCiAgY29sdW1uX3RvX3Jvd25hbWVzKCJJREVOVElGSUVSIikgJT4lDQogIGFzLm1hdHJpeCgpICU+JSBleHAoKQ0KICANCmdkczM4X3Jhd19maWx0ZXJlZCA8LSBnZHMzOF9yYXdbZ2VuZXNfb2ZfaW50ZXJlc3QsXQ0KDQp0aW1lX3JhdyA8LSBzZXEoMCxieSA9IDcsIGxlbmd0aC5vdXQgPSAxNikNCnRpbWVfc21vb3RoIDwtIDA6bWF4KHRpbWVfcmF3KQ0KYGBgDQoNCiMjIFNwbGluaW5nIHRoZSBwcm9maWxlcyArIGluc3BlY3RpbmcgcmVzdWx0cw0KYGBge3J9DQpzcGxpbmVERiA9IDYNCmdkczM4X3Ntb290aF9maWx0ZXJlZCA8LSBzcGxpbmVQcm9maWxlTWF0cml4KGdkczM4X3Jhd19maWx0ZXJlZCwgdGltZV9yYXcsIHRpbWVfc21vb3RoLCBkZiA9IHNwbGluZURGLCBpbnRlcmNlcHQgPSBUUlVFKQ0KaW5zcGVjdFNtb290aGluZyh0aW1lX3JhdywgZ2RzMzhfcmF3X2ZpbHRlcmVkLHRpbWVfc21vb3RoLCBnZHMzOF9zbW9vdGhfZmlsdGVyZWQsIGMoIkFDRTIiLCJGS0gyIiwiU1dJNCIpKQ0KDQpgYGANCiMgVGhlIGV2YWx1YXRpb24gaXRzZWxmDQoNCmBgYHtyfQ0KZXJyb3JEZWYgPC0gZGVmYXVsdEVycm9yRGVmKCkNCmVycm9yRGVmJHJlbGF0aXZlIDwtIDAuMQ0KZXJyb3JEZWYkbWluaW1hbCA8LSAwLjINCmRldmljZVNwZWNzIDwtIGdldERldmljZVNwZWNzKGRldmljZVR5cGUgPSAicHJvY2Vzc29yIikNCm1pbkZpdFF1YWxpdHkgPC0gMC44DQpyYW5kb21Sb3VuZHMgPC0gMjANCmBgYA0KDQojIyBGaWx0ZXIgb3V0IGNvbnN0YW50IHN5bnRoZXNpcw0KYGBge3J9DQpyZWd1bGF0b3JzX2xvZ2ljYWwgPC0gcm93bmFtZXMoZ2RzMzhfc21vb3RoX2ZpbHRlcmVkKSAlaW4lIHJlZ3VsYXRvcnMgDQpjb25zdGFudFByb2ZpbGVzID0gdGVzdENvbnN0YW50KGdkczM4X3Ntb290aF9maWx0ZXJlZCwgZXJyb3JEZWYpDQpwcm9maWxlc1RvVGVzdENvbnN0YW50U3ludGhlc2lzSW5kaWNlcyA9IHdoaWNoKCFjb25zdGFudFByb2ZpbGVzICYgIXJlZ3VsYXRvcnNfbG9naWNhbCkNCg0KY29uc3RhbnRTeW50aGVzaXNSZXN1bHRzID0gY29tcHV0ZUNvbnN0YW50U3ludGhlc2lzKGRldmljZVNwZWNzLCBnZHMzOF9zbW9vdGhfZmlsdGVyZWQsIHRhc2tzID0gcHJvZmlsZXNUb1Rlc3RDb25zdGFudFN5bnRoZXNpc0luZGljZXMpOw0KDQpjb25zdGFudFN5bnRoZXNpc1Byb2ZpbGVzID0gdGVzdENvbnN0YW50U3ludGhlc2lzKGNvbnN0YW50U3ludGhlc2lzUmVzdWx0cywgZXJyb3JEZWYsIG1pbkZpdFF1YWxpdHkpOw0KDQpwcm9maWxlc1RvVGVzdF9sb2dpY2FsIDwtIHJlZ3VsYXRvcnNfbG9naWNhbCB8ICghY29uc3RhbnRQcm9maWxlcyAmICFjb25zdGFudFN5bnRoZXNpc1Byb2ZpbGVzKQ0KDQpnZHMzOF9yYXdfdG9fdGVzdCA8LSBnZHMzOF9yYXdfZmlsdGVyZWRbcHJvZmlsZXNUb1Rlc3RfbG9naWNhbCxdDQpnZHMzOF9zbW9vdGhfdG9fdGVzdCA8LSBnZHMzOF9zbW9vdGhfZmlsdGVyZWRbcHJvZmlsZXNUb1Rlc3RfbG9naWNhbCxdDQoNCnRhcmdldHNfdG9fdGVzdCA8LSByb3duYW1lcyhnZHMzOF9zbW9vdGhfZmlsdGVyZWQpWyghY29uc3RhbnRQcm9maWxlcyAmICFjb25zdGFudFN5bnRoZXNpc1Byb2ZpbGVzKV0NCmBgYA0KDQojIyBUZXN0IHJhbmRvbSBwcm9maWxlIGdlbmVyYXRpb24NCg0KSGVyZSB0aGUgZG90cyBhcmUgYWN0dWFsIG1lYXN1cmVkIHByb2ZpbGVzIG9mIHNldmVyYWwgcmVndWxhdG9ycywgd2hpbGUgdGhlIGxpbmVzIGFyZSByYW5kb21seSBnZW5lcmF0ZWQgcHJvZmlsZXMuDQoNCmBgYHtyfQ0KcmFuZG9tU2NhbGUgPSAxLjYNCnJhbmRvbUxlbmd0aCA9IDEwDQojU2VlIEFDRTINCnBsb3RSYW5kb21Qcm9maWxlcygyMCwgdGltZV9yYXcsIHNjYWxlID0gcmFuZG9tU2NhbGUsIGxlbmd0aCA9IHJhbmRvbUxlbmd0aCx0cnVlVGltZSA9IHRpbWVfcmF3LCAgdHJ1ZVByb2ZpbGUgPSBnZHMzOF9yYXdbIkFDRTIiLF0pDQojU2VlIEZLSDENCnBsb3RSYW5kb21Qcm9maWxlcygyMCwgdGltZV9yYXcsIHNjYWxlID0gcmFuZG9tU2NhbGUsIGxlbmd0aCA9IHJhbmRvbUxlbmd0aCx0cnVlVGltZSA9IHRpbWVfcmF3LCAgdHJ1ZVByb2ZpbGUgPSBnZHMzOF9yYXdbIkZLSDEiLF0pDQojU2VlIFNXSTQNCnBsb3RSYW5kb21Qcm9maWxlcygyMCwgdGltZV9yYXcsIHNjYWxlID0gcmFuZG9tU2NhbGUsIGxlbmd0aCA9ICAgICAgICAgICAgICAgIHJhbmRvbUxlbmd0aCx0cnVlVGltZSA9IHRpbWVfcmF3LCAgdHJ1ZVByb2ZpbGUgPSBnZHMzOF9yYXdbIlNXSTQiLF0pDQpgYGANCg0KIyMgU3Vic2FtcGxlIHRoZSByZWd1bG9ucw0KDQpgYGB7cn0NCnJlc3VsdHNfZ2VuZXhwaSA8LSBsaXN0KCkNCnJlc3VsdHNfYXJhY25lIDwtIGxpc3QoKQ0KDQpzZXQuc2VlZCgyMTM0NTY3OCkNCnJlZ3Vsb25zX3RvX3Rlc3QgPSBsaXN0KCkNCmZvcihyZWd1bGF0b3JfdG9fdGVzdCBpbiByZWd1bGF0b3JzKSB7DQogIHJlZ3Vsb25fdG9fdGVzdF9kZiA8LSB5ZWFzdHJhY3RfZmlsdGVyZWQgJT4lIGZpbHRlcihyZWd1bGF0b3IgPT0gcmVndWxhdG9yX3RvX3Rlc3QgJiB0YXJnZXQgJWluJSB0YXJnZXRzX3RvX3Rlc3QpDQogIGlmKG5yb3cocmVndWxvbl90b190ZXN0X2RmKSA+IDMwKSB7DQogICAgcmVndWxvbl90b190ZXN0X2RmIDwtIHJlZ3Vsb25fdG9fdGVzdF9kZiAlPiUgc2FtcGxlX24oMzApDQogIH0NCiAgcmVndWxvbnNfdG9fdGVzdFtbcmVndWxhdG9yX3RvX3Rlc3RdXSA8LSByZWd1bG9uX3RvX3Rlc3RfZGYkdGFyZ2V0ICU+JSBhcy5jaGFyYWN0ZXIoKQ0KfQ0KDQpgYGANCg0KIyMgRXhlY3V0ZSBHZW5leHBpIGFuZCBURC1BcmFjbmUNCmBgYHtyIHJlc3VsdHM9J2hpZGUnfQ0KDQpmb3IocmVndWxhdG9yX3RvX3Rlc3QgaW4gcmVndWxhdG9ycykgew0KICBjYXQoIj09PT09PSIsIHJlZ3VsYXRvcl90b190ZXN0LCAiPT09PT1cbiIpDQogIHJlZ3Vsb25fdG9fdGVzdCA8LSByZWd1bG9uc190b190ZXN0W1tyZWd1bGF0b3JfdG9fdGVzdF1dDQogIA0KICAjVGhpcyBpcyB0dSBzdXBwb3J0IHJlc3RhcnRzIG9mIHRoZSBjb21wdXRhdGlvbg0KICBpZihpcy5udWxsKHJlc3VsdHNfZ2VuZXhwaVtbcmVndWxhdG9yX3RvX3Rlc3RdXSkpIHsNCiAgICBldmFsdWF0aW9uX3JlcyA8LSBldmFsdWF0ZVJhbmRvbUZvclJlZ3Vsb24oZGV2aWNlU3BlY3MgPSBkZXZpY2VTcGVjcyxyYXdQcm9maWxlcyA9IGdkczM4X3Jhd190b190ZXN0LCByb3VuZHMgPSByYW5kb21Sb3VuZHMsIHJlZ3VsYXRvck5hbWUgPSByZWd1bGF0b3JfdG9fdGVzdCwgcmVndWxvbk5hbWVzID0gcmVndWxvbl90b190ZXN0LCBjaGVja0NvbnN0YW50U3ludGhlc2lzID0gRkFMU0UsIHRpbWUgPSB0aW1lX3Ntb290aCwgcmF3VGltZSA9IHRpbWVfcmF3LCByYW5kb21TY2FsZSA9IHJhbmRvbVNjYWxlLCByYW5kb21MZW5ndGggPSByYW5kb21MZW5ndGgsIHNwbGluZURGcyA9IHNwbGluZURGLCBzcGxpbmVJbnRlcmNlcHQgPSBUUlVFLCBlcnJvckRlZiA9IGVycm9yRGVmKQ0KICAgIHJlc3VsdHNfZ2VuZXhwaVtbcmVndWxhdG9yX3RvX3Rlc3RdXSA8LSBldmFsdWF0aW9uX3Jlcw0KICAgIHNhdmUuaW1hZ2UoImV2YWx1YXRpb25fc2FjaGFyb215Y2VzLlJEYXRhIikNCiAgfQ0KfQ0KDQpmb3IocmVndWxhdG9yX3RvX3Rlc3QgaW4gcmVndWxhdG9ycykgew0KICBjYXQoIj09PT09PSBBcmFjbmU6IiwgcmVndWxhdG9yX3RvX3Rlc3QsICI9PT09PVxuIikNCiAgcmVndWxvbl90b190ZXN0IDwtIHJlZ3Vsb25zX3RvX3Rlc3RbW3JlZ3VsYXRvcl90b190ZXN0XV0NCiAgI1RoaXMgaXMgdHUgc3VwcG9ydCByZXN0YXJ0cyBvZiB0aGUgY29tcHV0YXRpb24NCiAgaWYoaXMubnVsbChyZXN1bHRzX2FyYWNuZVtbcmVndWxhdG9yX3RvX3Rlc3RdXSkpIHsNCiAgICByZXNfYXJhY25lIDwtIGV2YWx1YXRlVERBcmFjbmUocmFuZG9tUm91bmRzLCBnZHMzOF9yYXcsIHRpbWVfcmF3LCBjKHNwbGluZURGKSwgdGltZV9zbW9vdGgsIHJhbmRvbVNjYWxlLCByYW5kb21MZW5ndGgsIHJlZ3VsYXRvck5hbWUgPSByZWd1bGF0b3JfdG9fdGVzdCwgcmVndWxvbk5hbWVzID0gcmVndWxvbl90b190ZXN0LCBlcnJvckRlZiA9IGVycm9yRGVmLCBudW1CaW5zID0gMTApDQogICAgcmVzdWx0c19hcmFjbmVbW3JlZ3VsYXRvcl90b190ZXN0XV0gPC0gcmVzX2FyYWNuZQ0KICAgIHNhdmUuaW1hZ2UoImV2YWx1YXRpb25fc2FjaGFyb215Y2VzLlJEYXRhIikNCiAgfQ0KICANCn0NCmBgYA0KDQojIyBPdXRwdXQgdGhlIHJlc3VsdHMNCg0KRm9yIHRoZSBwdWJsaWNhdGlvbiwgd2UgY2hvc2UgdGhlIGJldHRlciByZXN1bHQgZm9yIFRELUFyYWNuZSAoZWl0aGVyIGRvd25zdHJlYW0gb3IgY29ubmVjdGVkKSBzZXBhcmF0ZWx5IGZvciBlYWNoIHJlZ3VsYXRvci4NCg0KYGBge3J9DQpyZXN1bHRzX2Zvcm1hdHRlZCA8LSBsaXN0KCkNCnN0ZXAgPSAxDQpmb3IocmVndWxhdG9yIGluIHJlZ3VsYXRvcnMpIHsNCiAgaWYoIWlzLm51bGwocmVzdWx0c19nZW5leHBpW1tyZWd1bGF0b3JdXSkpIHsNCiAgICByZXNfZ2VuZXhwaSA8LSByZXN1bHRzX2dlbmV4cGlbW3JlZ3VsYXRvcl1dDQogICAgcmVzdWx0c19mb3JtYXR0ZWRbW3N0ZXBdXSA8LSBkYXRhLmZyYW1lKHJlZ3VsYXRvciA9IHJlZ3VsYXRvciwgdHlwZSA9ICJHZW5leHBpIiwgdHJ1ZSA9IHJlc19nZW5leHBpJHRydWVSYXRpbywgcmFuZG9tID0gbWVhbihyZXNfZ2VuZXhwaSRyYW5kb21SYXRpb3MpKQ0KICAgIHN0ZXAgPC0gc3RlcCArIDENCiAgfQ0KICANCiAgaWYoIWlzLm51bGwocmVzdWx0c19hcmFjbmVbW3JlZ3VsYXRvcl1dKSkgew0KICAgIHJlc19hcmFjbmUgPC0gcmVzdWx0c19hcmFjbmVbW3JlZ3VsYXRvcl1dDQogICAgcmVzdWx0c19mb3JtYXR0ZWRbW3N0ZXBdXSA8LSBkYXRhLmZyYW1lKHJlZ3VsYXRvciA9IHJlZ3VsYXRvciwgdHlwZSA9ICJURC1BcmFjbmUtRG93bnN0cmVhbSIsIHRydWUgPSByZXNfYXJhY25lJHRydWVSYXRpb0Rvd25zdHJlYW0sIHJhbmRvbSA9IHJlc19hcmFjbmUkb3ZlcmFsbFJhbmRvbVJhdGlvRG93bnN0cmVhbSkNCiAgICBzdGVwIDwtIHN0ZXAgKyAxDQogICAgcmVzdWx0c19mb3JtYXR0ZWRbW3N0ZXBdXSA8LSBkYXRhLmZyYW1lKHJlZ3VsYXRvciA9IHJlZ3VsYXRvciwgdHlwZSA9ICJURC1BcmFjbmUtQ29ubmVjdGVkIiwgdHJ1ZSA9IHJlc19hcmFjbmUkdHJ1ZVJhdGlvQ29ubmVjdGVkLCByYW5kb20gPSByZXNfYXJhY25lJG92ZXJhbGxSYW5kb21SYXRpb0Nvbm5lY3RlZCkNCiAgICBzdGVwIDwtIHN0ZXAgKyAxDQogIH0NCn0NCg0KcmVzdWx0c19mb3JtYXR0ZWQgPC0gZG8uY2FsbChyYmluZCwgcmVzdWx0c19mb3JtYXR0ZWQpDQpyZXN1bHRzX2Zvcm1hdHRlZA0KYGBgDQoNCg==
